# Synthetic Study of Natural Metabolites Containing a Benzo[*c*]oxepine Skeleton: Heterocornol C and D

**DOI:** 10.3390/ijms241210331

**Published:** 2023-06-19

**Authors:** Ján Gettler, Tomáš Čarný, Martin Markovič, Peter Koóš, Erika Samoľová, Ján Moncoľ, Tibor Gracza

**Affiliations:** 1 Department of Organic Chemistry, Institute of Organic Chemistry, Catalysis and Petrochemistry, Slovak University of Technology, Radlinského 9, SK-812 37 Bratislava, Slovakia; xgettler@stuba.sk (J.G.); tomas.carny@uniba.sk (T.Č.); tibor.gracza@stuba.sk (T.G.); 2 Georganics Ltd., Koreničova 1, SK-811 03 Bratislava, Slovakia; 3 Institute of Physics of the Czech Academy of Science, Na Slovance 2, 182 21 Prague, Czech Republic; samolova@fzu.cz; 4 Department of Inorganic Chemistry, Institute of Inorganic Chemistry, Technology and Materials, Slovak University of Technology, Radlinského 9, SK-812 37 Bratislava, Slovakia; jan.moncol@stuba.sk

**Keywords:** heterocornol, polyketide, benzo[*c*]oxepin-1-one, natural compound, asymmetric synthesis

## Abstract

A versatile strategy for the enantioselective synthesis of a benzo[*c*]oxepine structural core containing natural secondary metabolites was developed. The key steps of the synthetic approach include ring-closing alkene metathesis for seven-member ring construction, the Suzuki–Miyaura cross-coupling reaction for the installation of the double bond and Katsuki–Sharpless asymmetric epoxidation for the introduction of chiral centers. The first total synthesis and absolute configuration assignment of heterocornol D (**3a**) were achieved. Four stereoisomers, **3a**, *ent*-**3a**, **3b** and *ent*-**3b**, of this natural polyketide were prepared, starting with 2,6-dihydroxy benzoic acid and divinyl carbinol. The absolute and relative configuration of heterocornol D was assigned via single-crystal X-ray analysis. The extension of the described synthetic approach is further presented with the synthesis of heterocornol C by applying the ether group reduction method to the lactone.

## 1. Introduction

**Figure 1:**
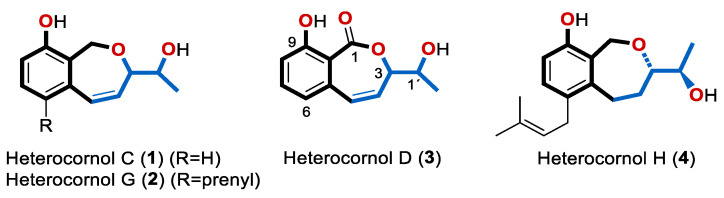
Structure of the secondary metabolites **1**–**4** from *Pestalotiopsis heterocornis*.

Marine organisms are interesting sources of new biologically and therapeutically active compounds [1]. Over the last several decades, many polyketide-type secondary metabolites of fungal origin have been evaluated [2]. Polyketides containing a salicylic/benzylic alcohol structural fragment have been shown to exhibit diverse biological activities [3,4,5,6,7]. Recently, Huang, Han and coworkers [8] described twelve new salicylaldehyde-type polyketide derivatives obtained from a marine-sponge-associated fungus *Pestalotiopsis heterocornis*. Among these compounds, bicyclic benzo[*c*]oxepines such as heterocornols C, D, G (**1**–**3**) and H (**4**) were identified (Figure 1).

The structure of natural compounds **1**–**4** was determined based on detailed spectroscopic data. The configuration of the C-3 and C-1′ centers in heterocornol H (**4**) were identified as anti by comparing the ^13^C NMR data of the putative structure of xylarinol B [8,9]. In addition, all the isolated metabolites were evaluated via MTT assay for their cytotoxic activity against four human cancer cell lines (BGC-823, H460, PC-3 and SMMC-7721). Compounds **1**, **2** and **4** showed moderate cytotoxicity, with IC_50_ values in the 15–100 μM range, with adriamycin assayed as a positive control. The antifungal properties of the isolated derivatives appeared to be dependent on the presence of the pent-4-ene-2,3-diol fragment. Derivatives **1** and **4** showed moderate antifungal activity against *Candida parapsilosis* and *Cryptococcus neofromans* at a concentration of 100 μg/mL. The promising results of the preliminary SAR study of *P. heterocosnis* metabolites encouraged us to develop a synthetic strategy for these natural compounds containing a benzo[*c*]oxepine skeleton.

## 2. Results and Discussion

**Scheme 1:**
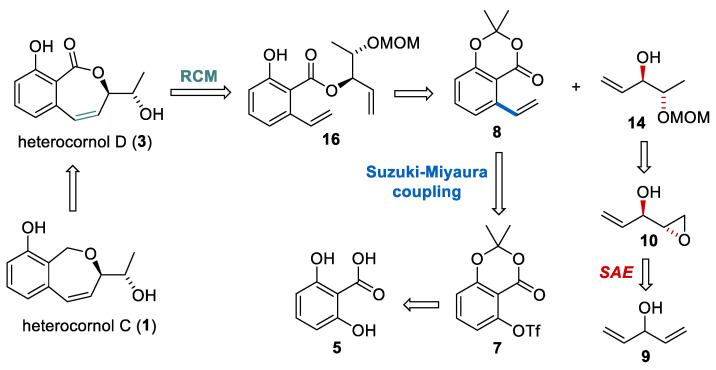
Retrosynthetic analysis of the heterocornols C and D.

To determine the relative and absolute configurations of the isolated heterocornols (**1**–**4**), we propose a flexible strategy for the synthesis of all possible stereoisomers of **3**. Herein, we report a synthetic route for the construction of the benzo[*c*]oxepine skeleton. The synthesis of all stereoisomers of heterocornol D (**3**) (**3a**, **3b**, *ent*-**3a** and *ent*-**3b**) with a relative C3-C1′ *syn*-/*anti*-configuration is described. The utilized strategy is depicted in Scheme 1.

In general, the key operation in synthesis is the construction of an oxepine-1-one ring via ring-closing metathesis. The substrate, optically pure diene **16**, having the defined configuration of the alkyl fragment, can be obtained via the re-esterification of the aromatic ester **8** with partially protected diol **14**. The following styrene **8** can be prepared via Suzuki–Miyaura coupling starting with triflate **7**, which, in turn, is available from 2,6-dihydroxy-benzoic acid **5**. The applicability of this strategy in the preparation of all stereoisomers of the targeted natural products is ensured through the Sharpless asymmetric epoxidation of divinyl carbinol **9** [10,11] and the eventual Mitsunobu inversion of the epoxide **10**, affording both diastereomers *syn* and *anti*, respectively.

**Scheme 2:**
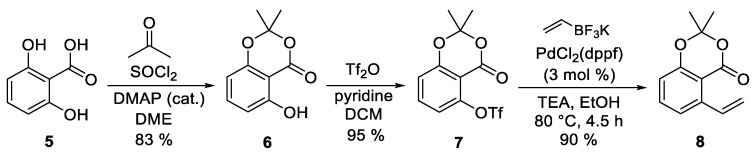
Synthesis of styrene **8**.

In addition, the proposed strategy could be utilized in the preparation of other natural heterocornols, (**1**), (**2**) and (**4**), involving selective reduction of the lactone **3**, the introduction of the prenyl group at carbon C-6 and/or hydrogenation of the C4-C5 double bond. Consequently, styrene **8** was readily prepared from commercially available 2,6-dihydroxy-benzoic acid **5** in three steps, using the literature protocols (Scheme 2) [12].

**Scheme 3:**
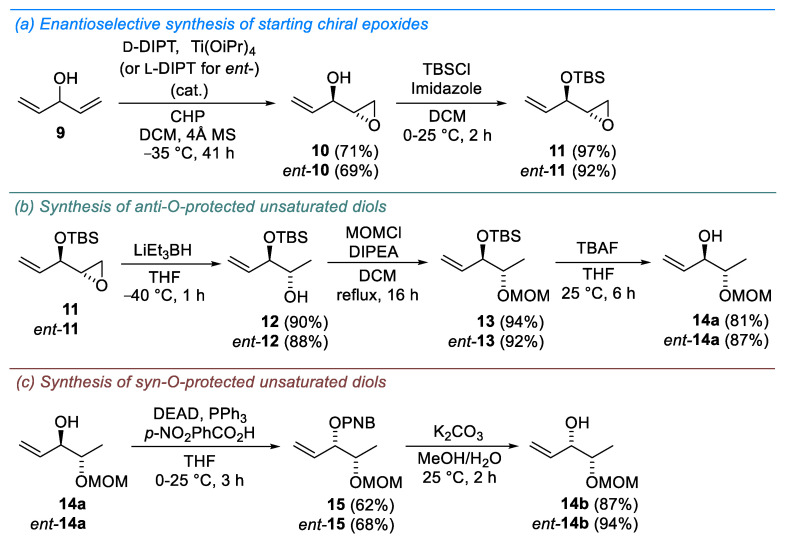
Synthesis of partially MOM-protected diols **14a**, **14b**, *ent*-**14a** and *ent*-**14b**.

The synthesis of the alkyl fragments, utilizing Katsuki–Sharpless asymmetric epoxidation (SAE), was employed for the desymmetrization of partially MOM-protected diols **14**, starting with commercially available prochiral divinyl carbinol **9** (Scheme 3a) [10,11,12,13]. Based on our previous work [14], we showed that *anti*-diastereomeric epoxides **10** and *ent*-**10** are readily available via SAE using cumene hydroperoxide (CHP) as an oxidant in good yields and high enantiopurities (71%/>99% ee and 69%/>99% ee, respectively) [15]. See Supplementary Materials for HPLC analysis (Figures S70–S73). All subsequent steps in the syntheses of both enantiomers, **14a**/*ent*-**14a**, were carried out in parallel.

As follows, the protection of the free hydroxyl group of **10**/*ent*-**10** with tert-butyl-dimethyl chlorosilane and imidazole in dichloromethane produced **11**/*ent*-**11** in a 97%/92% yield (Scheme 3a). Following the reduction of epoxides **11**/*ent*-**11** with lithium-triethyl-borohydride-furnished silyl-protected diols **12**/*ent*-**12** (90%/88%) (Scheme 3b), the first key fragments—compounds **14a**/*ent*-**14a**—were obtained in a two-step sequence. First, the protection of the C2-OH group of **12**/*ent*-**12** with methoxymethyl chloride was performed, followed by removal of the TBS-protecting group using TBAF in tetrahydrofuran. This sequence provided partially MOM-protected *anti*-diols **14a** and *ent*-**14a** in 76% and 75% yields (over two steps), respectively. The diastereomeric *syn*-diols **14b**/*ent*-**14b** were prepared from *anti*-diols **14a**/*ent*-**14a** using the Mitsunobu inversion reaction at the C3 centers (Scheme 3c). Thus, the treatment of **14a**/*ent*-**14a** with the mixture of *p*-nitrobenzoic acid, diethyl azodicarboxylate and triphenylphosphine produced fully protected diols **15**/*ent*-**15**, which, upon subsequent basic hydrolysis with potassium carbonate, afforded *syn*-diastereomers **14b** and *ent*-**14b** in good yields (54% and 64% over two steps).

**Scheme 4:**
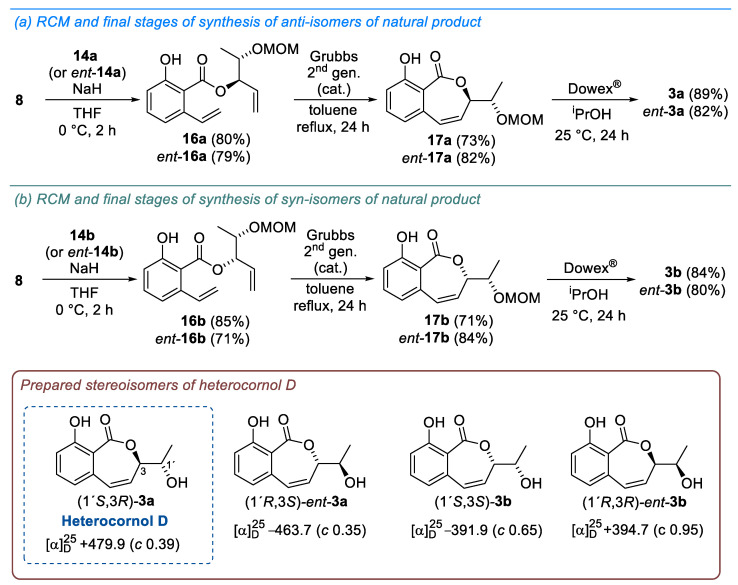
Synthesis of heterocornol D (**3a** and its isomers, **3b**, *ent*-**3a** and *ent*-**3b**).

With both key fragments in hand, the aromatic and alkyl fragments were coupled using a re-esterification reaction. In this manner, the reaction of the previously prepared styrene **8** with the corresponding alcohols **14** and sodium hydride in THF at 0 °C for 2 h provided the benzoates **16** in good yields (**16a**/80%, *ent*-**16a**/79%, **16b**/85%, *ent*-**16b**/71%), after work-up and purification using MPLC (Scheme 4a,b). The key reaction for the construction of the benzoxepinone skeleton was ring-closing alkene metathesis. Thus, the treatment of the dienes **16** with the Grubbs catalyst (second generation, 0.13 equiv) in toluene at reflux for 24 h provided benzoxepine-1-ones **17a**, *ent*-**17a**, **17b** and *ent*-**17b** in 73%, 82%, 71% and 84% yields, respectively. The final deprotection of the methoxymethyl group via acidic hydrolysis using Dowex in iso-propanol furnished the target heterocornols (**3**). The purification of the crude products via MPLC (5 min gradient from Hex to Hex/EtOAc = 75/25) provided the desired products **3a** (89%, $[\alpha {]}_{D}^{25}$ + 479.9 (c 0.39, MeOH)), *ent*-**3a** (82%, $[\alpha {]}_{D}^{25}$ − 463.7 (c 0.35, MeOH)), **3b** (84%, $[\alpha {]}_{D}^{25}$ − 391.9 (c 0.65, MeOH)) and *ent*-**3b** (80%, $[\alpha {]}_{D}^{25}$ + 394.7 (c 0.95, MeOH)) as colorless oils solidifying at −3 °C (Scheme 4).

**Figure 2:**
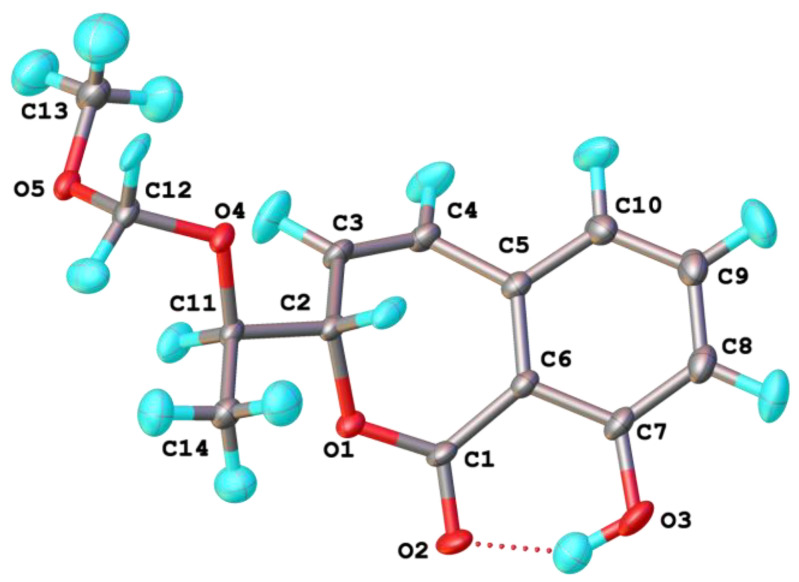
An ORTEP view of the *ent*-**17a** structure [16].

The following NMR analysis of the prepared final compounds showed very good agreement of the ^1^H and ^13^C spectral data for the synthetic derivatives **3a** and *ent*-**3a** with those obtained from the natural source, confirming the constitution of heterocornol D (**3**). The relative and absolute configurations of natural compound **3** were confirmed in a single-crystal X-ray analysis. See Supplementary Materials for X-ray data (Figures S1–S3, Tables S1 and S2). The colorless needles suitable for single-crystal X-ray analysis were isolated after MPLC purification and recrystallisation of the intermediate *ent*-**17a** (EtOAc/*n*-heptane = 1/25). Accordingly, the benzo[*c*]oxepin-1-one structure with 1′,3-*anti*-alignment and (1′*R*,3*S*)-absolute configuration of the compound *ent*-**17a** were confirmed (Figure 2) [16]. Considering the positive sign of the rotation of the plane of polarized light for the stereoisomer **3a**, the structure of the natural heterocornol D (**3**) was assigned as having 1′,3-*anti*-alignment. The (1′*S*,3*R*)-absolute configuration was also established, despite the fact that the value of specific rotation $[\alpha {]}_{D}^{25}$ + 479.9 (c 0.39, MeOH) of the synthetic sample **3a** differed from the literature value $[\alpha {]}_{D}^{20}$ + 75 (c 0.20, MeOH) [8] of natural **3**.

**Figure 3:**
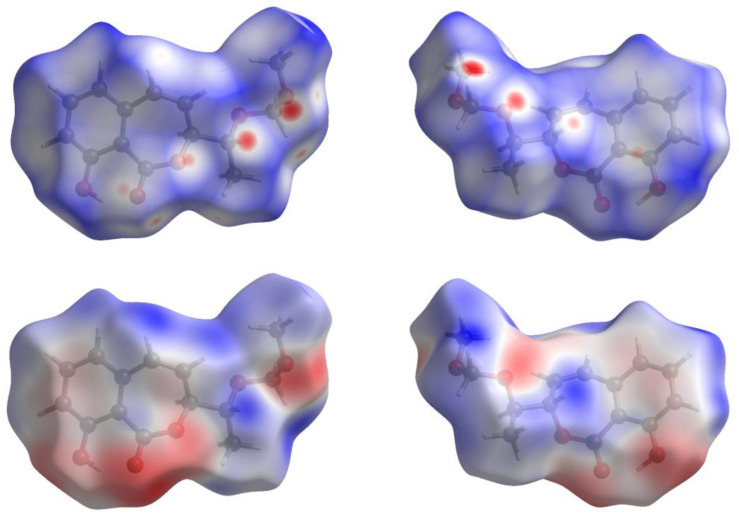
View of the three-dimensional Hirshfeld surface of *ent*-**17a**, plotted over d_norm_ in the range −0.1978 to 1.2511 a.u. (**top**), and the electrostatic potential on the Hirshfeld surface (**bottom**).

Moreover, the compound *ent*-**17a** was evaluated for a three-dimensional Hirshfeld surface. Furthermore, the calculated electrostatic potential on the Hirshfeld surface is shown in Figure 3.

The deep-red spots on the d_norm_ Hirshfeld surfaces indicate close contact interactions, which are mainly responsible for the significant intermolecular C–H∙∙∙O hydrogen-bonding interactions. The red and blue colors on the electrostatic potential of the Hirshfeld surface show the donor (red) and acceptor (blue) parts of the molecule for the formation of intermolecular interactions. The results of the electrostatic potential on the Hirshfeld surface confirm the existence of C–H∙∙∙O hydrogen-bonding interactions in the crystal structure of *ent*-**17a**. The analysis of the experimentally obtained Hirshfeld surface of *ent*-**17a** directly indicates the capacity for intermolecular interactions in future docking analysis.

**Scheme 5:**
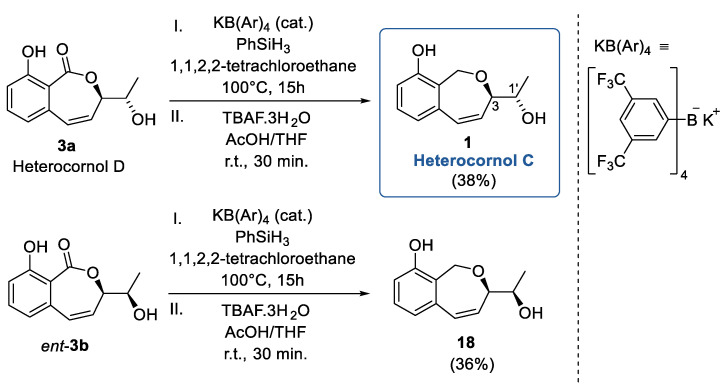
Synthesis of heterocornol C (**1**) and its isomer **18**.

To further extend the applicability of the described synthetic approach, an additional investigation for the synthesis of heterocornol C (**1**) was carried out. Since the relative and absolute configurations of natural compound **1** have not been described in the literature, we aimed to prepare both *syn* and *anti*-isomers of heterocornol C (**1**). Thus, synthesis was proposed utilizing the lactone reduction method, starting with the synthetic heterocornol D (**3a**). After trialing a few methods and optimization steps, we succeeded in the reduction of the lactone group of **3a** to the ether **1** using a modified literature method (this is an extension to substrates having free OH groups) [17]. The two-step procedure, utilizing potassium tetrakis[3,5-bis(trifluoromethyl)phenyl]borate as a catalyst and phenylsilane as a reducing agent, following treatment of the reaction mixture with TBAF, provided natural compound **1** in a 38% yield (Scheme 5).

Subsequently, the configuration of natural **1** was established through a comparison of the NMR data and optical rotation of synthetic **1** with the reported data of the isolated natural compound. See Supplementary Materials for NMR (Figures S4–S69, Tables S3 and S4). The sign and value of specific rotation $[\alpha {]}_{D}^{25}$ + 57.5 (c 0.3, MeOH) of the synthetic sample **1** and perfect match of the NMR spectra confirmed the *anti*-1′*S*,3*R* configuration of the natural heterocornol C (**1**) (see [8]: $[\alpha {]}_{D}^{20}$ + 80 (c 0.30, MeOH)).

To reliably prove the relative configuration of **1**, we also prepared its diastereomer **18b** in a 36% yield by applying the same synthetic procedure. The comparison of the NMR data of the synthetic *syn*-isomer **18b** with the natural **1** data showed large discrepancies in the shifts of the oxepine ring and C-1′ and C-3 proton signals in the ^1^H NMR spectrum, thus confirming the *anti*-alignment of the substituents of natural **1**.

## 3. Materials and Methods

Commercial materials which were obtained from Merck (https://www.sigmaaldrich.com/, Bratislava, Slovakia, accessed on 16 June 2023), Alfa Aesar (https://www.alfa.com/, curently: Thermo Fisher, Kandel, Germany, accessed on 16 June 2023) or Thermo Fisher Scientific (https://www.thermofisher.com/, Waltham, MA USA, accessed on 16 June 2023) were used without further purification. Reactions were monitored using TLC on silica gel. Compound purification was undertaken by flash chromatography. All solvents were distilled before use. Hexanes refer to the fraction boiling at 60–65 °C.

Melting points were obtained using a Boecius apparatus and are uncorrected. Optical rotations were measured with a JASCO P-2000 polarimeter and are given in units of 10^−1^ deg·cm^2^·g^−1^. FTIR spectra were obtained using a Nicolet 5700 spectrometer (Thermo Electron, currently: Thermo Fisher Scientific, Waltham, MA, USA) equipped with a Smart Orbit (diamond crystal ATR) accessory using the reflectance technique (400–4000 cm^−1^). ^1^H and ^13^C NMR spectra were recorded using either a 300 (75) MHz Unity Inova or a 600 (151) MHz VNMRS spectrometer from Varian. Standard chemical shifts are referenced to the corresponding solvent residual peaks (CDCl_3_: δ_H_ 7.26 ppm, δ_C_ 77.16 ppm; CD_3_OD: δ_H_ 3.31 ppm, δ_H_ 49.00 ppm; DMSO-d_6_: δ_H_ 2.50 ppm, δ_H_ 39.52 ppm) or tetramethylsilane (TMS) as an internal standard. High-resolution mass spectra (HRMS) were recorded with an OrbitrapVelos mass spectrometer (Thermo Scientific, currently: Thermo Fisher Scientific, Waltham, MA, USA) with a heated electrospray ionization (HESI) source. The mass spectrometer was operated with full scan (50–2000 amu) in the positive or negative FT mode (at a resolution of 100,000). The analyte was dissolved in MeOH and infused via a syringe pump at a rate of 5 mL/min. The heated capillary was maintained at 275 °C with a source heater temperature of 50 °C, and the sheath, auxiliary, and sweep gases were used at 10, 5, and 0 units, respectively. The source voltage was set to 3.5 kV. Flash column liquid chromatography (FLC) was undertaken on silica gel Kieselgel 60 (40–63 μm, 230–400 mesh), and analytical thin-layer chromatography (TLC) was performed on aluminum plates pre-coated with either 0.2 mm (DC-Alufolien, Merck Life Science, Bratislava, Slovakia) or 0.25 mm silica gel 60 F254 (ALUGRAM SIL G/UV254, Macherey-Nagel, Dueren, Germany). The compounds were visualized using UV fluorescence and by dipping the plates into an aqueous H_2_SO_4_ solution of cerium sulfate/ammonium molybdate, followed by charring with a heat gun.

The data collection and cell refinement of *ent*-**17a** were performed with a SuperNova diffractometer using a CCD detector Atlas S2 and a micro-focus sealed tube with mirror-collimated CuKα radiation (λ = 1.54184 Å). The structure was solved using the Superflip program and refined using the full-matrix least-squares procedure of the Independent Atom Model (IAM) with Shelxl (ver. 2018/3) [18,19]. The Hirshfeld Atom Refinement (HAR) method was carried out using the IAM model as a starting point. The wave function was calculated using ORCA 4.2.0 software with the basic set def2-TZVP and hybrid exchange–correlation functional PBE0 [20,21,22]. The least-squares refinement of the HAR model was then carried out with Olex2.refine (ver. 1.5-alpha) [23]. The NoSpherA2 implementation of HAR was used for tailor-made aspherical atomic factors calculated on-the-fly using a Hirshfeld-partitioned electron density [24]. For the HAR approach, all hydrogen atoms were accurately anisotropic, using restraints on the X-H distances of the neutron structures [25]. The structure was drawn using the OLEX2 package [26]. The absolute configuration of the *ent*-**17a** stereoisomer was determined using the Parsons and Hooft methods [27,28]. The software CrystalExplorer [29] (version 21.5) was used to calculate the Hirshfeld surface, electrostatic potentials, and associated fingerprint plots [30,31,32].

## 4. Conclusions

In summary, we developed a synthetic strategy for the enantioselective construction of a benzo[*c*]oxepine framework of natural *Pestalotiopsis heterocornis* metabolites. The key features of the strategy employ the ring-closing metathesis of diene for the assembly of the oxepinone ring and the Sharpless asymmetric epoxidation of allyl alcohol. The applicability of this approach was demonstrated through the synthesis of naturally occurring heterocornol D. Four stereoisomers of this secondary metabolite were prepared from prochiral divinyl carbinol and 2,2-dihydroxy benzoic acid. The absolute configuration of the natural stereoisomer was established based on the single-crystal X-ray analysis of the intermediate *ent*-**17a**. The (*R*)-9-hydroxy-3-((*S*)-1′-hydroxyethyl)benzo[*c*]oxepin-1(3H)-one (+)-**3a** was identified as the naturally occurring heterocornol D (**3**) based on the comparison of the specific rotation and NMR spectroscopic data. In addition, the synthesis of heterocornol C (**1**) was achieved by extending the synthetic route using the lactone group reduction method with the prepared heterocornol D (**3**). Finally, the presented approach can provide access to other natural benzo[*c*]oxepine-derived polyketides that show improved properties for their evaluation as potential drugs.

## Data Availability

The datasets supporting the conclusions of this article are included within the article and Supplementary Materials.

## Supplementary Materials

The following supporting information can be downloaded at: https://www.mdpi.com/article/10.3390/ijms241210331/s1. All experimental procedures and analytical data for all compounds, copies of ^1^H and ^13^C NMR spectra, HPLC analysis of compounds and crystallographic data are included.
