# Supplementary material for: Synthetic Study of Natural Metabolites Containing a Benzo[c]oxepine Skeleton: Heterocornol C and D
Source: Int J Mol Sci. 2023 Jun 19;24(12):10331. doi: 10.3390/ijms241210331 (PMC10299168; doi:10.3390/ijms241210331)
Supplement: Supplementary file 1 [file ijms-24-10331-s001.zip › Experimental procedures and analytical data_heterocornol D.pdf]

Supporting information – Experimental procedures and analytical data

Synthetic Study of Natural Metabolites Containing a Benzo[*c*]oxepine Skeleton: Heterocornol C and D

Ján Gettler,† Tomáš Čarný, † Martin Markovič,†‡ Peter Koóš,* †‡ Erika Samoľová,# Ján Moncol§ and Tibor Gracza†*

†Department of Organic Chemistry, Institute of Organic Chemistry, Catalysis and Petrochemistry, Slovak University of Technology, Radlinského 9, SK-812 37 Bratislava, Slovakia

‡ Georganics Ltd., Koreničova 1, SK-811 03 Bratislava, Slovakia

Institute of Physics of the Czech Academy of Science, Na Slovance 2, 182 21 Prague 8, Czech Republic

§Department of Inorganic Chemistry, Institute of Inorganic Chemistry, Technology and Materials, Slovak University of Technology, Radlinského 9, SK-812 37 Bratislava, Slovakia

Table of Content

General Experimental Procedures…………………………………………………………S3

Experimental procedures and analytical data for compounds:

Experimental procedure for **8** ……………………..…..……………………..……………......... S4
Experimental procedure for **10** ………………………………...…………………….……..…. S4
Experimental procedure for *ent*-**10** ………………………...……………………..……..…. S5
Experimental procedure for **11** ………………………………...…………………….……..…. S5
Experimental procedure for *ent*-**11** ………………………...……………………..……..…. S6
Experimental procedure for **12** ………………………………...…………………….……..…. S6
Experimental procedure for *ent*-**12** ………………………...……………………..……..…. S7
Experimental procedure for **13** ………………………………...…………………….……..…. S7
Experimental procedure for *ent*-**13** ………………………...……………………..……..…. S8
Experimental procedure for **14a** ……………………………...……………….…………..…. S8
Experimental procedure for *ent*-**14a**…………………………...…………………….……..…. S9
Experimental procedure for **15** ………………………………...…………………….……..…. S9
Experimental procedure for *ent*-**15** …………………………....………………..……....…. S10
Experimental procedure for **14b**…………………………...…..………………...………..…. S10
Experimental procedure for *ent*-**14b**……………………...……...……………………….. S11
Experimental procedure for **16a**…...…………………………...………………………..……. S11
Experimental procedure for *ent*-**16a**…………………………...…………………….………. S12
Experimental procedure for **16b** …………………………………..………………………….. S13
Experimental procedure for *ent*-**16b**……...……………………...…………………..…..... S13
Experimental procedure for **17a** ……..……………………………...……………….……. S14
Experimental procedure for *ent*-**17a**……....……………………...…………………..…..... S15
Experimental procedure for **17b** ……………………………………...………………….….…S15
Experimental procedure for *ent*-**17b**……....……………………...…………………..…..... S16
Experimental procedure for **3a** ………………………..………...…………………….……. S16
Experimental procedure for *ent*-**3a**……...……………..…...…………………….……. S17
Experimental procedure for **3b** ………………………..………...…………………….……. S18
Experimental procedure for *ent*-**3b**……...……………..……...…………………….……. S18
Experimental procedure for **1** ……………………..…..……………………..……………........S19
Experimental procedure for **18** …...……………………………....…………………..……..... S20

General Experimental Procedures:

Melting points were obtained using a Boecius apparatus and are uncorrected. Optical rotations were measured with a JASCO P-2000 polarimeter and are given in units of 10^{-1} deg.cm^2.g^{-1}. FTIR spectra were obtained on a Nicolet 5700 spectrometer (Thermo Electron) equipped with a Smart Orbit (diamond crystal ATR) accessory, using the reflectance technique (4000-400 cm-1). ^{1}H and ^{13}C NMR spectra were recorded on either a 300 (75) MHz Unity Inova or a 600 (151) MHz VNMRS spectrometer from Varian. Standard chemical shifts are referenced to corresponding solvent residual peak ($CDCl_3$: δ_H 7.26 ppm, δ_C 77.16 ppm; CD_3OD: δ_H 3.31 ppm, δ_C 49.00 ppm; DMSO-d_6: δ_H 2.50 ppm, δ_C 39.52 ppm) or tetramethylsilane (TMS) as internal standard. High resolution mass spectra (HRMS) were recorded on an OrbitrapVelos mass spectrometer (Thermo Scientific) with a heated electrospray ionisation (HESI) source. The mass spectrometer was operated with full scan (50–2000 amu) in positive or negative FT mode (at a resolution of 100,000). The analyte was dissolved in MeOH and infused via syringe pump at a rate of 5 mL/min. The heated capillary was maintained at 275 °C with a source heater temperature of 50 °C and the sheath, auxiliary and sweep gases were at 10, 5 and 0 units, respectively. Source voltage was set to 3.5 kV. Flash column liquid chromatography (FLC) was performed on silica gel Kieselgel 60 (40-63 μm, 230-400 mesh) and analytical thin-layer chromatography (TLC) was performed on aluminum plates pre-coated with either 0.2 mm (DC-Alufolien, Merck) or 0.25 mm silica gel 60 F254 (ALUGRAM SIL G/UV254, Macherey-Nagel). The compounds were visualized by UV fluorescence and by dipping the plates in an aqueous H_2SO_4 solution of cerium sulfate/ammonium molybdate followed by charring with a heat gun. The absorption of crude material on a silica gel was performed after drying of a solution of crude material using Na_2SO_4 and filtration. Filtered solution of crude material was mixed with a certain amount of SiO_2 and it was evaporated under vacuum to dryness.

Experimental procedures and analytical data for compounds:

2,2-dimethyl-5-vinyl-4H-benzo[*d*][1,3]dioxin-4-one (8). To a solution of potassium vinyltrifluoroborate (1.13 g, 8.44 mmol, 1.1 equiv), $PdCl_2$(dppf).CH_2Cl_2 (188 mg, 0.23 mmol, 0.03 equiv) and TEA (1.39 mL, 1.01 g, 9.97 mmol, 1.3 equiv) in EtOH (50 mL) was added triflate **7** (2.50 g, 7.67 mmol, 1.0 equiv). The reaction mixture was allowed to reflux for 4.5 h under Ar atmosphere. The reaction mixture was than cooled to room temperature, diluted with water (50 mL) and aqueous layer was extracted by Et_2O (3 x 50 mL). Combined organic layers were washed by brine, dried over anhydrous Na_2SO_4, filtered, and adsorbed on silica gel. The residue was purified using MPLC (5 min. gradient from Hex/EtOAc: 100/ 0 to Hex/ EtOAc: 95/5) providing desired product **8** (1.41 g, 90% yield, white solid): TLC R_f 0.27 (Hex/EtOAc: 90/10); m.p. 49.4 – 50.2 °C; IR (ATR) ν_{max} 3087, 2989, 1720, 1475, 1202, 921, 695 cm^{-1}; NMR data were in good agreement with published data in ref [12]: ^{1}H NMR (300 MHz, $CDCl_3$) δ_H 7.72 (dd, *J* = 17.5, 11.0 Hz, 1 H), 7.46 (td, *J* = 8.0, 0.6 Hz, 1 H), 7.26 (ddd, *J* = 7.8, 1.1, 0.6 Hz, 1 H), 6.88 (dd, *J* = 8.2, 1.1 Hz, 1 H), 5.71 (dd, *J* = 17.4, 1.4 Hz, 1 H), 5.42 (dd, *J* = 11.0, 1.4 Hz, 1 H), 1.71 (s, 6 H) ppm; ^{13}C NMR (75 MHz, $CDCl_3$) δ_C 160.4, 156.9, 142.5, 135.5, 135.4, 121.5, 117.9, 116.7, 111.0, 105.4, 25.8 ppm; HRMS (ESI) *m/z* $[M + H]^+$ calcd for $C_{12}H_{13}O_3$: 205.0859, found: 205.0859; $[M + Na]^+$ calcd for $C_{12}H_{12}NaO_3$: 227.0679, found: 227.0679.

(*R*)-1-((*S*)-oxiran-2-yl)prop-2-en-1-ol (10). A mixture of crushed and activated 4Å molecular sieves (16 g) in dry DCM (240 mL) was cooled to – 40 °C under Ar atmosphere. To the mixture was then added $Ti(O^iPr)_4$ (7.03 mL, 6.76 g, 23.78 mmol, 0.1 equiv) and D-DIPT (6.62 mL, 7.41 g, 31.62 mmol, 0.13 equiv). After 30 min. of stirring at – 35 °C divinyl carbinol **9** (23.12 mL, 20 g, 237.76 mmol, 1 equiv) was added, followed by addition of cumene hydroperoxide (70.26 mL, 72.37 g, 380.41 mmol, 1.6 equiv, 80 wt-%). The reaction mixture was stirred at – 35 °C for 41 h. Saturated Na_2SO_4 solution (20 mL), Et_2O (200 mL) and Celite (55 g) were added. After the mixture was left to stir at ambient temperature for 3 h, the resulting slurry was filtered through a pad of Celite and glass fiber paper and yellow solution was concentrated under

reduced pressure (min. 200 mbar). Excess cumene alcohol and cumene hydroperoxide were removed by MPLC (10 min. gradient from Hex/EtOAc: 100/0 to Hex/EtOAc: 80/20, then Et_2O). Distillation under reduced pressure provided the desire product **10** (16.79 g, 71 % yield, colorless oil): TLC R_f 0.27 (Hex/EtOAc: 75/25); $[\boldsymbol{\alpha}]_{\mathbf{D}}^{\mathbf{25}}$ –56.90 (*c* 2.19, $CHCl_3$), $[\alpha]_D^{25}$–61.0 (*c* 0.93, $CHCl_3$), Ref [13,15]; IR (ATR) ν_{max} 3406, 2995, 1645, 1427, 1251, 993, 885 cm^{-1}; ^1H NMR (300 MHz, $CDCl_3$) δ_H 5.85 (ddd, *J* = 17.3, 10.5, 6.2 Hz, 1 H), 5.40 (dt, *J* = 17.3, 1.3 Hz, 1 H), 5.27 (dt, *J* = 10.5, 1.2 Hz, 1 H), 4.38 – 4.32 (m, 1 H), 3.10 (ddd, *J* = 6.9, 3.7, 3.0 Hz, 1 H), 2.81 (dd, *J* = 4.9, 3.0 Hz, 1 H), 2.76 (dd, *J* = 4.9, 3.8 Hz, 1 H), 2.00 (d, *J* = 2.8 Hz, 1 H) ppm; ^{13}C NMR (75 MHz, $CDCl_3$) δ_C 135.6, 117.8, 70.3, 54.0, 43.6 ppm; HRMS (ESI) *m/z* $[M + Na]^+$ calcd for $C_5H_8NaO_2$: 123.0417, found: 123.0403.

(*S*)-1-((*R*)-oxiran-2-yl)prop-2-en-1-ol (*ent*-10). Prepared as described for enantiomer **10** from divinyl carbinol **9** (23.12 mL, 20 g, 237.76 mmol, 1 equiv), $Ti(O^iPr)_4$ (7.03 mL, 6.76 g, 23.78 mmol, 0.1 equiv) and L-DIPT (6.62 mL, 7.41 g, 31.62 mmol, 0.13 equiv). Distillation under reduced pressure provided epoxide *ent*-**10** (16.53 g, 69 % yield, colorless oil); TLC R_f 0.21 (Hex/EtOAc: 75/25); $[\boldsymbol{\alpha}]_{\mathbf{D}}^{\mathbf{25}}$ +52.33 (*c* 2.65, $CHCl_3$) $[\alpha]_D^{25}$ +59.6 (*c* 0.97, $CHCl_3$), Ref [13,15]; IR (ATR) ν_{max} 3420, 2996, 1645, 1427, 1104, 929, 885 cm^{-1}; ^1H NMR (300 MHz, $CDCl_3$) δ_H 5.85 (ddd, *J* = 17.3, 10.5, 6.2 Hz, 1 H), 5.40 (dt, *J* = 17.3, 1.3 Hz, 1 H), 5.27 (dt, *J* = 10.5, 1.3 Hz, 1 H), 4.35 (s, 1 H), 3.13 – 3.06 (m, 1 H), 2.81 (dd, *J* = 5.0, 0.5 Hz, 1 H), 2.76 (dd, *J* = 5.0, 4.0 Hz, 1 H), 1.99 (s, 1 H) ppm; ^{13}C NMR (75 MHz, $CDCl_3$) δ_C 135.6, 117.8, 70.3, 54.0, 43.6 ppm; HRMS (ESI) *m/z* $[M + Na]^+$ calcd for $C_5H_8NaO_2$: 123.0417, found: 123.0404.

***tert*-Butyldimethyl(((*R*)-1-((*S*)-oxiran-2-yl)allyl)oxy)silane (11).** To a solution of **10** (2.71 g, 27.09 mmol) in DCM (65 mL) was added imidazole (4.43 g, 65.01 mmol, 2.4 equiv). After cooling the solution to 0 °C, TBSCl (7.76 g, 51.47 mmol, 1.9 equiv) was added in one portion. The reaction mixture was stirred for 2 h at ambient temperature. After full conversion (indicated by TLC), reaction mixture was quenched by saturated $NaHCO_3$ solution (50 mL). Aqueous

layer was extracted by DCM (2 x 20 mL). Combined organic layers were dried over anhydrous Na_2SO_4, filtered, and concentrated under reduced pressure (min. 200 mbar). The residue was purified using MPLC (5 min. gradient from Hex/Et_2O: 100/0 to Hex/Et_2O: 99.5/0.5) providing desired product **11** (5.61 g, 97% yield, colorless oil); TLC R_f 0.33 (Hex/EtOAc: 95/5); $[\alpha]_D^{25}$ –1.53 (*c* 2.06, $CHCl_3$); IR (ATR) ν_{max} 2929, 2857, 1473, 1250, 1080, 1000, 834 cm^{-1}; ^1H NMR (300 MHz, $CDCl_3$) δ_H 5.88 (ddd, *J* = 17.2, 10.4, 5.6 Hz, 1 H), 5.32 (dt, *J* = 17.2, 1.6 Hz, 1 H), 5.20 (dt, *J* = 10.4, 1.6 Hz, 1 H), 4.12 (ddt, *J* = 5.6, 4.0, 1.4 Hz, 1 H), 2.98 – 2.91 (m, 1 H), 2.70 (d, *J* = 3.5 Hz, 2 H), 0.89 (s, 9 H), 0.06 (s, 3 H), 0.05 (s, 3 H) ppm; ^{13}C NMR (75 MHz, $CDCl_3$) δ_C 137.6, 116.1, 72.6, 54.7, 44.4, 25.9, 18.4, -4.6, -4.7 ppm; HRMS (ESI) *m/z* $[M + Na]^+$ calcd for $C_{11}H_{22}NaO_2Si$: 237.1281, found: 237.1282.

***tert*-Butyldimethyl(((*S*)-1-((*R*)-oxiran-2-yl)allyl)oxy)silane (*ent*-11)**. Prepared as described for **11** from *ent*-**10** (1.05 g, 10.49 mmol), imidazole (1.714 g, 25.17 mmol, 2.4 equiv), TBSCl (3.00 g, 19.93 mmol, 1.9 equiv) in DCM (25 mL). Epoxide *ent*-**11** (2.07 g, 92% yield, colorless oil); TLC R_f 0.33 (Hex/EtOAc: 95/5); $[\alpha]_D^{25}$ +1.21 (*c* 2.20, $CHCl_3$); IR (ATR) ν_{max} 2956, 2929, 2857, 1472, 1251, 834, 775 cm^{-1}; ^1H NMR (300 MHz, $CDCl_3$) δ_H 5.88 (ddd, *J* = 17.2, 10.4, 5.6 Hz, 1 H), 5.32 (dt, *J* = 17.2, 1.6 Hz, 1 H), 5.18 (dt, *J* = 10.4, 1.6 Hz, 1 H), 4.12 (ddt, *J* = 5.6, 4.0, 1.4 Hz, 1 H), 2.98 – 2.91 (m, 1 H), 2.71 (d, *J* = 3.3 Hz, 2 H), 0.89 (s, 9 H), 0.06 (s, 3 H), 0.05 (s, 3 H) ppm; ^{13}C NMR (75 MHz, $CDCl_3$) δ_C 137.6, 116.1, 72.6, 54.7, 44.4, 25.9, 18.4, -4.6, -4.7 ppm; HRMS (ESI) *m/z* $[M + H]^+$ calcd for $C_{11}H_{23}O_2Si$: 215.1462, found: 215.1461; $[M + Na]^+$ calcd for $C_{11}H_{22}NaO_2Si$: 237.1281, found: 237.1281.

(2*S*,3*R*)-3-((*tert*-Butyldimethylsilyl)oxy)pent-4-en-2-ol (12). A solution of **11** (5.22 g, 24.35 mmol) in dry THF (150 mL) under Ar atmosphere was cooled to -40 °C. $LiEt_3BH$ (17.19 mL, 29.22 mmol, 1.2 equiv, 1.7 M in THF) was added dropwise through septum and reaction mixture was left to stir for 1 h to full conversion (indicated by TLC). Reaction mixture was quenched by saturated NH_4Cl solution (50 mL). Aqueous layer was extracted by Et_2O (3 x 75 mL). Combined organic layers were dried over anhydrous Na_2SO_4, filtered, and concentrated

under reduced pressure (min. 200 mbar). The residue was purified using MPLC (7 min. gradient from Hex/Et_2O: 100/0 to Hex/Et_2O: 90/10) providing desired product **12** (4.75 g, 90% yield, colorless oil); TLC R_f 0.23 (Hex/EtOAc: 90/10); $[\alpha]_D^{25}$ +3.71 (*c* 1.32, $CHCl_3$); IR (ATR) ν_{max} 2956, 2930, 2858, 1472, 1252, 1077, 924 cm^{-1}; ^1H NMR (300 MHz, $CDCl_3$) δ_H 5.82 (ddd, *J* = 17.3, 10.5, 6.8 Hz, 1 H), 5.24 (ddd, *J* = 9.5, 1.8, 1.2 Hz, 1 H), 5.21 – 5.17 (m, 1 H), 4.02 – 3.97 (m, 1 H), 3.73 (qt, *J* = 6.4, 4.2 Hz, 1 H), 2.15 (d, *J* = 4.3 Hz, 1 H), 1.11 (d, *J* = 6.4 Hz, 3 H), 0.91 (s, 9 H), 0.08 (s, 3 H), 0.05 (s, 3 H) ppm; ^{13}C NMR (75 MHz, $CDCl_3$) δ_C 137.2, 117.2, 78.2, 70.8, 26.0, 18.3, 17.6, -4.1, -4.8 ppm; HRMS (ESI) *m/z* $[M + Na]^+$ calcd for $C_{11}H_{24}NaO_2Si$: 239.1438, found: 239.1438.

(2*R*,3*S*)-3-((*tert*-Butyldimethylsilyl)oxy)pent-4-en-2-ol (*ent*-12). Prepared as above from *ent*-**11** (1.79 g, 8.38 mmol), $LiEt_3BH$ (5.92 mL, 10.06 mmol, 1.2 equiv, 1.7 M in THF). Compound *ent*-**12** (1.59 g, 88% yield , colorless oil); TLC R_f 0.23 (Hex/EtOAc: 90/10); $[\alpha]_D^{25}$ -3.84 (*c* 1.70, $CHCl_3$); IR (ATR) ν_{max} 2956, 2930, 2858, 1463, 1252, 1024, 833 cm^{-1}; ^1H NMR (300 MHz, $CDCl_3$) δ_H 5.81 (ddd, *J* = 17.3, 10.5, 6.8 Hz, 1 H), 5.24 (ddd, *J* = 9.5, 1.8, 1.2 Hz, 1 H), 5.21 – 5.18 (m, 1 H), 4.04 – 3.95 (m, 1 H), 3.73 (qt, *J* = 6.4, 4.1 Hz, 1 H), 2.16 (d, *J* = 4.2 Hz, 1 H), 1.11 (d, *J* = 6.4 Hz, 3 H), 0.91 (s, 9 H), 0.08 (s, 3 H), 0.05 (s, 3 H) ppm; ^{13}C NMR (75 MHz, $CDCl_3$) δ_C 137.2, 117.2, 78.2, 70.8, 26.0, 18.3, 17.6, -4.1, -4.8 ppm; HRMS (ESI) *m/z* $[M + Na]^+$ calcd for $C_{11}H_{24}NaO_2Si$: 239.1438, found: 239.1443.

(5*S*,6*R*)-5,8,8,9,9-Pentamethyl-6-vinyl-2,4,7-trioxa-8-siladecane (13). To a solution of substrate **12** (2.73 g, 12.62 mmol) and DIPEA (3.3 mL, 2.45 g, 18.92 mmol, 1.5 equiv) in dry DCM (30 mL) under Ar atmosphere at 0 °C was added MOMCl (0.96 mL, 1.02 g, 12.62 mmol, 1 equiv). Reaction mixture was heated and refluxed for 16 h overnight. Reaction mixture was cooled to room temperature and quenched by saturated $NaHCO_3$ solution (50 mL). Aqueous layer was extracted by DCM (3 x 50 mL). Combined organic layers were dried over anhydrous Na_2SO_4, filtered, and concentrated under reduced pressure (min. 200 mbar). The residue was

purified using MPLC (5 min. gradient from Hex/DCM: 100/0 to Hex/DCM: 50/50) providing desired product **13** (3.10 g, 94% yield, colorless oil), TLC R_f 0.23 (Hex/Et_2O: 90/10); $[\alpha]_D^{25}$ – 13.10 (*c* 1.20, $CHCl_3$); IR (ATR) ν_{max} 2929, 2857, 1473, 1251, 1104, 1034, 920, 833 cm^{-1}; ^1H NMR (300 MHz, $CDCl_3$) δ_H 5.85 (ddd, *J* = 17.2, 10.4, 6.1 Hz, 1 H), 5.24 (ddd, *J* = 17.2, 1.9, 1.5 Hz, 1 H), 5.15 (ddd, *J* = 10.4, 1.9, 1.3 Hz, 1 H), 4.70 (d, *J* = 6.7 Hz, 1 H), 4.65 (d, *J* = 6.7 Hz, 1 H), 4.04 (ddt, *J* = 6.1, 4.2, 1.3 Hz, 1 H), 3.66 (qd, *J* = 6.4, 4.2 Hz, 1 H), 3.37 (3 H, s), 1.13 (d, *J* = 6.4 Hz, 3 H), 0.90 (s, 9 H), 0.06 (s, 3 H), 0.03 (s, 3 H) ppm; ^{13}C NMR (75 MHz, $CDCl_3$) δ_C 138.5, 116.0, 95.5, 77.1, 76.4, 55.5, 26.0, 18.4, 16.1, -4.3, -4.7 ppm; HRMS (ESI) *m/z* $[M + H]^+$ calcd for $C_{13}H_{29}O_3Si$: 261.1880, found: 261.1881; $[M + Na]^+$ calcd for $C_{13}H_{28}NaO_3Si$: 283.1700, found: 283.1701, $[M + K]^+$ calcd for $C_{13}H_{28}KO_3Si$: 299.1439, found: 299.1441.

(5*R*,6*S*)-5,8,8,9,9-Pentamethyl-6-vinyl-2,4,7-trioxa-8-siladecane (*ent*-13). Prepared as **13** from *ent*-**12** (990 mg, 4.58 mmol), DIPEA (4.78 mL, 3.55 g, 27.45 mmol, 3 equiv) MOMCl (0.69 mL, 740 mg, 9.15 mmol, 2 equiv) in dry DCM (11 mL). Compound *ent*-**13** (1.09 g, 92% yield, colorless oil); TLC R_f 0.23 (Hex/Et_2O: 90/10); $[\alpha]_D^{25}$ +10.96 (*c* 1.30, $CHCl_3$); IR (ATR) ν_{max} 2930, 1473, 1251, 1104, 1034, 833, 774 cm^{-1}; ^1H NMR (300 MHz, $CDCl_3$) δ_H 5.85 (ddd, *J* = 17.2, 10.4, 6.1 Hz, 1 H), 5.24 (ddd, *J* = 17.2, 1.9, 1.5 Hz, 1 H), 5.15 (ddd, *J* = 10.4, 1.9, 1.3 Hz, 1 H), 4.70 (d, *J* = 6.7 Hz, 1 H), 4.65 (d, *J* = 6.7 Hz, 1 H), 4.04 (ddt, *J* = 5.5, 4.2, 1.3 Hz, 1 H), 3.66 (qd, *J* = 6.4, 4.2 Hz, 1 H), 3.37 (3 H, s), 1.13 (d, *J* = 6.4 Hz, 3 H), 0.90 (s, 9 H), 0.06 (s, 3 H), 0.03 (s, 3 H); ^{13}C NMR (75 MHz, $CDCl_3$) δ_C 138.5, 116.0, 95.5, 77.1, 76.4, 55.5, 26.0, 18.4, 16.1, -4.3, -4.7 ppm; HRMS (ESI) *m/z* $[M + Na]^+$ calcd for $C_{13}H_{28}NaO_3Si$: 283.1700, found: 283.1703.

(3*R*,4*S*)-4-(Methoxymethoxy)pent-1-en-3-ol (14a). To a solution of substrate **13** (1.00 g, 3.84 mmol) in THF (38 mL) was added TBAF (1.57 g, 4.99 mmol, 1.5 equiv) and reaction

mixture was left to stir at room temperature. After 6 h of stirring (full conversion monitored by TLC), water (30 mL) was added and aqueous layer was extracted by Et_2O (3 x 50 mL). Combined organic layers were dried over anhydrous Na_2SO_4, filtered, and concentrated under reduced pressure (min. 200 mbar). The residue was purified using MPLC (5 min. gradient from Hex/Et_2O: 100/0 to Hex/Et_2O: 60/40) providing desired diol **14a** (460 mg, 81% yield, colorless oil), TLC R_f 0.20 (Hex/Et_2O: 50/50); $[\alpha]_D^{25}$ +65.6 (*c* 1.70, $CHCl_3$); IR (ATR) ν_{max} 2980, 2934, 1448, 1378, 1215, 1026, 986 cm^{-1}; ^1H NMR (300 MHz, $CDCl_3$) δ_H 5.88 (ddd, *J* = 17.3, 10.5, 5.9 Hz, 1 H, H-4), 5.33 (dt, *J* = 17.3, 1.6 Hz, 1 H), 5.24 (dt, *J* = 10.5, 1.6 Hz, 1 H), 4.71 (d, *J* = 6.9 Hz, 1 H), 4.69 (d, *J* = 6.9 Hz, 1 H),4.16 – 4.06 (m, 1 H), 3.78 (qd, *J* = 6.5, 3.0 Hz, 1 H), 3.40 (s, 3 H), 2.77 (d, *J* = 6.1 Hz, 1 H), 1.16 (d, *J* = 6.5 Hz, 3 H) ppm; ^{13}C NMR (151 MHz, $CDCl_3$) δ_C 136.4, 116.9, 96.2, 78.1, 75.3, 55.8, 15.7 ppm; HRMS (ESI) *m/z* $[M + H]^+$ calcd for $C_7H_{15}O_3$: 147.1016, found: 147.1018; $[M + Na]^+$ calcd for $C_7H_{14}NaO_3$: 169.0835, found: 169.0837.

(3*S*,4*R*)-4-(Methoxymethoxy)pent-1-en-3-ol (*ent*-14a). Prepared as described for **14a** from *ent*-**13** (2.49 g, 9.59 mmol) and TBAF.$3H_2O$ (6.94 g, 18.22 mmol, 1.9 equiv) in THF (95 mL). *ent*-**14a** (1.22 g, 87% yield, colorless oil); TLC R_f 0.20 (Hex/Et_2O: 50/50); $[\alpha]_D^{25}$ -58.0 (*c* 1.45, $CHCl_3$); IR (ATR) ν_{max} 3447, 2934, 1378, 1148, 1101, 1027, 917 cm^{-1}; ^1H NMR (300 MHz, $CDCl_3$) δ_H 5.88 (ddd, *J* = 17.3, 10.5, 5.9 Hz, 1 H, H-4), 5.32 (dt, *J* = 17.3, 1.6 Hz, 1 H), 5.24 (dt, *J* = 10.5, 1.6 Hz, 1 H), 4.71 (d, *J* = 6.9 Hz, 1 H), 4.69 (d, *J* = 6.9 Hz, 1 H),4.15 – 4.08 (m, 1 H), 3.78 (qd, *J* = 6.5, 3.0 Hz, 1 H), 3.40 (s, 3 H), 2.78 (d, *J* = 6.0 Hz, 1 H), 1.16 (d, *J* = 6.5 Hz, 3 H) ppm; ^{13}C NMR (75 MHz, $CDCl_3$) δ_C 136.4, 116.9, 96.2, 78.1, 75.3, 55.8, 15.7 ppm; HRMS (ESI) *m/z* $[M + Na]^+$ calcd for $C_7H_{14}NaO_3$: 169.0835, found: 169.0836.

(3*S*,4*S*)-4-(Methoxymethoxy)pent-1-en-3-yl 4-nitrobenzoate (15). To a solution of PPh_3 (592 mg, 2.26 mmol, 1.1 equiv) and 4-nitrobenzoic acid (377 mg, 2.26 mmol, 1.1 equiv) in THF (8.2 mL) at 0 °C was added DEAD (0.95 mL, 375 mg, 174.15 mmol, 1.05 equiv, 40 % in

toluene) and solution of alcohol **14a** (300 mg, 2.05 mmol, 1 equiv) in THF (1 mL). Reaction mixture was left to stir for 1.5 h to full conversion (monitored by TLC). Reaction mixture was concentrated under reduced pressure and adsorbed on silica gel. The residue was purified using MPLC (5 min. gradient from Hex/EtOAc: 100/0 to Hex/EtOAc: 95/5) providing desired product **15** (375 mg, 62% yield, yellow oil); TLC R_f 0.12 (Hex/EtOAc: 90/10); $[\alpha]_D^{25}$ -47.2 (*c* 1.54, $CHCl_3$); IR (ATR) ν_{max} 2936, 1724, 1525, 1265, 1100, 1030, 917 cm^{-1}; ^1H NMR (300 MHz, $CDCl_3$) δ_H 8.32 – 8.22 (m, 4 H_{Ar}), 5.94 (ddd, *J* = 17.2, 10.6, 6.6 Hz, 1 H), 5.55 (ddt, *J* = 7.2, 6.0, 1.2 Hz, 1 H), 5.43 (dt, *J* = 17.2, 1.3 Hz, 1 H), 5.35 (dt, *J* = 10.6, 1.2 Hz, 1 H), 4.71 (d, *J* = 6.9 Hz, 1 H), 4.68 (d, *J* = 6.9 Hz, 1 H), 3.97 (quint, *J* = 6.4 Hz, 1 H), 3.35 (s, 3 H), 1.25 (d, *J* = 6.4 Hz, 3 H) ppm; ^{13}C NMR (75 MHz, $CDCl_3$) δ_C 163.9, 150.8, 135.8, 132.6, 130.9, 123.7, 119.6, 95.8, 78.7, 73.9, 55.8, 16.7 ppm; g (ESI) *m/z* $[M + Na]^+$ calcd for: $C_{14}H_{17}NNaO_6$: 318.0948, found: 318.0950; $[M + K]^+$ calcd for $C_{12}H_{12}KO_4$: 334.0687, found: 334.0688.

(3*R*,4*R*)-4-(Methoxymethoxy)pent-1-en-3-yl 4-nitrobenzoate (*ent*-15). Prepared as described for **15** from *ent*-**14a** (150 mg, 1.03 mmol, 1 equiv), PPh_3 (296 mg, 1.13 mmol, 1.1 equiv), 4-nitrobenzoic acid (188 mg, 1.13 mmol, 1.1 equiv) and DEAD (0.48 mL, 187 mg, 1.08 mmol, 1.05 equiv, 40 % in toluene) in THF (6 mL). Compound *ent*-**15** (206 mg, 68% yield, yellow oil); TLC R_f 0.11 (Hex/EtOAc: 95/5); $[\alpha]_D^{25}$ +44.6 (*c* 1.04, $CHCl_3$); IR (ATR) ν_{max} 2936, 1724, 1525, 1265, 1030, 917, 717 cm^{-1}; ^1H NMR (300 MHz, $CDCl_3$) δ_H 8.33 – 8.21 (m, 4 H), 5.94 (ddd, *J* = 17.2, 10.6, 6.6 Hz, 1 H), 5.55 (ddt, *J* = 7.2, 6.1, 1.2 Hz, 1 H), 5.43 (dt, *J* = 17.2, 1.3 Hz, 1 H), 5.35 (dt, *J* = 10.6, 1.2 Hz, 1 H), 4.71 (d, *J* = 6.9 Hz, 1 H), 4.68 (d, *J* = 6.9 Hz, 1 H), 3.97 (quint, *J* = 6.4 Hz, 1 H), 3.35 (s, 3 H), 1.25 (d, *J* = 6.4 Hz, 3 H) ppm; ^{13}C NMR (75 MHz, $CDCl_3$) δ_C 163.9, 150.8, 135.8, 132.6, 130.9, 123.7, 119.6, 95.8, 78.7, 73.9, 55.8, 16.6 ppm; HRMS (ESI) *m/z* $[M + Na]^+$ calcd for $C_{14}H_{17}NNaO_6$: 318.0948, found: 318.0953.

(3*S*,4*S*)-4-(Methoxymethoxy)pent-1-en-3-ol (14b). To a solution of benzoate **15** (278 mg, 0.94 mmol) in MeOH (4.2 mL) was added dropwise solution of K_2CO_3 (142.64 mg, 1.03 mmol, 1.1 equiv) in water (0.4 mL). Reaction mixture was left to stir at ambient temperature for 3 h

to full conversion (monitored by TLC). Reaction mixture was concentrated under reduced pressure (min. 200 mbar). The residue was then dissolved in DCM (5.6 mL), dried over anhydrous Na_2SO_4, filtered, and concentrated under reduced pressure (min. 200 mbar). The residue was adsorbed on silica gel and purified using MPLC (5 min. gradient from Hex/Et_2O: 100/0 to Hex/Et_2O: 80/20) providing desired product **14b** (120 mg, 87% yield, yellow oil); TLC R_f 0.30 (Hex/Et_2O: 70/30); $[\alpha]_D^{25}$ +39.2 (*c* 0.36, $CHCl_3$); IR (ATR) ν_{max} 3446, 2934, 1449, 1378, 1133, 1027, 917 cm^{-1}; ^1H NMR (300 MHz, $CDCl_3$) δ_H 5.83 (ddd, *J* = 17.2, 10.4, 6.3 Hz, 1 H), 5.37 (ddd, *J* = 17.2, 1.6, 1.3 Hz, 1 H), 5.23 (ddd, *J* = 10.4, 1.6, 1.3 Hz, 1 H), 4.74 (d, *J* = 6.8 Hz, 1 H), 4.69 (d, *J* = 6.8 Hz, 1 H), 3.97 – 3.89 (m, 1 H), 3.57 (quint, *J* = 6.4 Hz, 1 H), 3.41 (s, 3 H), 2.91 (d, *J* = 3.2 Hz, 1 H), 1.18 (d, *J* = 6.3 Hz, 3 H) ppm; ^{13}C NMR (75 MHz, $CDCl_3$) δ_C 137.1, 117.5, 96.2, 78.2, 76.7, 55.8, 17.0 ppm; HRMS (ESI) *m/z* $[M + Na]^+$ calcd for $C_7H_{14}NaO_3$: 169.0835, found: 169.0837; $[M + K]^+$ calcd for $C_7H_{14}KO_3$: 185.0575, found: 185.0575.

(3*R*,4*R*)-4-(Methoxymethoxy)pent-1-en-3-ol (*ent*-14b). Prepared as above from *ent*-**15** (820 mg, 2.77 mmol), solution of K_2CO_3 (420.73 mg, 3.04 mmol, 1.1 equiv) in water (1.3 mL). Compound *ent*-**14b** (380 mg, 94% yield, yellow oil); TLC R_f 0.20 (Hex/Et_2O: 70/30); $[\alpha]_D^{25}$ -24.2 (*c* 0.70, $CHCl_3$); IR (ATR) ν_{max} 3446, 2934, 1378, 1133, 1027, 993, 917 cm^{-1}; ^1H NMR (300 MHz, $CDCl_3$) δ_H 5.83 (ddd, *J* = 17.2, 10.4, 6.3 Hz, 1 H), 5.37 (ddd, *J* = 17.2, 1.6, 1.3 Hz, 1 H), 5.23 (ddd, *J* = 10.4, 1.6, 1.3 Hz, 1 H), 4.75 (d, *J* = 6.8 Hz, 1 H), 4.69 (d, *J* = 6.8 Hz, 1 H), 3.97 – 3.89 (m, 1 H), 3.57 (quint, *J* = 6.4 Hz, 1 H), 3.41 (s, 3 H), 2.91 (d, *J* = 3.2 Hz, 1 H), 1.18 (d, *J* = 6.3 Hz, 3 H) ppm; ^{13}C NMR (151 MHz, $CDCl_3$) δ_C 137.1, 117.5, 96.2, 78.2, 76.7, 55.8, 17.0 ppm; HRMS (ESI) *m/z* $[M + Na]^+$ calcd for $C_7H_{14}NaO_3$: 169.0835, found: 169.0836.

(3*R*,4*S*)-4-(Methoxymethoxy)pent-1-en-3-yl 2-hydroxy-6-vinylbenzoate (16a). NaH (1.64 g, 6.08 mmol, 3.2 equiv, 60 wt-% in mineral oil) was washed by hexane. The suspension of NaH in THF (13.5 mL) was cooled to 0 °C and alcohol **14a** (361 mg, 2.47 mmol, 1.3 equiv) in THF (2.7 mL) was added dropwise. Styrene **8** (388 mg,

1.9 mmol) in THF (4.8 mL) was added dropwise after 5 min of stirring. Reaction mixture was left to stir continuously at an ambient temperature for 2 h to full conversion (monitored by TLC). After 2 h of stirring, the reaction was quenched by saturated NH_4Cl solution (10 mL). Aqueous layer was extracted by EtOAc (3 x 50 mL). Combined organic layers were dried over anhydrous Na_2SO_4, filtered, and concentrated under reduced pressure. The residue was adsorbed on silica gel and purified using MPLC (3 min. gradient from Hex/EtOAc: 100/0 to Hex/EtOAc: 92/8) providing desired product **16a** (445 mg, 80% yield, yellow oil); TLC R_f 0.13 (Hex : EtOAc = 95 : 5); $[\alpha]_D^{25}$ +35.4 (*c* 1.2, $CHCl_3$); IR (ATR) ν_{max} 2939, 1732, 1660, 1448, 1212, 1029, 916 cm^{-1}; ^1H NMR (300 MHz, $CDCl_3$) δ_H 10.93 (d, *J* = 0.4 Hz, 1 H, OH), 7.41 – 7.30 (m, 2 H), 6.97 (ddd, *J* = 7.6, 1.2, 0.7 Hz, 1 H), 6.93 (dd, *J* = 8.2, 1.1 Hz, 1 H), 5.91 (ddd, *J* = 17.2, 10.7, 6.5 Hz, 1 H), 5.67 (ddt, *J* = 6.5, 3.4, 1.2 Hz, 1 H), 5.50 (dd, *J* = 17.3, 1.5 Hz, 1 H), 5.39 (dt, *J* = 17.3, 1.2 Hz, 1 H), 5.35 (dt, *J* = 10.6, 1.2 Hz, 1 H), 5.26 (dd, *J* = 10.8, 1.5 Hz, 1 H), 4.72 (d, *J* = 7.0 Hz, 1 H), 4.69 (d, *J* = 7.0 Hz, 1 H), 3.97 (qd, *J* = 6.5, 3.4 Hz, 1 H) 3.36 (s, 3 H), 1.29 (d, *J* = 6.5 Hz, 3 H) ppm; ^{13}C NMR (151 MHz, $CDCl_3$) δ_C 170.2, 162.1, 142.0, 138.5, 134.6, 132.4, 120.0, 119.5, 117.3, 116.0, 111.4, 95.6, 78.8, 73.9, 55.7, 16.1 ppm; HRMS (ESI) *m/z* $[M + Na]^+$ calcd for $C_{16}H_{20}NaO_5$: 315.1203, found: 315.1204; $[M + K]^+$ calcd for $C_{16}H_{20}KO_5$: 331.0942, found: 331.0944.

(3*S*,4*R*)-4-(Methoxymethoxy)pent-1-en-3-yl 2-hydroxy-6-vinylbenzoate (*ent*-16a). Prepared as **16a** from *ent*-**14a** (139 mg, 0.95 mmol, 1.3 equiv), NaH (631 mg, 2.34 mmol, 3.2 equiv) and **4** (149 mg, 0.73 mmol) in THF. Compound *ent*-**16a** (168 mg, 79% yield, yellow oil); R_f 0.13 (Hex/EtOAc: 95/5); $[\alpha]_D^{25}$ -39.8 (*c* 1.45, $CHCl_3$); IR (ATR) ν_{max} 2938, 1732, 1661, 1448, 1250, 1029, 818 cm^{-1}; ^1H NMR (300 MHz, $CDCl_3$) δ_H 10.93 (s, 1 H, OH), 7.41 – 7.29 (m, 2 H), 6.97 (ddd, *J* = 7.6, 1.1, 0.6 Hz, 1 H), 6.93 (dd,

J = 8.3, 1.1 Hz, 1 H), 5.91 (ddd, *J* = 17.2, 10.6, 6.5 Hz, 1 H), 5.70 – 5.64 (m, 1 H), 5.50 (dd, *J* = 17.2, 1.5 Hz, 1 H), 5.39 (dt, *J* = 17.3, 1.2 Hz, 1 H), 5.35 (dt, *J* = 10.6, 1.2 Hz, 1 H), 5.26 (dd, *J* = 10.9, 1.5 Hz, 1 H), 4.72 (d, *J* = 7.0 Hz, 1 H), 4.69 (d, *J* = 7.0 Hz, 1 H), 3.97 (qd, *J* = 6.5, 3.4 Hz, 1 H) 3.36 (s, 3 H), 1.29 (d, *J* = 6.5 Hz, 3 H) ppm; ^{13}C NMR (75 MHz, $CDCl_3$) δ_C 170.2, 162.1, 142.0, 138.5, 134.6, 132.4, 120.0, 119.5, 117.3, 116.0, 111.4, 95.6, 78.8, 73.9, 55.7, 16.1 ppm; HRMS (ESI) *m/z* $[M + H]^+$ calcd for $C_{16}H_{21}O_5$: 293.1384, found: 293.1386; $[M + Na]^+$ calcd for $C_{16}H_{20}NaO_5$: 315.1203, found: 315.1204; $[M + K]^+$ calcd for $C_{16}H_{20}KO_5$: 331.0942, found: 331.0945.

(3*S*,4*S*)-4-(Methoxymethoxy)pent-1-en-3-yl 2-hydroxy-6-vinylbenzoate (16b). Prepared as **16a** from **14b** (88 mg, 0.60 mmol, 1.3 equiv), NaH (402 mg, 1.49 mmol, 3.2 equiv) and **8** (95 mg, 0.47 mmol) in THF. Compound **16b** (115 mg, 85% yield, yellow oil); TLC R_f 0.25 (Hex/EtOAc: 90/10); $[\alpha]_D^{25}$ -72.6 (*c* 0.45, $CHCl_3$); IR (ATR) ν_{max} 2935, 1661, 1148, 1249, 1212, 1030, 917 cm^{-1}; ^1H NMR (300 MHz, $CDCl_3$) δ_H 11.05 (d, *J* = 0.3 Hz, 1 H), 7.41 – 7.29 (m, 2 H), 6.98 – 6.91 (m, 2 H), 5.94 (ddd, *J* = 17.2, 10.6, 6.5 Hz, 1 H), 5.60 (ddt, *J* = 7.0, 5.9, 1.2 Hz, 1 H), 5.47 (dd, *J* = 17.2, 1.6 Hz, 1 H), 5.43 (dt, *J* = 17.2, 1.2 Hz, 1 H), 5.35 (dt, *J* = 10.6, 1.2 Hz, 1 H), 5.25 (dd, *J* = 10.9, 1.6 Hz, 1 H), 4.74 – 4.66 (m, 2 H), 3.94 (quint, *J* = 6.4 Hz, 1 H), 3.36 (s, 3 H), 1.26 (d, *J* = 6.4 Hz, 3 H) ppm; ^{13}C NMR (75 MHz, $CDCl_3$) δ_C 170.1, 162.3, 142.1, 138.6, 134.6, 132.4, 120.1, 119.7, 117.4, 116.0, 111.0, 95.9, 78.7, 74.2, 55.8, 16.7 ppm; HRMS (ESI) *m/z* $[M + H]^+$ calcd for $C_{16}H_{21}O_5$: 293.1384, found: 293.1387; $[M + Na]^+$ calcd for $C_{16}H_{20}NaO_5$: 315.1203, found: 315.1206; $[M + K]^+$ calcd for $C_{16}H_{20}KO_5$: 331.0942, found: 331.0945.

(3*R*,4*R*)-4-(Methoxymethoxy)pent-1-en-3-yl 2-hydroxy-6-vinylbenzoate (*ent*-16b). Prepared from *ent*-**14b** (205 mg, 1.40 mmol, 1.3 equiv), NaH (932 mg, 3.45

mmol, 3.2 equiv) and **8** (220 mg, 1.08 mmol) in THF. Compound *ent*-**16b** (225 mg, 71% yield, yellow oil); TLC R_f 0.28 (Hex/EtOAc: 90/10); $[\alpha]_D^{25}$ +86.4 (*c* 0.34, $CHCl_3$); IR (ATR) ν_{max} 2935, 1731, 1661, 1448, 1249, 1030, 817 cm^{-1}; ^1H NMR (300 MHz, $CDCl_3$) δ_H 11.05 (d, *J* = 0.3 Hz, 1 H), 7.42 – 7.29 (m, 2 H), 6.99 – 6.91 (m, 2 H), 5.94 (ddd, *J* = 17.2, 10.6, 6.5 Hz, 1 H), 5.60 (ddt, *J* = 7.0, 5.9, 1.2 Hz, 1 H), 5.47 (dd, *J* = 17.2, 1.6 Hz, 1 H), 5.43 (dt, *J* = 17.2, 1.2 Hz, 1 H), 5.35 (dt, *J* = 10.6, 1.2 Hz, 1 H), 5.25 (dd, *J* = 10.9, 1.6 Hz, 1 H), 4.74 – 4.66 (m, 2 H), 3.94 (quint, *J* = 6.3 Hz, 1 H), 3.36 (s, 3 H), 1.26 (d, *J* = 6.4 Hz, 3 H) ppm; ^{13}C NMR (75 MHz, $CDCl_3$) δ_C 170.1, 162.3, 142.1, 138.6, 134.6, 132.4, 120.1, 119.7, 117.4, 116.0, 111.0, 95.9, 78.7, 74.2, 55.8, 16.7 ppm; HRMS (ESI) *m/z* $[M + Na]^+$ calcd for $C_{16}H_{20}NaO_5$: 315.1203, found: 315.1204; $[M + K]^+$ calcd for $C_{16}H_{20}KO_5$: 331.0942, found: 331.0945.

(*R*)-9-Hydroxy-3-((*S*)-1-(methoxymethoxy)ethyl)benzo[*c*]oxepin-1(3H)-one (17a). To a solution of ester **16a** (100 mg, 0.34 mmol) in toluene (34 mL) was added Grubbs catalyst 2nd generation (36 mg, 0.04 mmol, 0.13 equiv). Reaction mixture was left to stir at reflux for 24 h. Reaction mixture was adsorbed on silica gel and then purified using MPLC (5 min. gradient from Hex/EtOAc: 100/0 to Hex/EtOAc: 75/15) providing desired product **17a** (66 mg, 73% yield, brown oil); TLC R_f 0.23 (Hex : EtOAc = 80 : 20); $[\alpha]_D^{25}$ +354.2 (*c* 0.87, $CHCl_3$); IR (ATR) ν_{max} 2940, 2825, 1661, 1443, 1238, 1005, 819 cm^{-1}; ^1H NMR (300 MHz, $CDCl_3$) δ_H 10.47 (d, *J* = 0.4 Hz, 1 H), 7.43 (t, *J* = 8.0 Hz, 1 H), 7.02 (dd, *J* = 8.4, 1.2 Hz, 1 H), 6.89 (dd, *J* = 10.4, 1.8 Hz, 1 H), 6.80 (ddd, *J* = 7.6, 1.1, 0.6 Hz, 1H), 6.35 (dd, *J* = 10.4, 5.8 Hz, 1 H), 4.77 (s, 2 H), 4.57 (ddd, *J* = 5.8, 4.2, 1.8 Hz, 1 H), 4.10 (qd, *J* = 6.5, 4.2 Hz, 1 H), 3.43 (s, 3 H), 1.31 (d, *J* = 6.5 Hz, 3 H) ppm; ^{13}C NMR (75 MHz, $CDCl_3$) δ_C 172.3, 162.1, 136.9, 136.3, 134.2, 129.3, 121.3, 118.2, 114.0, 95.8, 77.0, 72.9, 55.8, 16.6 ppm; HRMS (ESI) *m/z* $[M + H]^+$ calcd for

$C_{14}H_{17}O_5$: 265.1071, found: 265.1072; [M + Na]$^+$ calcd for $C_{14}H_{16}NaO_5$: 287.0890, found: 287.0891; [M + K]$^+$ calcd for $C_{14}H_{16}KO_5$: 303.0629, found: 303.0631.

(*S*)-9-Hydroxy-3-((*R*)-1-(methoxymethoxy)ethyl)benzo[*c*]oxepin-1(3H)-one(*ent*-17a). Prepared as described for **17a** from ester *ent*-**16a** (252 mg, 0.86 mmol) and Grubbs catalyst 2nd generation (95 mg, 0.11 mmol, 0.13 equiv) in toluene (86 mL). Compound *ent*-**17a** (187 mg, 82% yield, brown oil); TLC R_f 0.23 (Hex/EtOAc: 80/20); $[\alpha]_D^{25}$ -395.5 (*c* 1.30, $CHCl_3$); IR (ATR) ν_{max} 2940, 1661, 1443, 1239, 1114, 1005, 819 cm^{-1}; ^{1}H NMR (300 MHz, $CDCl_3$) δ_H 10.47 (s, 1 H, OH), 7.43 (t, *J* = 8.0 Hz, 1 H), 7.02 (dd, *J* = 8.4, 1.0 Hz, 1 H), 6.88 (dd, *J* = 10.4, 1.7 Hz, 1 H), 6.80 (d, *J* = 7.5, 1 H), 6.35 (dd, *J* = 10.4, 5.8 Hz, 1 H), 4.77 (s, 2 H), 4.57 (ddd, *J* = 5.8, 4.2, 1.7 Hz, 1 H), 4.10 (qd, *J* = 6.5, 4.2 Hz, 1 H), 3.43 (s, 3 H), 1.31 (d, *J* = 6.5 Hz, 3 H) ppm; ^{13}C NMR (75 MHz, $CDCl_3$) δ_C 172.3, 162.1, 136.9, 136.3, 134.2, 129.2, 121.3, 118.2, 114.0, 95.8, 77.0, 72.9, 55.8, 16.6 ppm; HRMS (ESI) *m/z* [M + H]$^+$ calcd for $C_{14}H_{17}O_5$: 265.1071, found: 265.1072; [M + Na]$^+$ calcd for $C_{14}H_{16}NaO_5$: 287.0890, found: 287.0891; [M + K]$^+$ calcd for $C_{14}H_{16}KO_5$: 303.0629, found: 303.0631.

(*S*)-9-Hydroxy-3-((*S*)-1-(methoxymethoxy)ethyl)benzo[*c*]oxepin-1(3H)-one (17b). Prepared as described for **17a** from ester **16b** (19 mg, 0.065 mmol) and Grubbs catalyst 2nd generation (7 mg, 0.008 mmol, 0.13 equiv) in toluene (6.5 mL). Compound **17b** (12 mg, 71% yield, brown oil); TLC R_f 0.18 (Hex/EtOAc: 80/20); $[\alpha]_D^{25}$ -349.6 (*c* 0.22, $CHCl_3$); IR (ATR) ν_{max} 2935, 1659, 1443, 1236, 1022, 821, 696 cm^{-1}; ^{1}H NMR (300 MHz, $CDCl_3$) δ_H 10.51 (d, J = 0.4 Hz, 1 H), 7.43 (t, *J* = 8.0 Hz, 1 H), 7.02 (dd, *J* = 8.3, 1.2 Hz, 1 H), 6.88 (dd, *J* = 10.4, 1.7 Hz, 1 H), 6.80 (d, *J* = 7.5 Hz, 1 H), 6.27 (dd, *J* = 10.4, 5.8 Hz, 1 H), 4.76 (d, *J* = 6.9 Hz, 1 H), 4.71 (d, *J* = 6.9 Hz, 1 H), 4.66 (td, *J* = 6.0, 1.8 Hz, 1 H), 4.09 (quint, *J* = 6.3 Hz, 1 H), 3.39 (s, 3 H), 1.34 (d, *J* = 6.4 Hz, 3 H)

ppm; ^{13}C NMR (75 MHz, $CDCl_3$) δ_C 172.3, 162.2, 136.9, 136.3, 134.2, 129.2, 121.3, 118.3, 114.1, 96.0, 76.2, 72.6, 55.9, 16.0 ppm; HRMS (ESI) *m/z* $[M + H]^+$ calcd for $C_{14}H_{17}O_5$: 265.1071, found: 265.1071; $[M + Na]^+$ calcd for $C_{14}H_{16}NaO_5$: 287.0890, found: 287.0891; $[M + K]^+$ calcd for $C_{14}H_{16}KO_5$: 303.0629, found: 303.0631.

(*R*)-9-Hydroxy-3-((*R*)-1-(methoxymethoxy)ethyl)benzo[*c*]oxepin-1(3H)-one(*ent*-17b). Prepared as described for **17a** from ester *ent*-**16b** (71 mg, 0.24 mmol) and Grubbs catalyst 2nd generation (31 mg, 0.03 mmol, 0.15 equiv) in toluene (24 mL). Compound *ent*-**17b** (54 mg, 84% yield, brown oil); TLC R_f 0.19 (Hex/EtOAc: 80/20); $[\alpha]_D^{25}$ +337.2 (*c* 0.45, $CHCl_3$); IR (ATR) ν_{max} 2935, 2823, 1660, 1443, 1237, 1027, 822 cm^{-1}; ^1H NMR (300 MHz, $CDCl_3$) δ_H 10.51 (d, J = 0.4 Hz, 1 H), 7.43 (t, *J* = 8.0 Hz, 1 H), 7.02 (dd, *J* = 8.4, 1.2 Hz, 1 H), 6.88 (dd, *J* = 10.5, 1.7 Hz, 1 H), 6.80 (dd, *J* = 7.3, 0.9 Hz, 1 H), 6.27 (dd, *J* = 10.4, 5.8 Hz, 1 H), 4.74 (d, *J* = 6.9 Hz, 1 H), 4.71 (d, *J* = 6.9 Hz, 1 H), 4.66 (td, *J* = 6.0, 1.8 Hz, 1 H), 4.09 (quint, *J* = 6.3 Hz, 1 H), 3.39 (s, 3 H), 1.34 (d, *J* = 6.4 Hz, 3 H) ppm; ^{13}C NMR (75 MHz, $CDCl_3$) δ_C 172.4, 162.2, 136.9, 136.3, 134.2, 129.2, 121.3, 118.3, 114.1, 96.0, 76.2, 72.6, 55.9, 16.0 ppm; HRMS (ESI) *m/z* $[M + H]^+$ calcd for $C_{14}H_{17}O_5$: 265.1071, found: 265.1072; $[M + Na]^+$ calcd for $C_{14}H_{16}NaO_5$: 287.0890, found: 287.0892; $[M + K]^+$ calcd for $C_{14}H_{16}KO_5$: 303.0629, found: 303.0631.

(*R*)-9-Hydroxy-3-((*S*)-1-hydroxyethyl)benzo[*c*]oxepin-1(3H)-one (3a). To a solution of ester **17a** (215 mg, 0.81 mmol) in ^{i}PrOH (8 mL) Dowex Marathon (480 mg) was added. Reaction mixture was left to stir at room temperature. After 4h of stirring, another portion of Dowex Marathon (480 mg) was added to the reaction mixture. After 24 h of stirring, Dowex Marathon was filtered and washed with methanol. After concentration, the residue was adsorbed on silica gel and then purified using MPLC (5 min. gradient from Hex/EtOAc: 100/0 to Hex/EtOAc: 75/25) providing desired product **3a** (160 mg, 89% yield, colorless oil, white

solid after cooling to -3 °C); TLC R_f 0.10 (Hex/EtOAc: 80/20); $[\alpha]_D^{25}$ +479.9 (*c* 0.39, MeOH); IR (ATR) ν_{max} 3369, 2976, 1655, 1442, 1236, 1110, 820 cm^{-1}; ^{1}H NMR (600 MHz, $CDCl_3$) δ_H 10.42 (d, *J* = 0.2 Hz, 1 H), 7.44 (t, *J* = 7.9 Hz, 1 H), 7.02 (dd, *J* = 8.3, 1.1 Hz, 1 H), 6.91 (dd, *J* = 10.4, 1.7 Hz, 1 H), 6.81 (d, *J* = 7.3 Hz, 1 H), 6.39 (dd, *J* = 10.4, 5.7 Hz, 1 H), 4.56 (ddd, *J* = 5.7, 3.2, 1.8 Hz, 1 H), 4.26 – 4.21 (m, 1 H), 2.27 (s, 1 H), 1.28 (d, *J* = 6.6 Hz, 3 H) ppm; ^{1}H NMR (600 MHz, CD_3OD) δ_H 7.45 (t, *J* = 7.9 Hz, 1 H), 6.98 (brd, *J* = 10.4 Hz, 1 H), 6.98 – 6.96 (m, 1 H), 6.88 (d, *J* = 7.6 Hz, 1 H), 6.41 (dd, *J* = 10.4, 5.8 Hz, 1 H), 4.46 (ddd, *J* = 5.8, 4.7, 1.8 Hz, 1 H), 4.10 – 4.05 (m, 1 H), 1.27 (d, *J* = 6.5 Hz, 3 H) ppm; ^{13}C NMR (151 MHz, $CDCl_3$) δ_C 172.4, 162.1, 137.0, 136.7, 134.3, 128.2, 121.4, 118.3, 113.9, 78.1, 67.8, 17.9 ppm; ^{13}C NMR (151 MHz, CD_3OD) δ_C 172.5, 161.8, 138.4, 136.8, 134.7, 131.2, 121.8, 118.3, 116.5, 79.0, 68.3, 18.8 ppm; HRMS (ESI) *m/z* [M + H]$^+$ calcd for $C_{12}H_{13}O_4$: 221.0808, found: 221.0809; [M + Na]$^+$ calcd for $C_{12}H_{12}NaO_4$: 243.0628, found: 243.0629; [M + K]$^+$ calcd for $C_{12}H_{12}KO_4$: 259.0367, found: 259.0368.

(*S*)-9-Hydroxy-3-((*R*)-1-hydroxyethyl)benzo[*c*]oxepin-1(3H)-one (*ent*-3a). Prepared as described for **3a** from *ent*-**17a** (116 mg, 0.44 mmol) and Dowex Marathon (250 mg) in ^{i}PrOH (4.4 mL). Compound *ent*-**3a** (79 mg, 82% yield, colorless oil, white solid after cooling to -3 °C); TLC R_f 0.10 (Hex/EtOAc: 80/20); $[\alpha]_D^{25}$ -463.7 (*c* 0.35, MeOH); IR (ATR) ν_{max} 3367, 2976, 1655, 1599, 1442, 1235, 820 cm^{-1}; ^{1}H NMR (600 MHz, $CDCl_3$) δ_H 10.41 (s, 1 H), 7.43 (t, *J* = 7.9 Hz, 1 H), 7.01 (dd, *J* = 8.3, 1.0 Hz, 1 H), 6.89 (dd, *J* = 10.4, 1.5 Hz, 1 H), 6.80 (d, *J* = 7.5 Hz, 1 H), 6.37 (dd, *J* = 10.4, 5.7 Hz, 1 H), 4.55 (ddd, *J* = 5.7, 3.3, 1.9 Hz, 1 H), 4.26 – 4.18 (m, 1 H), 2.44 (s, 1 H), 1.28 (d, *J* = 6.6 Hz, 3 H) ppm; ^{1}H NMR (600 MHz, CD_3OD) δ_H 7.45 (t, *J* = 7.9 Hz, 1 H), 6.98 (brd, *J* = 10.4 Hz, 1 H), 6.98 – 6.96 (m, 1 H), 6.88 (d, *J* = 7.6 Hz, 1 H), 6.40 (dd, *J* = 10.4, 5.8 Hz, 1 H), 4.45 (ddd, *J* = 5.8, 4.7, 1.7 Hz, 1 H), 4.12 – 4.02 (m, 1 H), 1.27 (d, *J* = 6.5 Hz, 3 H) ppm; ^{13}C NMR (151 MHz, $CDCl_3$) δ_C 172.4, 162.1, 137.0, 136.6, 134.3, 128.3, 121.4, 118.2, 113.8, 78.1, 67.7, 17.9 ppm; ^{13}C NMR (151 MHz, CD_3OD) δ_C

172.5, 161.8, 138.4, 136.8, 134.7, 131.2, 121.8, 118.3, 116.4, 79.0, 68.3, 18.8 ppm; HRMS (ESI) *m/z* $[M + H]^+$ calcd for $C_{12}H_{13}O_4$: 221.0808, found: 221.0809; $[M + Na]^+$ calcd for $C_{12}H_{12}NaO_4$: 243.0628, found: 243.0628; $[M + K]^+$ calcd for $C_{12}H_{12}KO_4$: 259.0367, found: 259.0368.

(*S*)-9-Hydroxy-3-((S)-1-hydroxyethyl)benzo[*c*]oxepin-1(3H)-one (3b). Prepared as described for **3a** from **17b** (50 mg, 0.19 mmol) and Dowex Marathon (110 mg) in ^{i}PrOH (1.9 mL). Compound **3b** (35 mg, 84% yield, colorless oil, white solid after cooling to -3 °C); TLC R_f 0.20 (Hex/EtOAc: 70/30); $[\alpha]_D^{25}$ -391.9 (*c* 0.65, MeOH); IR (ATR) ν_{max} 3368, 1655, 1599, 1442, 1235, 1109, 1005 cm^{-1}; ^1H NMR (600 MHz, $CDCl_3$) δ_H 10.42 (s, 1 H), 7.44 (dd, *J* = 8.2, 7.7 Hz, 1 H), 7.03 (dd, *J* = 8.3, 1.0 Hz, 1 H), 6.87 (dd, *J* = 10.3, 1.5 Hz, 1 H), 6.81 (d, *J* = 7.5 Hz, 1 H), 6.18 (dd, *J* = 10.3, 5.8 Hz, 1 H), 4.44 (ddd, *J* = 7.3, 5.9, 1.7 Hz, 1 H), 4.14 – 4.09 (m, 1 H), 2.64 (s, 1 H), 1.30 (d, *J* = 6.4 Hz, 3 H) ppm; ^1H NMR (600 MHz, CD_3OD) δ_H 7.45 (t, *J* = 7.9 Hz, 1 H), 6.97 (dd, *J* = 8.2, 1.1 Hz, 1 H), 6.95 (brd, *J* = 10.4 Hz, 1 H), 6.88 (d, *J* = 7.5 Hz, 1 H), 6.34 (dd, *J* = 10.3, 5.9 Hz, 1 H), 4.46 (td, *J* = 5.8, 1.7 Hz, 1 H), 4.06 (quint, *J* = 6.4 Hz, 1 H), 1.28 (d, *J* = 6.5 Hz, 3 H) ppm; ^{13}C NMR (151 MHz, $CDCl_3$) δ_C 172.3, 162.2, 136.9, 136.5, 134.4, 129.3, 121.4, 118.4, 113.9, 78.7, 67.9, 18.3 ppm; ^{13}C NMR (151 MHz, CD_3OD) δ_C 172.5, 161.8, 138.3, 136.8, 134.7, 131.4, 121.8, 118.4, 116.5, 79.1, 68.1, 18.6 ppm; HRMS (ESI) *m/z* $[M + H]^+$ calcd for $C_{12}H_{13}O_4$: 221.0808, found: 221.0809; $[M + Na]^+$ calcd for $C_{12}H_{12}NaO_4$: 243.0628, found: 243.0629; $[M + K]^+$ calcd for $C_{12}H_{12}KO_4$: 259.0367, found: 259.0368.

(*R*)-9-Hydroxy-3-((*R*)-1-hydroxyethyl)benzo[*c*]oxepin-1(3H)-one (*ent*-3b). Prepared as described for **3a** from *ent*-**17b** (33 mg, 0.12 mmol) and Dowex Marathon (75 mg) in ^{i}PrOH (1.2 mL). Compound *ent*-**3b** (22 mg, 80% yield, colorless oil); TLC

R_f 0.19 (Hex/EtOAc: 80/20); $[\alpha]_D^{25}$ +394.7 (*c* 0.40, MeOH); IR (ATR) ν_{max} 3366, 2977, 1655, 1442, 1235, 1005, 821 cm^{-1}; ^1H NMR (300 MHz, $CDCl_3$) δ_H 10.42 (s, 1 H), 7.44 (t, *J* = 8.0 Hz, 1 H), 7.04 (dd, *J* = 8.3, 1.0 Hz, 1 H), 6.88 (dd, *J* = 10.4, 1.5 Hz, 1 H), 6.81 (d, *J* = 7.3 Hz, 1 H), 6.18 (dd, *J* = 10.3, 5.8 Hz, 1 H), 4.44 (ddd, *J* = 7.2, 5.9, 1.7 Hz, 1 H), 4.18 – 4.06 (m, 1 H), 2.52 (s, 1 H), 1.30 (d, *J* = 6.4 Hz, 3 H) ppm; ^1H NMR (600 MHz, CD_3OD) δ_H 7.45 (t, *J* = 7.9 Hz, 1 H), 6.97 (dd, *J* = 8.2, 1.1 Hz, 1 H), 6.95 (brd, *J* = 10.4 Hz, 1 H), 6.87 (d, *J* = 7.5 Hz, 1 H), 6.34 (dd, *J* = 10.3, 5.9 Hz, 1 H), 4.46 (td, *J* = 5.8, 1.7 Hz, 1 H), 4.06 (quint, *J* = 6.4 Hz, 1 H), 1.28 (d, *J* = 6.5 Hz, 3 H) ppm; ^{13}C NMR (151 MHz, $CDCl_3$) δ_C 172.3, 162.2, 136.9, 136.6, 134.4, 129.4, 121.4, 118.4, 113.9, 78.7, 67.9, 18.3 ppm; ^{13}C NMR (151 MHz, CD_3OD) δ_C 172.5, 161.8, 138.3, 136.8, 134.7, 131.4, 121.8, 118.4, 116.5, 79.1, 68.1, 18.6 ppm; HRMS (ESI) *m/z* $[M + H]^+$ calcd for $C_{12}H_{13}O_4$: 221.0808, found: 221.0808; $[M + Na]^+$ calcd for $C_{12}H_{12}NaO_4$: 243.0628, found: 243.0628; $[M + K]^+$ calcd for $C_{12}H_{12}KO_4$: 259.0367, found: 259.0367.

(*R*)-3-((*S*)-1-hydroxyethyl)-1,3-dihydrobenzo[*c*]oxepin-9-ol (1)

To a solution of lactone **3a** (55 mg, 0.25 mmol) in 1,1,2,2-tetrachloroethane (1,6 mL) was added potassium tetrakis[3,5-bis(trifluoromethyl)phenyl]borate ($KBAr_4$) (9.0 mg, 0.01 mmol, 0.04 equiv) and phenylsilane (118 mg, 1.09 mmol, 5.0 equiv) and the reaction mixture was stirred at 100 °C under Argon atmosphere for 15 hours. The resulting solution was evaporated under vacuum and the residue was dissolved in anhydrous THF (5 mL). Subsequently, TBAF.3H_2O (315 mg, 2.18 mmol, 10.0 equiv) and AcOH (144 µL, 2.50 mmol, 10.0 equiv) were added to the prepared solution and the resulting mixture was left to stir at room temperature for 20 min. The mixture was then diluted with water (15 ml) and EtOAc (15 ml), and separated aqueous layer was

extracted with EtOAc (3 x 15 mL). Combined organic layers were dried over anhydrous Na_2SO_4, filtered and concentrated under reduced pressure. The residue was adsorbed on a silica gel and purified using MPLC (isocratic Hex/Et_2O: 1/1) providing desired product **1** (20 mg, 38% yield, yellow oil). TLC R_f 0.09 (Hex/Et_2O: 1/1); $[\alpha]_D^{25}$ + 57.5 (c 0.3, MeOH); IR (ATR) ν_{max} 3297, 2922, 2457, 1587, 1465, 1261, 722 cm^{-1}; ^1H NMR (400 MHz, CD_3OD) δ 7.03 (t, *J* = 7.8 Hz, 1H), 6.72 (d, *J* = 7.5 Hz, 1H), 6.67 (d, *J* = 8.1 Hz, 1H), 6.50 (dd, *J* = 12.5, 2.3 Hz, 1H), 5.96 (dd, *J* = 12.4, 2.5 Hz, 1H), 5.23 (d, *J* = 13.8 Hz, 1H), 4.45 (d, *J* = 13.8 Hz, 1H), 4.35 (dt, *J* = 5.0, 2.4 Hz, 1H), 3.77 (qd, *J* = 6.4, 5.3 Hz, 1H), 1.19 (d, *J* = 6.4 Hz, 3H); ^{13}C NMR (151 MHz, CD_3OD) δ_C 154.9, 139.0, 133.6, 131.4, 129.0, 127.1, 123.2, 115.1, 87.4, 70.8, 64.9, 19.1 ppm; HRMS (ESI) m/z $[M + H]^+$ calcd for $C_{12}H_{15}O_3$: 207.1016, found: 207.1015; $[M + Na]^+$ calcd for $C_{12}H_{14}NaO_3$: 229.0835, found: 229.0836; $[M + K]^+$ calcd for $C_{12}H_{12}KO_4$: 245.0575, found: 245.0574.

(*R*)-3-((*R*)-1-hydroxyethyl)-1,3-dihydrobenzo[*c*]oxepin-9-ol (18)

To a solution of lactone *ent*-**3b** (51 mg, 0.23 mmol) in 1,1,2,2-tetrachloroethane (1,6 mL) was added potassium tetrakis[3,5-bis(trifluoromethyl)phenyl]borate ($KBAr_4$) (8.4 mg, 0.009 mmol, 0.04 equiv.) and phenylsilane (100 mg, 0,926 mmol, 5.0 equiv) and the reaction mixture was stirred at 100 °C under Argon atmosphere for 15 hours. The resulting solution was evaporated under vacuum and the residue was dissolved in anhydrous THF (5 mL). Subsequently, TBAF.$3H_2O$ (730 mg, 2.32 mmol, 10.0 equiv) and AcOH (133 μl, 2.32 mmol, 10.0 equiv) were added to the prepared solution and the resulting mixture was left to stir at room temperature for 20 min. The mixture was then diluted with water (15 mL) and EtOAc (15 mL), and separated aqueous layer was extracted with EtOAc (3 x 15 mL). Combined organic layers were dried over anhydrous

Na_2SO_4, filtered and concentrated under reduced pressure. The residue was adsorbed on silica gel and purified using MPLC (isocratic Hex/Et_2O: 1/1) providing desired product **18** (17 mg, 36% yield, yellow oil). TLC R_f 0.11 (Hex/Et_2O: 1/1); $[\alpha]_D^{25}$ +62.2 (c 0.3, MeOH); ^1H NMR (400 MHz, CD_3OD) δ 7.03 (t, *J* = 7.8 Hz, 1H), 6.73 (d, *J* = 7.5 Hz, 1H), 6.67 (d, *J* = 8.1 Hz, 1H), 6.54 (dd, *J* = 12.6, 2.3 Hz, 1H), 5.89 (dd, *J* = 12.5, 2.5 Hz, 1H), 5.25 (d, *J* = 13.8 Hz, 1H), 4.46 (d, *J* = 13.8 Hz, 1H), 4.40 (dt, *J* = 5.0, 2.4 Hz, 1H), 3.85 (qd, *J* = 6.4, 5.3 Hz, 1H), 1.15 (d, *J* = 6.4 Hz, 3H); ^{13}C NMR (151 MHz, CD_3OD) δ_C 155.1, 139.0, 133.0, 132.1, 129.0, 127.2, 123.1, 115.2, 87.3, 70.3, 64.8, 18.7 ppm; IR (ATR) ν_{max} 3310, 2925, 2457, 1586, 1462, 1272, 724 cm^{-1}; HRMS (ESI) m/z $[M + H]^+$ calcd for $C_{12}H_{15}O_3$: 207.1016, found: 207.1014; [M + Na]+ calcd for $C_{12}H_{14}NaO_3$: 229.0835, found: 229.0836; [M + K]+ calcd for $C_{12}H_{12}KO_4$: 245.0575, found: 245.0574.
