# Supplementary material for: Synthetic Study of Natural Metabolites Containing a Benzo[c]oxepine Skeleton: Heterocornol C and D
Source: Int J Mol Sci. 2023 Jun 19;24(12):10331. doi: 10.3390/ijms241210331 (PMC10299168; doi:10.3390/ijms241210331)
Supplement: Supplementary file 1 [file ijms-24-10331-s001.zip › NMR spectra_heterocornol_D.pdf]

Supporting information – NMR spectra, HPLC analysis and crystal data for *ent*-17a

Synthetic Study of Natural Metabolites Containing a Benzo[*c*]oxepine Skeleton: Heterocornol C and D

Ján Gettler,[†] *Tomáš Čarný,* [†] *Martin Markovič,**[†‡] *Peter Koóš,** [†‡] *Erika Samoľová,*[#] *Ján Moncol*[§] *and Tibor Gracza*[†]

[†]Department of Organic Chemistry, Institute of Organic Chemistry, Catalysis and Petrochemistry, Slovak University of Technology, Radlinského 9, SK-812 37 Bratislava, Slovakia

[‡] Georganics Ltd., Koreničova 1, SK-811 03 Bratislava, Slovakia

[#] Institute of Physics of the Czech Academy of Science, Na Slovance 2, 182 21 Prague 8, Czech Republic

[§]Department of Inorganic Chemistry, Institute of Inorganic Chemistry, Technology and Materials, Slovak University of Technology, Radlinského 9, SK-812 37 Bratislava, Slovakia

Table of Content

ORTEP like representation and crystal data for the molecular structures

Figure S1. ORTEP like representation of *ent*-**17a** ………………………………… S4
Figure S2. A ball-and-stick view of crystal structure of *ent*-**17a**……………………...S4
Figure S3. Crystal structure of *ent*-**17a** showing C–H···O hydrogen bonds…………S5

Crystal data for *ent*-**17a** ……………………………………………………………… S5

Table S1. Hydrogen Bonds for *ent*-**17a** (IAM)……………………………………….S5
Table S2. Hydrogen Bonds for *ent*-**17a** (HAR)……………………………………….S6

Copies of ^{1}H and ^{13}C NMR spectra

Table S3. Comparison of the NMR data of synthetic and natural heterocornol D …...S7
Table S4. Comparison of the NMR data of synthetic and natural heterocornol C …...S8

Figure S4. ^{1}H NMR spectrum of **8** ……………………………………………… S9
Figure S5. ^{13}C NMR spectrum of **8** ……………………………………………… S10
Figure S6. ^{1}H NMR spectrum of **10** ……………………………………………… S11
Figure S7. ^{13}C NMR spectrum of **10** ……………………………………………… S12
Figure S8. ^{1}H NMR spectrum of *ent*-**10** ………………………………………… S13
Figure S9. ^{13}C NMR spectrum of *ent*-**10** ………………………………………… S14
Figure S10. ^{1}H NMR spectrum of **11** ……………………………………………… S15
Figure S11. ^{13}C NMR spectrum of **11** ……………………………………………… S16
Figure S12. ^{1}H NMR spectrum of *ent*-**11** ………………………………………… S17
Figure S13. ^{13}C NMR spectrum of *ent*-**11** ………………………………………… S18
Figure S14. ^{1}H NMR spectrum of **12** ……………………………………………… S19
Figure S15. ^{13}C NMR spectrum of **12** ……………………………………………… S20
Figure S16. ^{1}H NMR spectrum of ent-**12** ………………………………………… S21
Figure S17. ^{13}C NMR spectrum of ent-**12** ………………………………………… S22
Figure S18. ^{1}H NMR spectrum of **13** ……………………………………………… S23
Figure S19. ^{13}C NMR spectrum of **13** ……………………………………………… S24
Figure S20. ^{1}H NMR spectrum of *ent*-**13** ………………………………………… S25
Figure S21. ^{13}C NMR spectrum of *ent*-**13** ………………………………………… S26
Figure S22. ^{1}H NMR spectrum of **14a** ……………………………………………… S27
Figure S23. ^{13}C NMR spectrum of **14a** …………………………………………… S28
Figure S24. ^{1}H NMR spectrum of *ent*-**14a** ……………………………………… S29
Figure S25. ^{13}C NMR spectrum of *ent*-**14a** ……………………………………… S30
Figure S26. ^{1}H NMR spectrum of **15** ……………………………………………… S31
Figure S27. ^{13}C NMR spectrum of **15** ……………………………………………… S32
Figure S28. ^{1}H NMR spectrum of *ent*-**15** ………………………………………… S33
Figure S29. ^{13}C NMR spectrum of *ent*-**15** ………………………………………… S34
Figure S30. ^{1}H NMR spectrum of **14b** ……………………………………………… S35
Figure S31. ^{13}C NMR spectrum of **14b** …………………………………………… S36
Figure S32. ^{1}H NMR spectrum of *ent*-**14b** ……………………………………… S37
Figure S33. ^{13}C NMR spectrum of *ent*-**14b** ……………………………………… S38
Figure S34. ^{1}H NMR spectrum of **16a** ……………………………………………… S39
Figure S35. ^{13}C NMR spectrum of **16a** …………………………………………… S40

Figure S36. ^{1}H NMR spectrum of *ent*-**16a** S41
Figure S37. ^{13}C NMR spectrum of *ent*-**16a** S42
Figure S38. ^{1}H NMR spectrum of **16b** S43
Figure S39. ^{13}C NMR spectrum of **16b** S44
Figure S40. ^{1}H NMR spectrum of *ent*-**16b** S45
Figure S41. ^{13}C NMR spectrum of *ent*-**16b** S46
Figure S42. ^{1}H NMR spectrum of **17a** S47
Figure S43. ^{13}C NMR spectrum of **17a** S48
Figure S44. ^{1}H NMR spectrum of *ent*-**17a** S49
Figure S45. ^{13}C NMR spectrum of *ent*-**17a** S50
Figure S46. ^{1}H NMR spectrum of **17b** S51
Figure S47. ^{13}C NMR spectrum of **17b** S52
Figure S48. ^{1}H NMR spectrum of *ent*-**17b** S53
Figure S49. ^{13}C NMR spectrum of *ent*-**17b** S54
Figure S50. ^{1}H NMR spectrum of **3a** in $CDCl_3$ S55
Figure S51. ^{13}C NMR spectrum of **3a** in $CDCl_3$ S56
Figure S52. ^{1}H NMR spectrum of **3a** in MeOH-d_4 .... S57
Figure S53. ^{13}C NMR spectrum of **3a** in MeOH-d_4 .... S58
Figure S54. ^{1}H NMR spectrum of *ent*-**3a** in $CDCl_3$ S59
Figure S55. ^{13}C NMR spectrum of *ent*-**3a** in $CDCl_3$ S60
Figure S56. ^{1}H NMR spectrum of *ent*-**3a** in MeOH-d_4 .... S61
Figure S57. ^{13}C NMR spectrum of *ent*-**3a** in MeOH-d_4 .... S62
Figure S58. ^{1}H NMR spectrum of **3b** in $CDCl_3$ S63
Figure S59. ^{13}C NMR spectrum of **3b** in $CDCl_3$ S64
Figure S60. ^{1}H NMR spectrum of **3b** in MeOH-d_4 .... S65
Figure S61. ^{13}C NMR spectrum of **3b** in MeOH-d_4 .... S66
Figure S62. ^{1}H NMR spectrum of *ent*-**3b** in $CDCl_3$ S67
Figure S63. ^{13}C NMR spectrum of *ent*-**3b** in $CDCl_3$ S68
Figure S64. ^{1}H, NMR spectrum of *ent*-**3b** in MeOH-d_4 .... S69
Figure S65. ^{13}C NMR spectrum of *ent*-**3b** in MeOH-d_4 .... S70
Figure S66. ^{1}H NMR spectrum of **1** S71
Figure S67. ^{13}C NMR spectrum of **1** S72
Figure S68. ^{1}H, NMR spectrum of **18** S73
Figure S69. ^{13}C NMR spectrum of **18** S74

HPLC analysis of 3a and 3b.

HPLC Analytical method.. S75
Figure S70. *HPLC analysis of a mixture of 3a and ent-3a*.. S76
Figure S71. *HPLC analysis of synthetic 3a* S77
Figure S72. *HPLC analysis of racemic mixture of 3b and ent-3b* S78
Figure S73. *HPLC analysis of synthetic 3b* S79

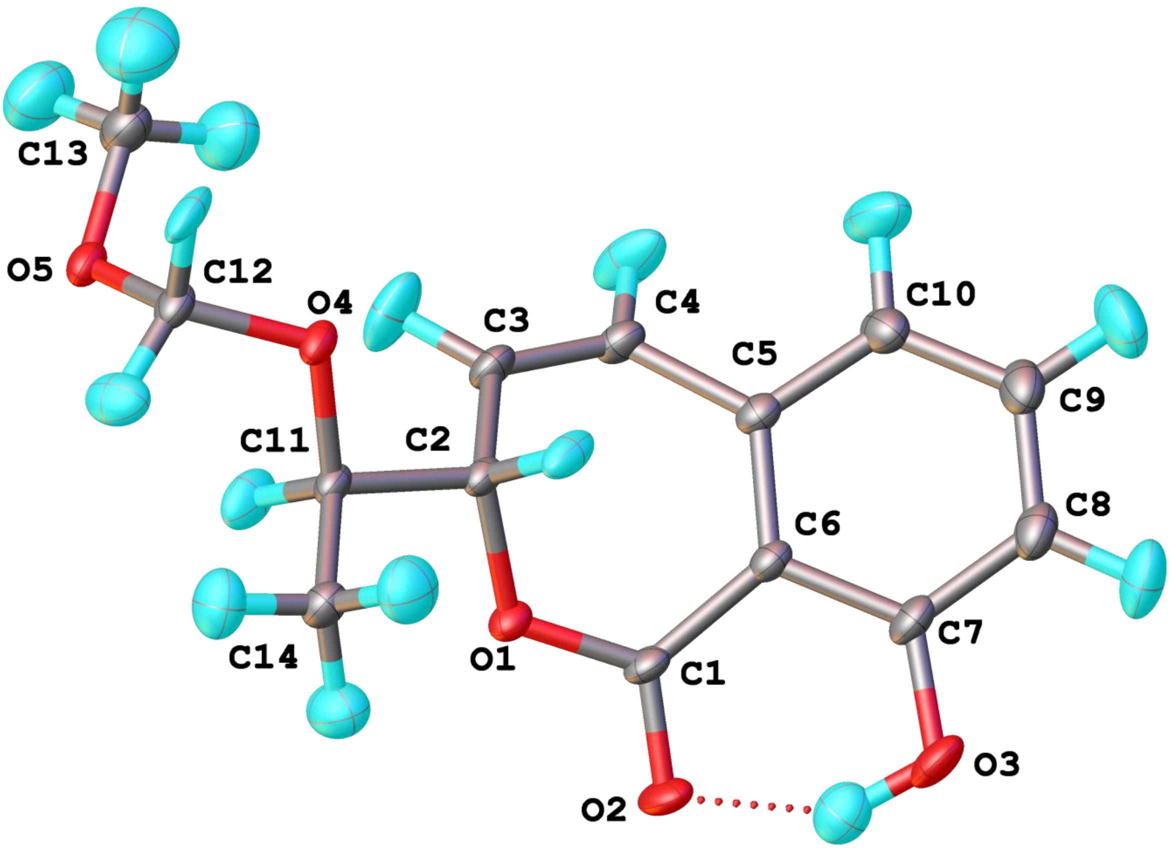

Figure S1. An ORTEP view of *ent*-**17a** showing the atom-numbering scheme of non-hydrogen atoms. Displacement ellipsoids are plotted at the 50% probability level.

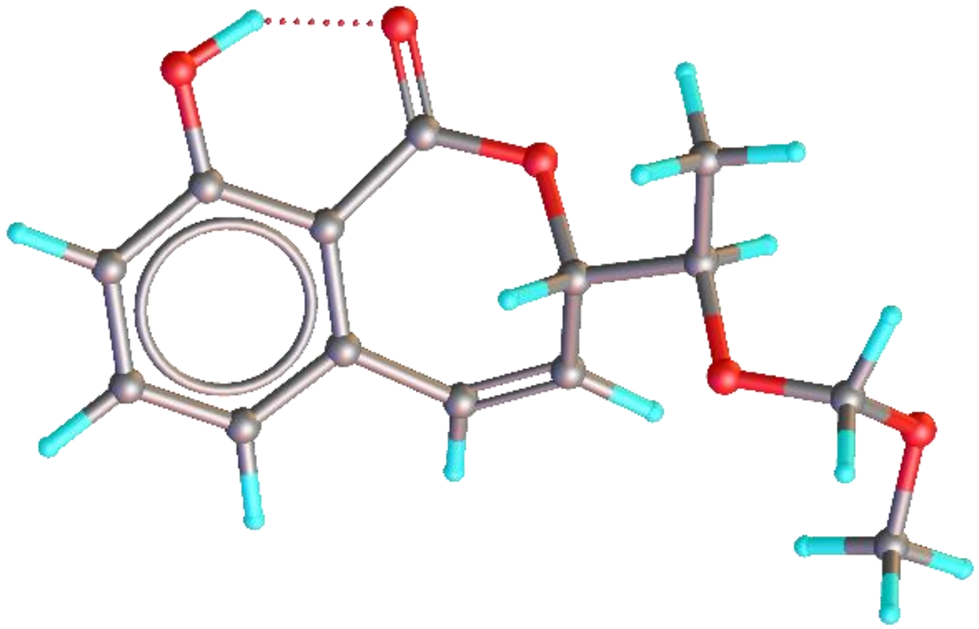

Figure S2. A ball-and-stick view of crystal structure of *ent*-**17a**.

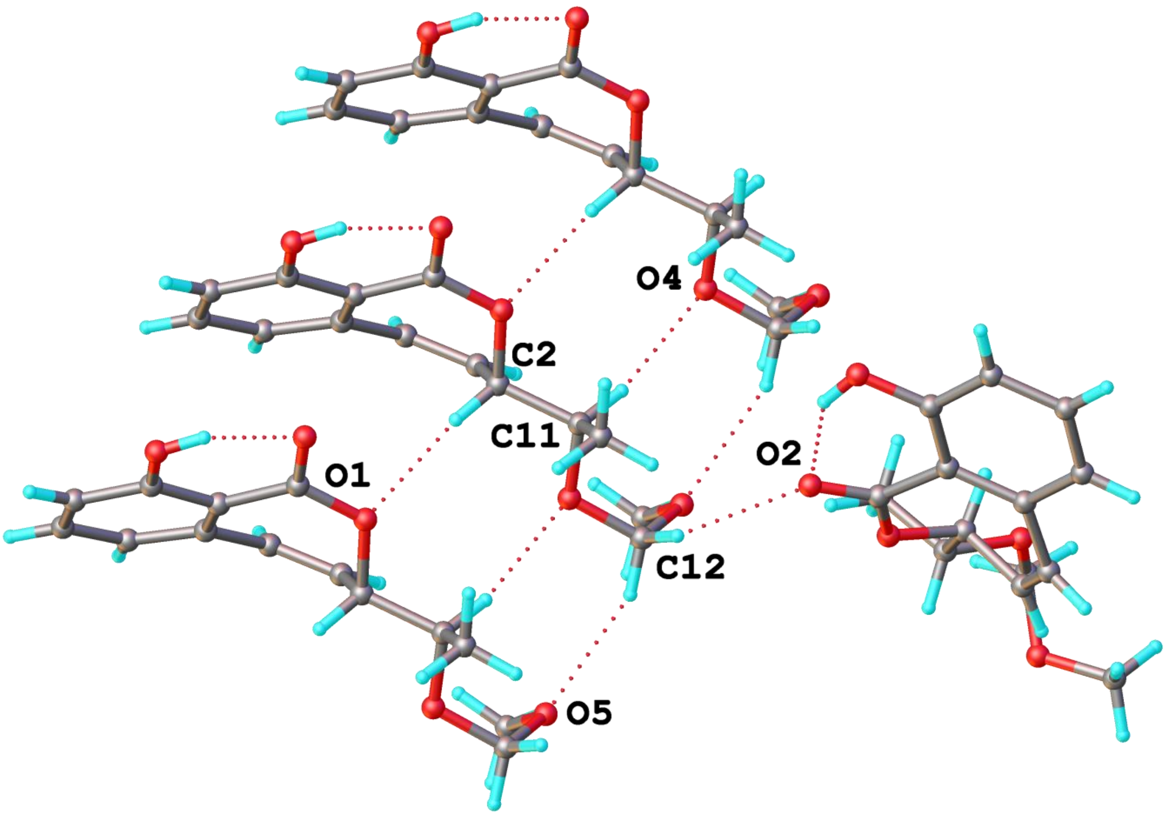

Figure S3. Crystal structure of *ent*-**17a** showing C–H···O hydrogen bonds.

Crystal Data for *ent*-**17a**: $C_{14}H_{16}O_5$ (M =264.27 g/mol): monoclinic, space group $P2_1$ (no. 4), a = 4.27815(5) Å, b = 12.64187(14) Å, c = 11.93812(17) Å, β = 98.3328(15)°, V = 638.843(17) Å^3, Z = 2, T = 95 K, μ(CuKα) = 0.873 mm^{-1}, $Dcalc$ = 1.374 g/cm^3, 12519 reflections measured (7.484° ≤ 2Θ ≤ 147.14°), 2520 unique (R_{int} = 0.0377, R_{sigma} = 0.0266) which were used in all calculations. IAM: The final R_1 was 0.0269 (I > 2σ(I)) and wR_2 was 0.0675 (all data). CCDC no. 2222838. HAR: The final R_1 was 0.0184 (I > 2σ(I)) and wR_2 was 0.0404 (all data). CCDC no. 2222839. The Flack parameters for the IAM nad HAR model have the value of x = 0.01(7) and 0.07(7), respectively. The Hooft parameter has a value of y = 0.04(6) for both models.

Table S1. Hydrogen Bonds for *ent*-**17a** (IAM).

D	H	A	d(D-H)/Å	d(H-A)/Å	d(D-A)/Å	D-H-A/°
O3	H3	O2	0.8400	1.8161(17)	2.5503(15)	145.03(13)
C11	H11	O4[1]	1.0000	2.4857(17)	3.4719(17)	168.75(3)
C2	H2	O1[2]	1.0000	2.5860(16)	3.5763(16)	170.62(4)
C12	H12a	O5[2]	0.9900	2.4544(16)	3.3739(16)	154.31(3)
C12	H12b	O2[3]	0.9900	2.5997(16)	3.4750(17)	147.41(4)

[1]-1+X,+Y,+Z; [2]1+X,+Y,+Z; [3]1-X,1/2+Y,1-Z

Table S2. Hydrogen Bonds for *ent*-**17a** (HAR).

D	H	A	d(D-H)/Å	d(H-A)/Å	d(D-A)/Å	D-H-A/°
O3	H3	O2	0.990(9)	1.664(11)	2.5451(11)	145.9(13)
C11	H11	$O4^{1}$	1.104(8)	2.387(8)	3.4763(12)	168.8(9)
C2	H2	$O1^{2}$	1.105(8)	2.488(8)	3.5814(12)	170.0(9)
C12	H12a	$O5^{2}$	1.099(8)	2.327(8)	3.3774(12)	159.2(8)
C12	H12b	$O2^{3}$	1.109(8)	2.472(9)	3.4762(12)	149.8(10)

^{1}-1+X,+Y,+Z; ^{2}1+X,+Y,+Z; ^{3}1-X,1/2+Y,1-Z

Table S3. Comparison of the NMR data of synthetic and natural heterocornol D.

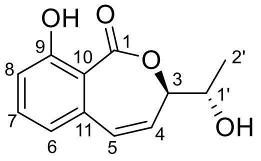

heterocornol D

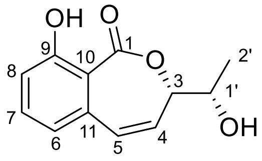

diastereoisomer (**3b**)

position	**natural** (Lei et al.[8])	**synthetic** heterocornol D (**3a**)	diastereoisomer **3b**
	δ_H (m, *J* [Hz])	δ_H (m, *J* [Hz])	δ_H (m, *J* [Hz])
1	-	-	-
3	4.45 (brt, 5.8)	4.46 (ddd, 5.8, 4.7, 1.8)	4.46 (td, 5.8, 1.7)
4	6.42 (dd, 10.3, 5.8)	6.41 (dd, 10.4, 5.8)	6.34 (dd, 10.3, 5.9)
5	6.97 (brd, 10.3)	6.98 (brd, 10.4)	6.95 (brd, 10.4)
6	6.88 (d, 8.1)	6.88 (d, 7.6)	6.88 (d, *J* = 7.5)
7	7.45 (t, 7.9)	7.45 (t, 7.9)	7.45 (t, 7.9)
8	6.97 (d, 7.9)	6.98 – 6.96 (m)	6.97 (dd, 8.2, 1.1)
9	-	-	-
10	-	-	-
11	-	-	-
1’	4.08 (m)	4.10 – 4.05 (m)	4.06 (quint., 6.4)
2’	1.27 (d, 6.4)	1.27 (d, 6.5)	1.28 (d, 6.5)

position	**natural** (Lei et al.[8])	**synthetic** heterocornol D (**3a**)	diastereoisomer **3b**
	δ_C	δ_C*	δ_C
1	171.0, C	172.5	172.5
3	77.5, CH	79.0	79.1
4	135.3, CH	136.8	136.8
5	129.7, CH	131.2	131.4
6	120.3, CH	121.8	121.8
7	133.2, CH	134.7	134.7
8	116.9, CH	118.3	118.4
9	160.3, C	161.8	161.8
10	115.0, C	116.5	116.5
11	136.9, C	138.4	138.3
1’	66.8, CH	68.3	68.1
2’	17.4, CH_3	18.8	18.6

* the difference of + 1.4 ppm in shifts for all signals compared to natural compound was caused by different locking of NMR spectra. The ^{13}C NMR spectrum of synthetic compound was locked to residual solvent peak (MeOH 49.00 ppm).

Table S4. Comparison of the NMR data of synthetic and natural heterocornol C.

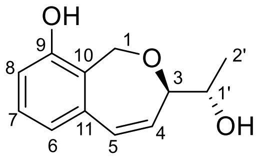

heterocornol C

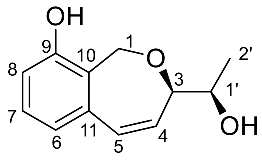

diastereoisomer **18**

position	**natural** (Lei et al.[8])	**synthetic** heterocornol C (**1**)	diastereoisomer **18**
	δ_H (m, *J* [Hz])	δ_H (m, *J* [Hz])	δ_H (m, *J* [Hz])
1	5.23 (d, 13.7)	5.23 (d, 13.8)	5.25, (d, 13.8)
3	4.35 (m)	4.35 (dt, 5.0, 2.4)	4.40 (dt, 5.0, 2.4)
4	5.96, dd (12.4, 2.0)	5.96 (dd, 12.4, 2.5)	5.89 (dd, 12.5, 2.5)
5	6.50 (brd, 12.4)	6.50 (dd, 12.5, 2.3)	6.54 (dd, 12.5, 2.3)
6	6.72 (d, 7.5)	6.72 (d, 7.5)	6.73, d (7.5)
7	7.02 (t, 7.5)	7.03 (t, 7.8)	7.03, t (7.8)
8	6.66 (d, 7.5)	6.67 (d, 8.1)	6.67, d (8.1)
9	-	-	-
10	4.44 (d, 13.7)	4.45 (d, 13.8)	4.46, (d, 13.8)
11	-	-	-
1’	3.76 (m)	3.77 (qd, 6.4, 5.3)	3.85 (qd, 6.4, 5.3)
2’	1.19 (d, 6.4)	1.19 (d, 6.4)	1.15 (d, 6.4)

position	**natural** (Lei et al.[8])	**synthetic** heterocornol C (**1**)	diastereoisomer **18**
	δ_C	δ_C*	δ_C
1	63.5, CH_2	64.9, CH_2	64.8, CH_2
3	86.1, CH	87.4, CH	87.3, CH
4	132.2, CH	133.6, CH	133.0, CH
5	130.1, CH	131.4, CH	132.1 CH
6	121.8, CH	123.2, CH	123.1, CH
7	127.6, CH	129.0, CH	129.0, CH
8	113.8, CH	115.1, CH	115.2, CH
9	153.5, C	154.9, C	155.1, C
10	125.7, C	127.1, C	127.2, C
11	137.6, C	139.0, C	139.0, C
1’	69.4, CH	70.8, CH	70.3, CH
2’	17.7, CH_3	19.1, CH_3	18.7, CH_3

* the difference of + 1.4 ppm in shifts for all signals compared to natural compound was caused by different locking of NMR spectra. The ^{13}C NMR spectrum of synthetic compound was locked to residual solvent peak (MeOH 49.00 ppm).

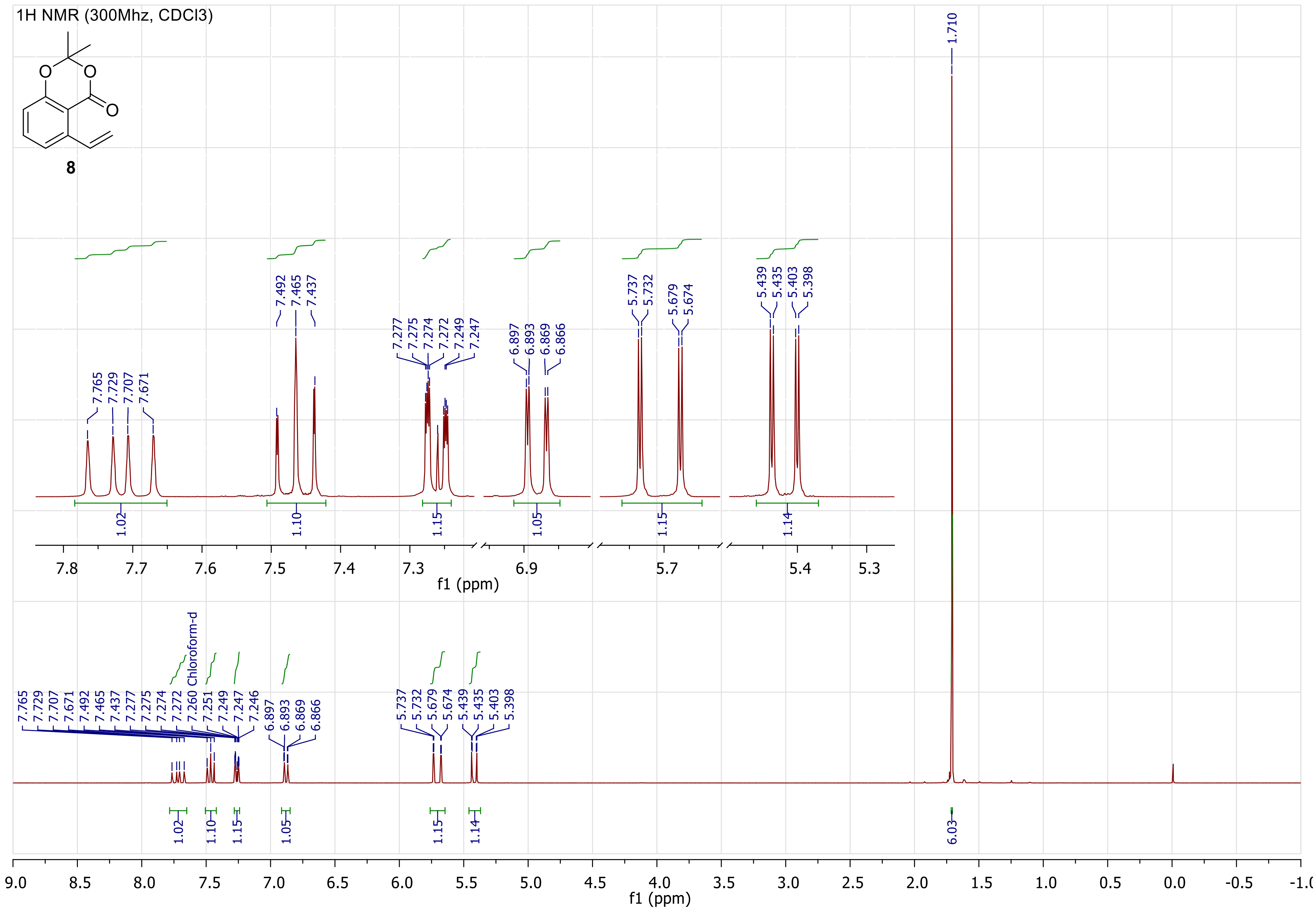

Figure S4. ^{1}H NMR spectrum of **8**.

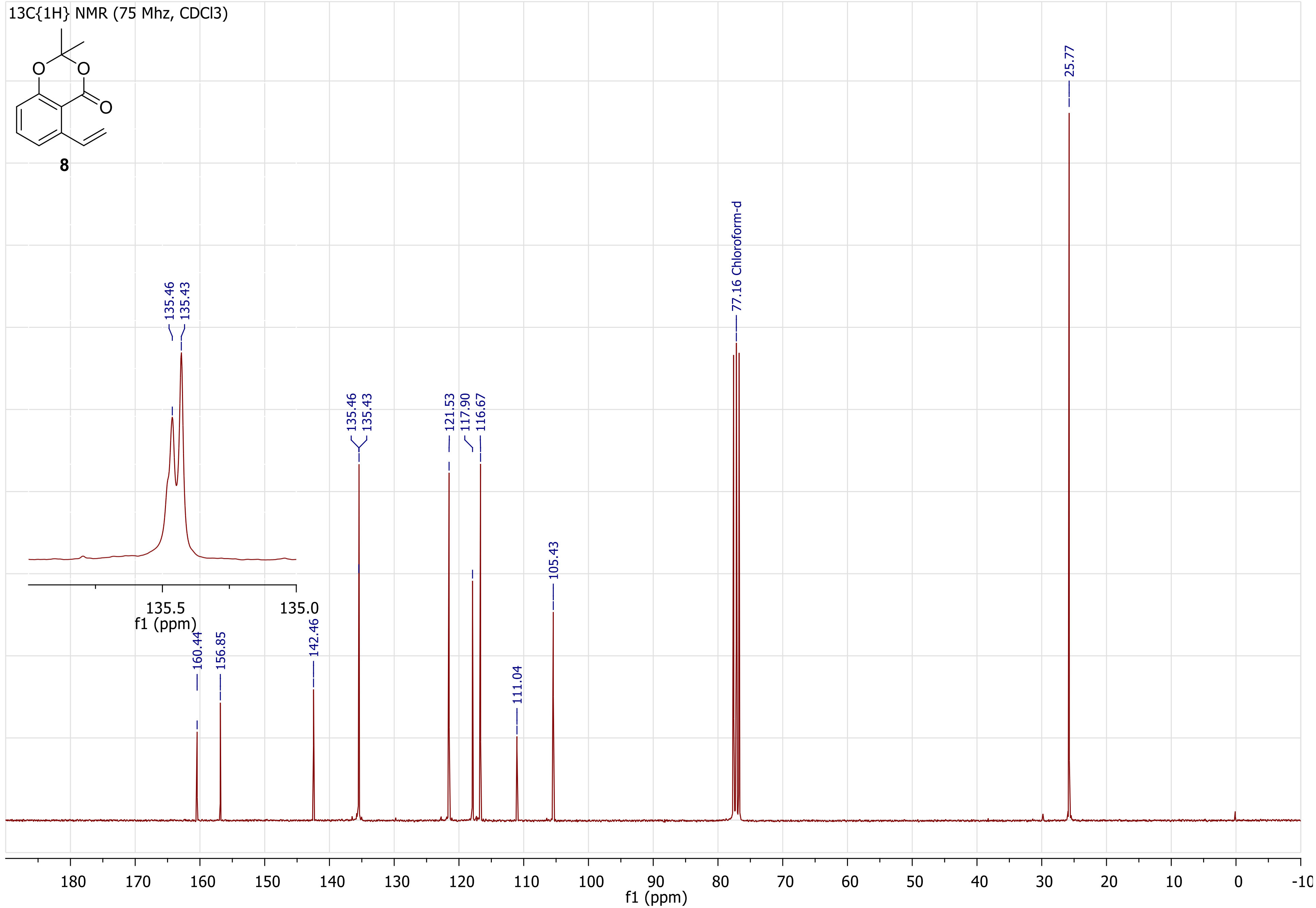

Figure S5. ^{13}C NMR spectrum of **8**.

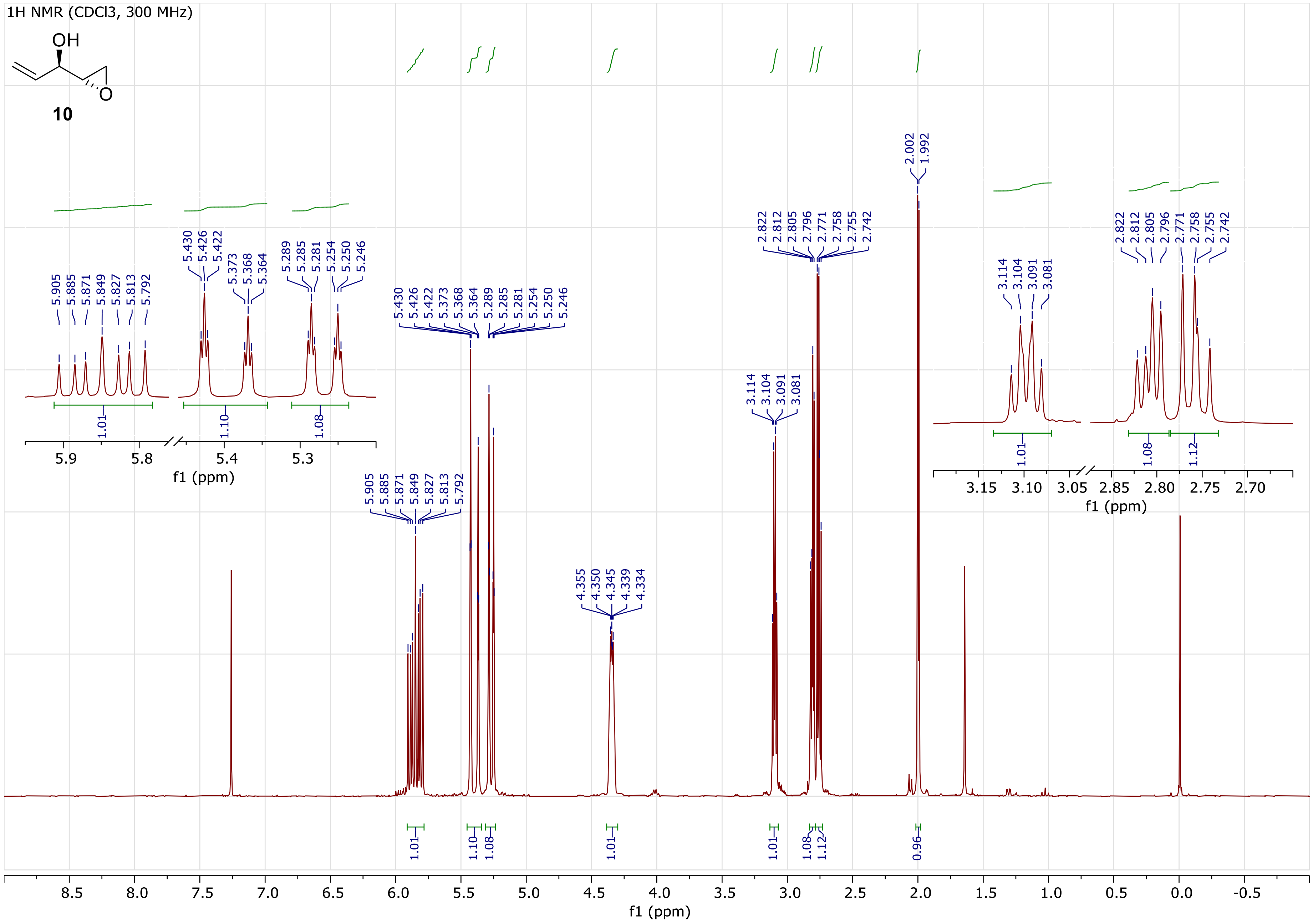

Figure S6. ^{1}H NMR spectrum of **10**.

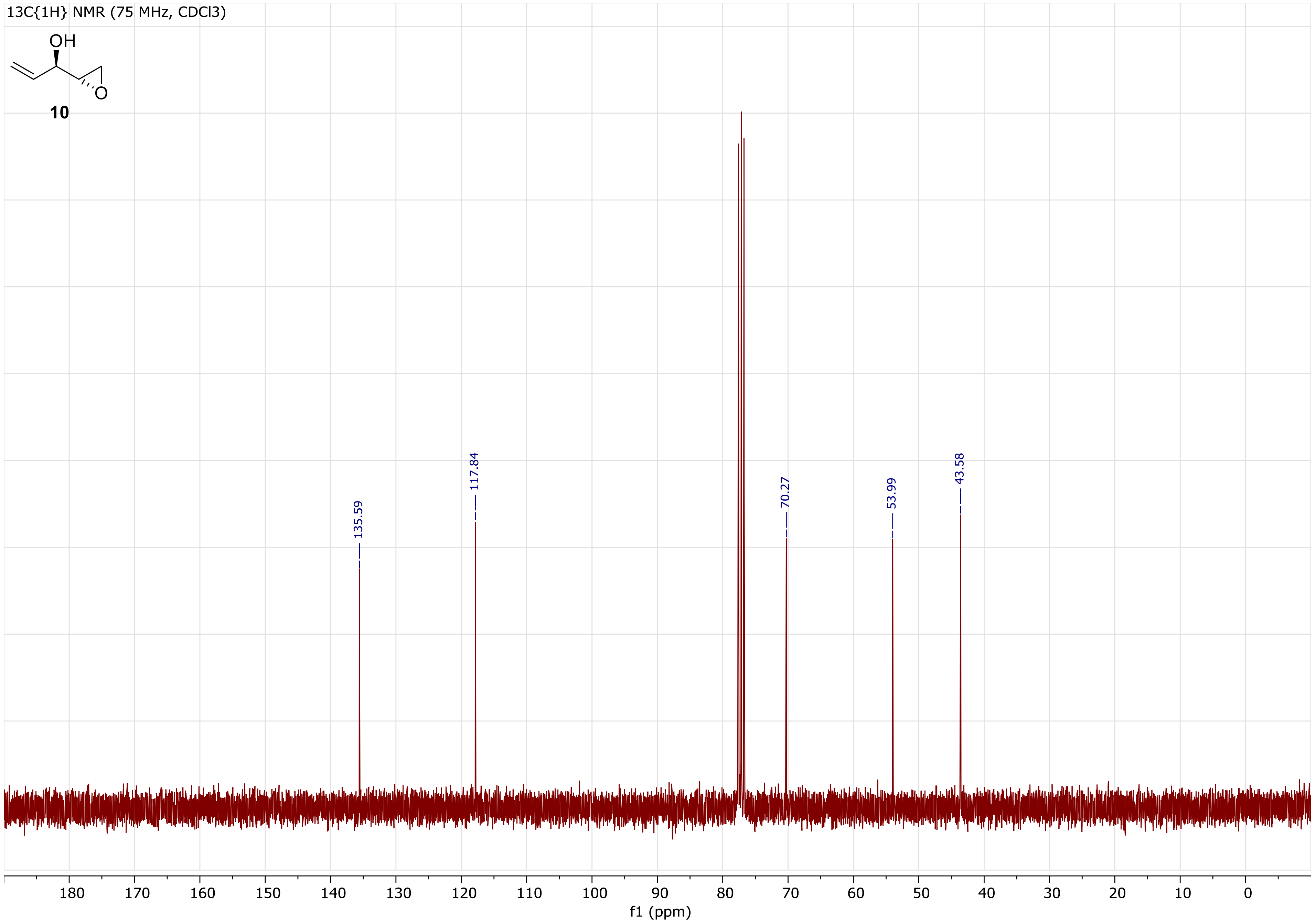

Figure S7. ^{13}C NMR spectrum of **10**.

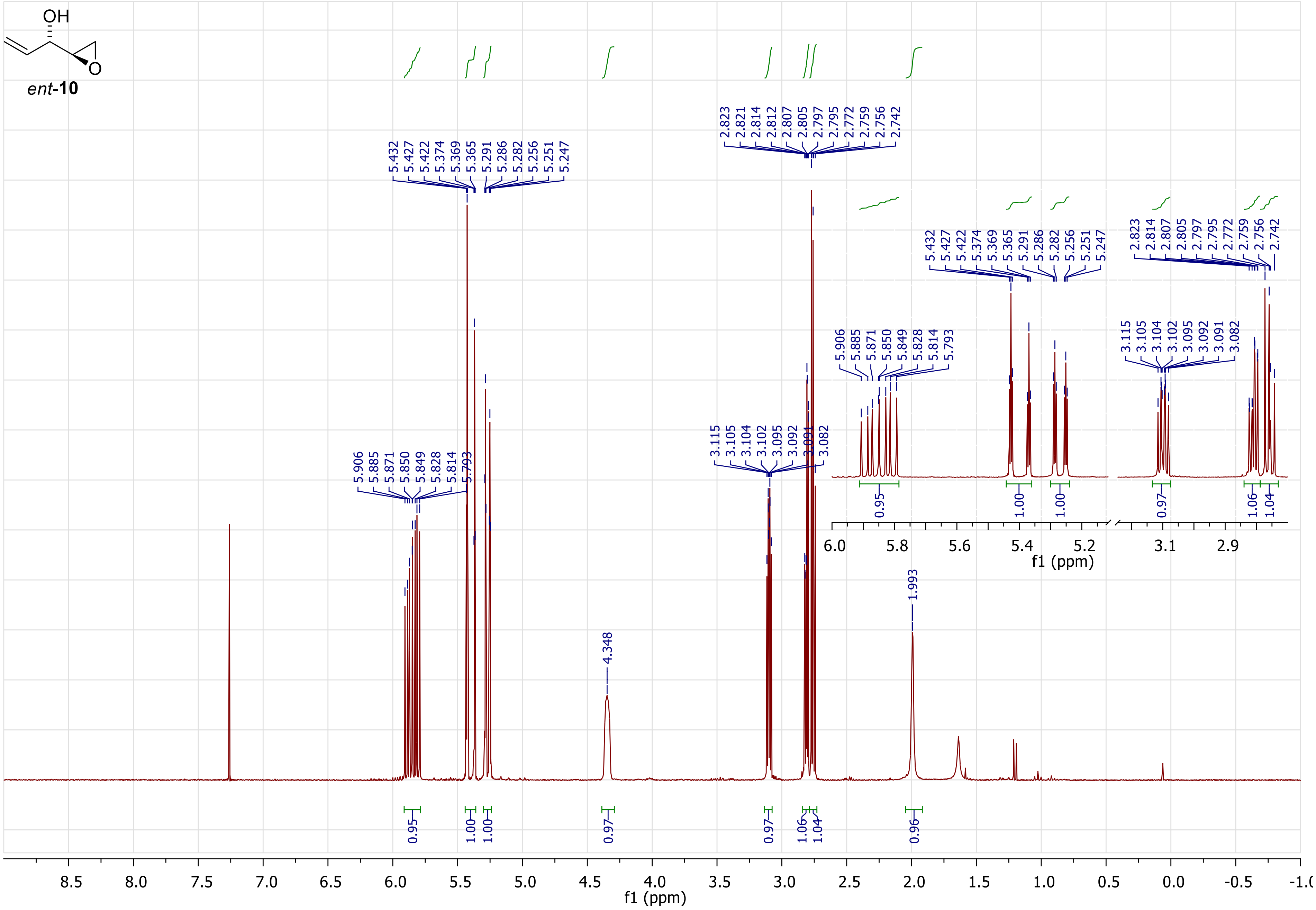

Figure S8. ^{1}H NMR spectrum of *ent*-**10**.

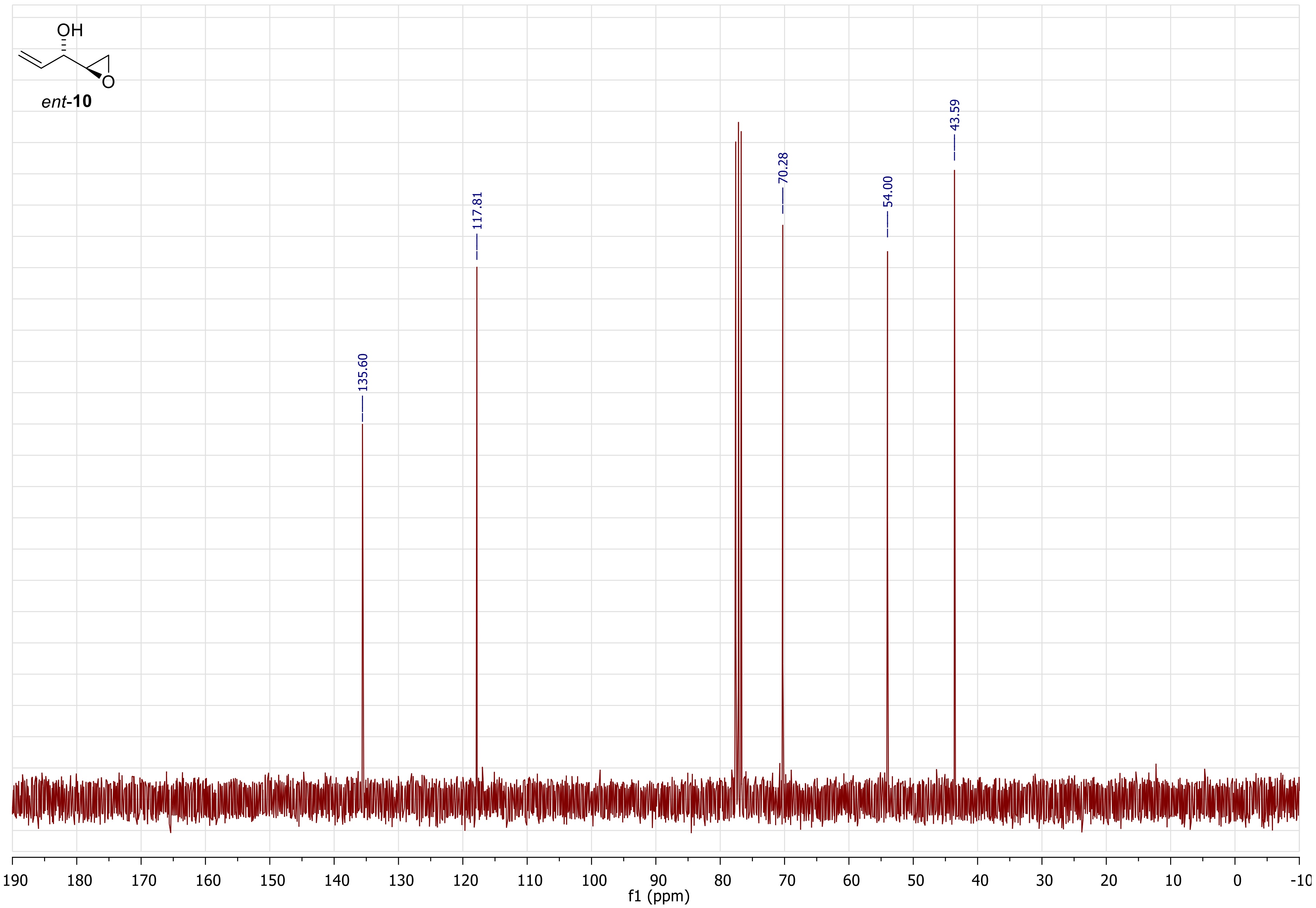

Figure S9. ^{13}C NMR spectrum of *ent*-**10**.

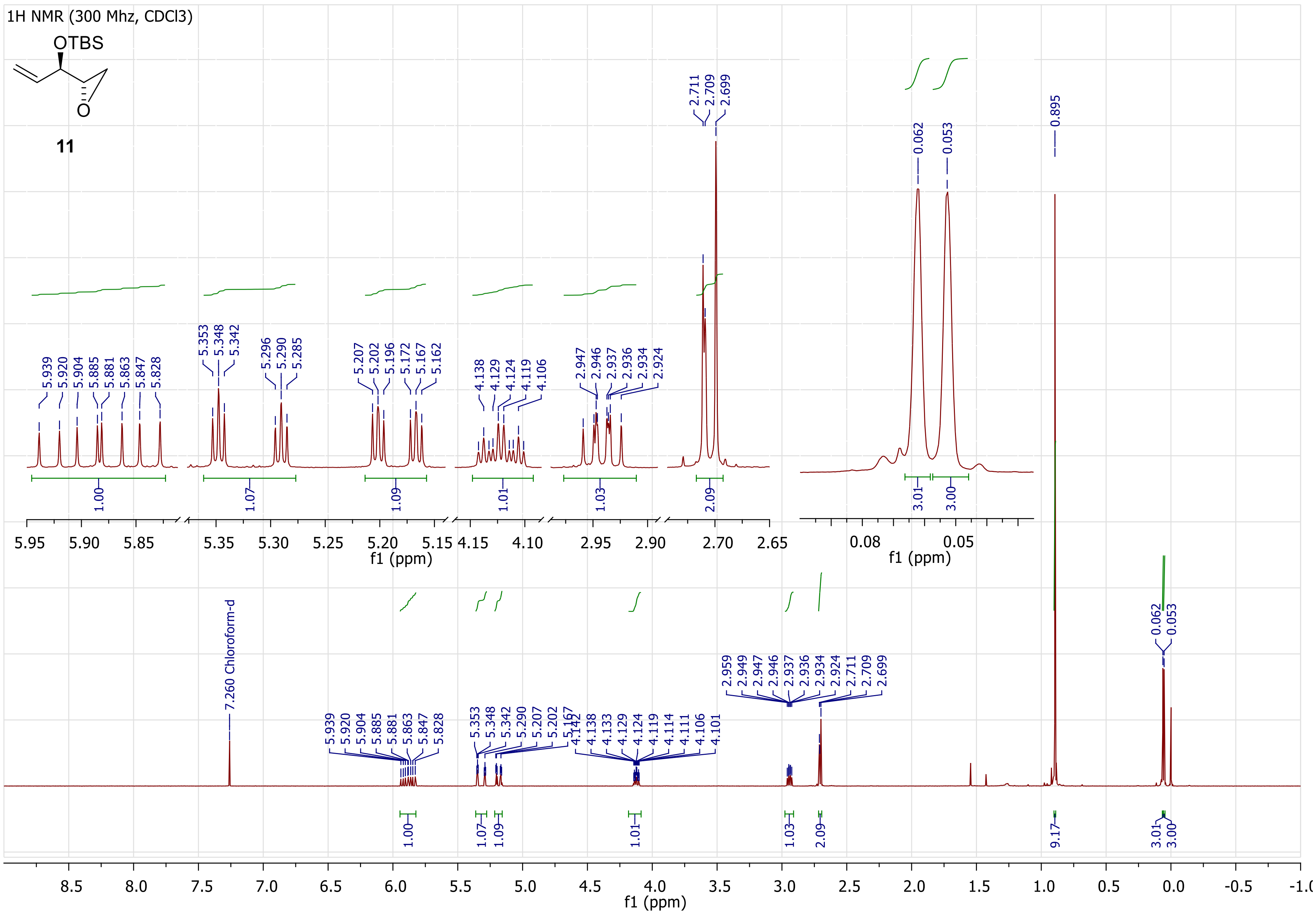

Figure S10. ^{1}H NMR spectrum of **11**.

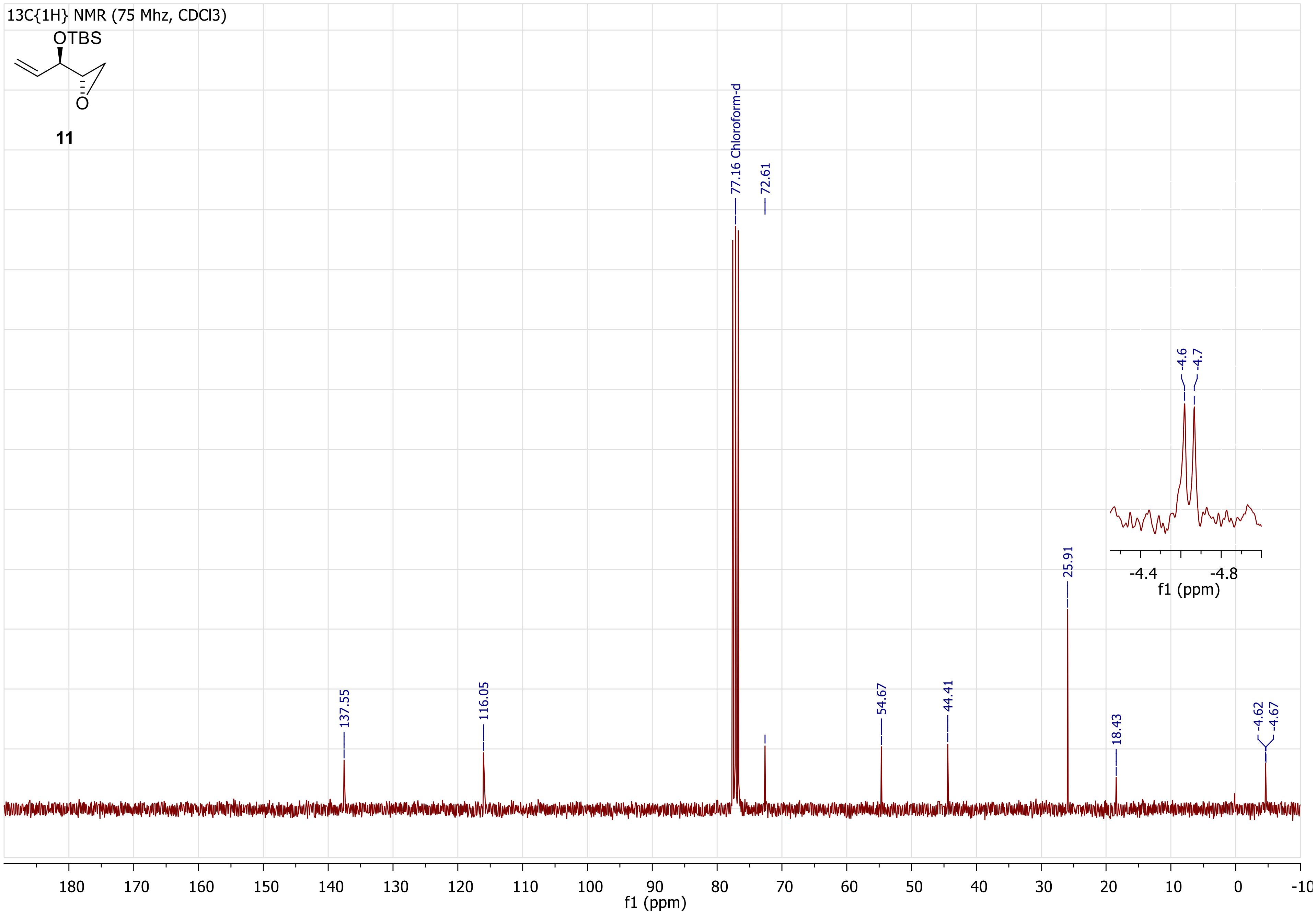

Figure S11. ^{13}C NMR spectrum of **11**.

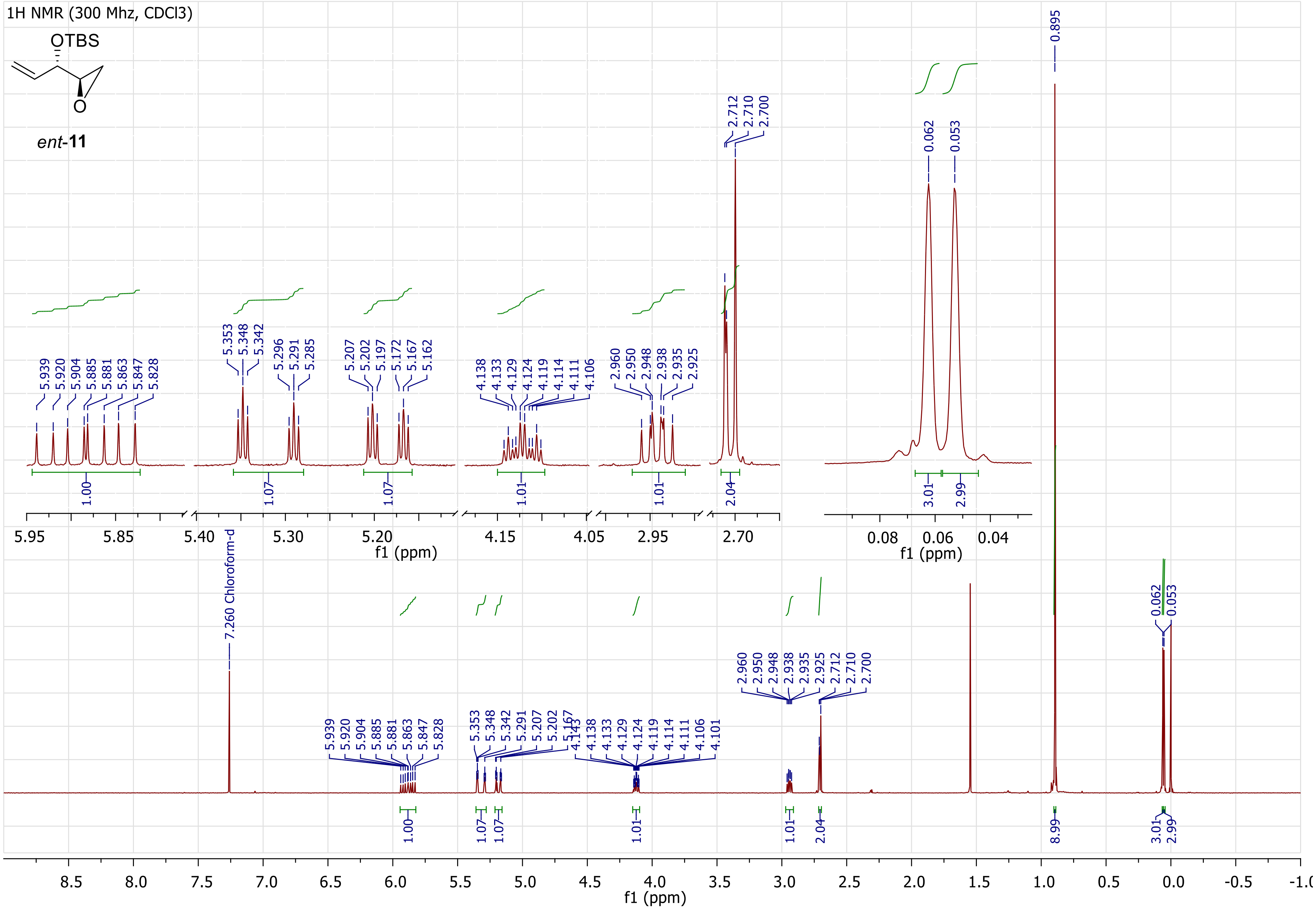

Figure S12. ^{1}H NMR spectrum of *ent*-**11**.

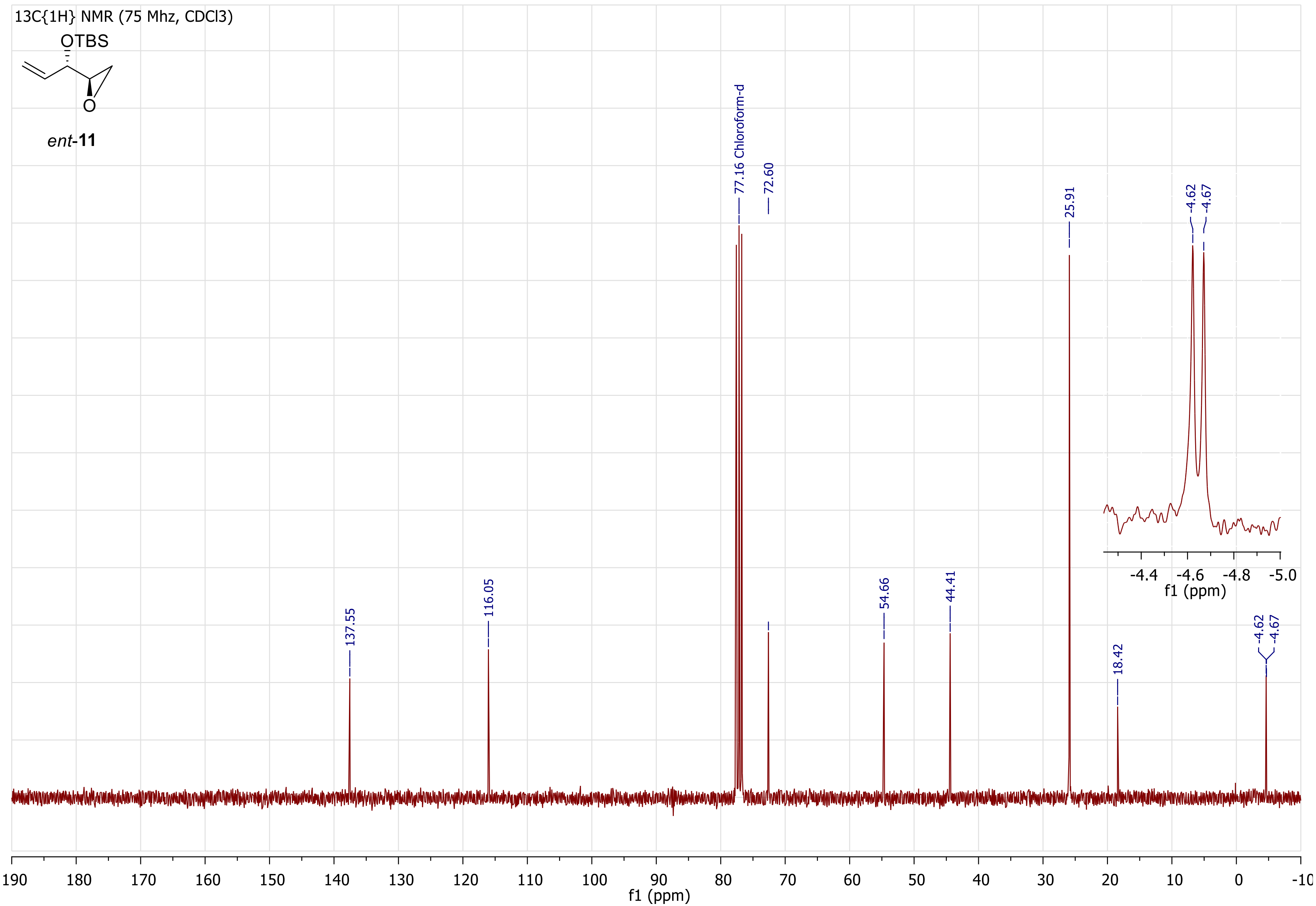

Figure S13. ^{13}C NMR spectrum of *ent*-**11**.

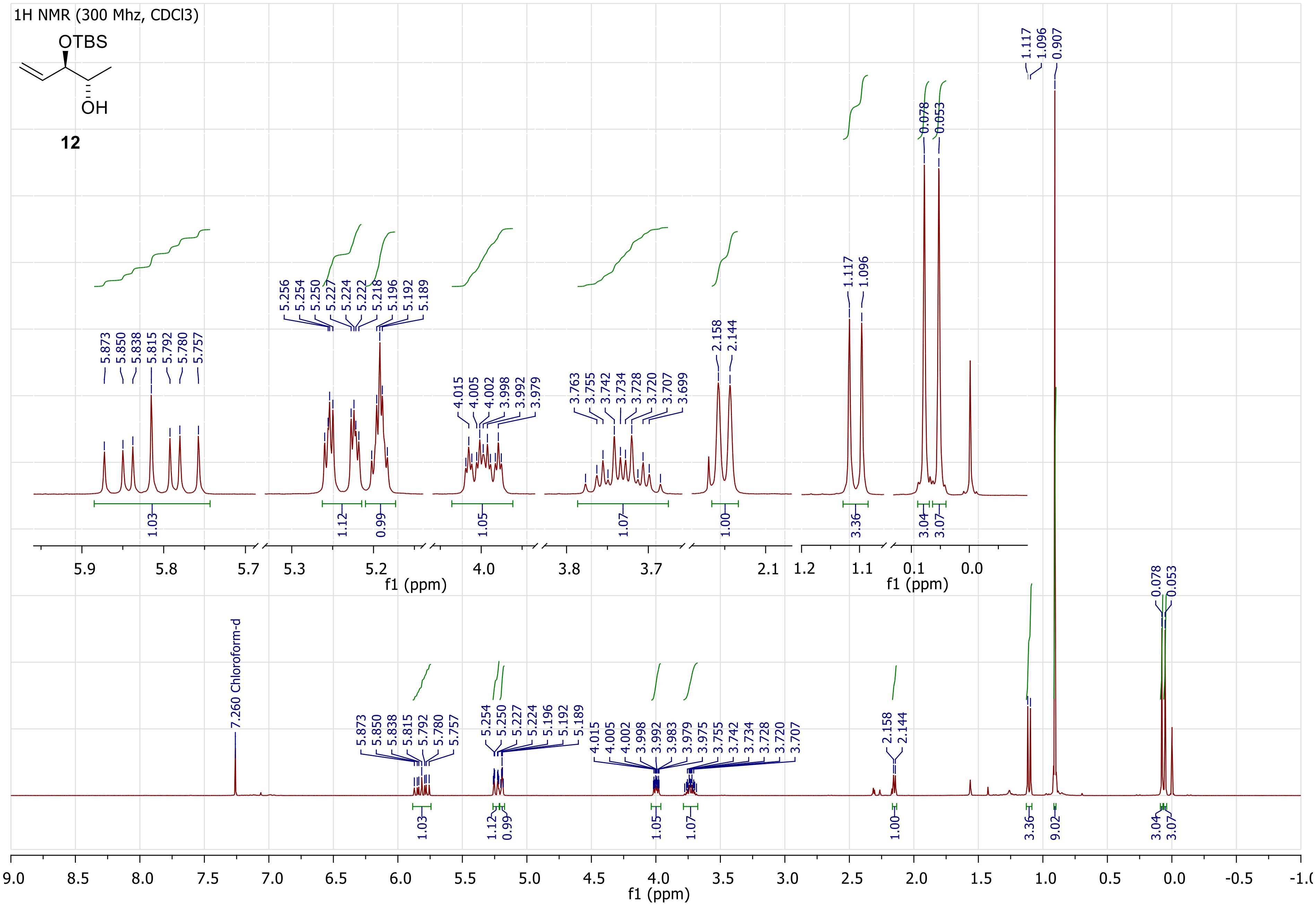

Figure S14. ^{1}H NMR spectrum of **12**.

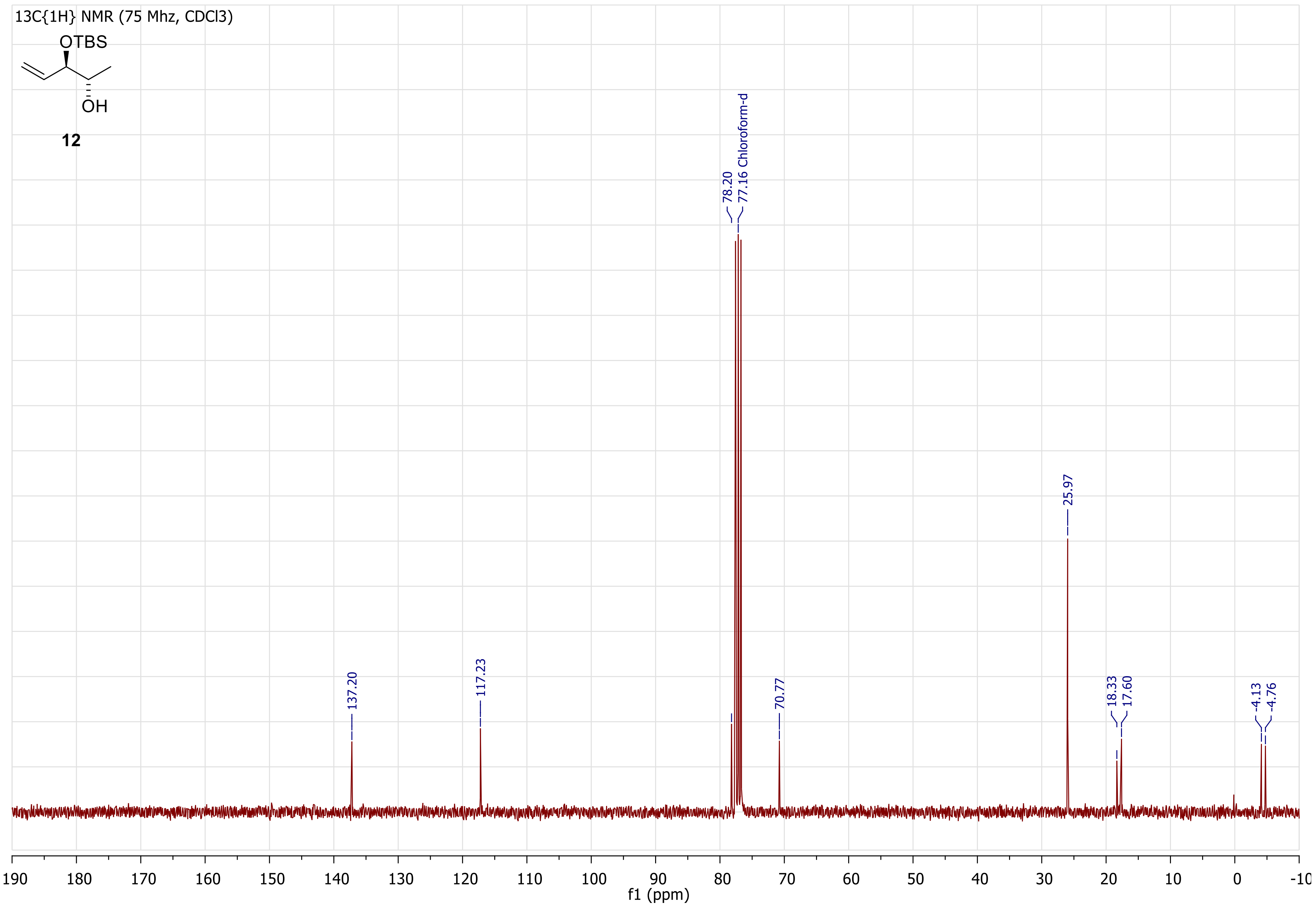

Figure S15. ^{13}C NMR spectrum of **12**.

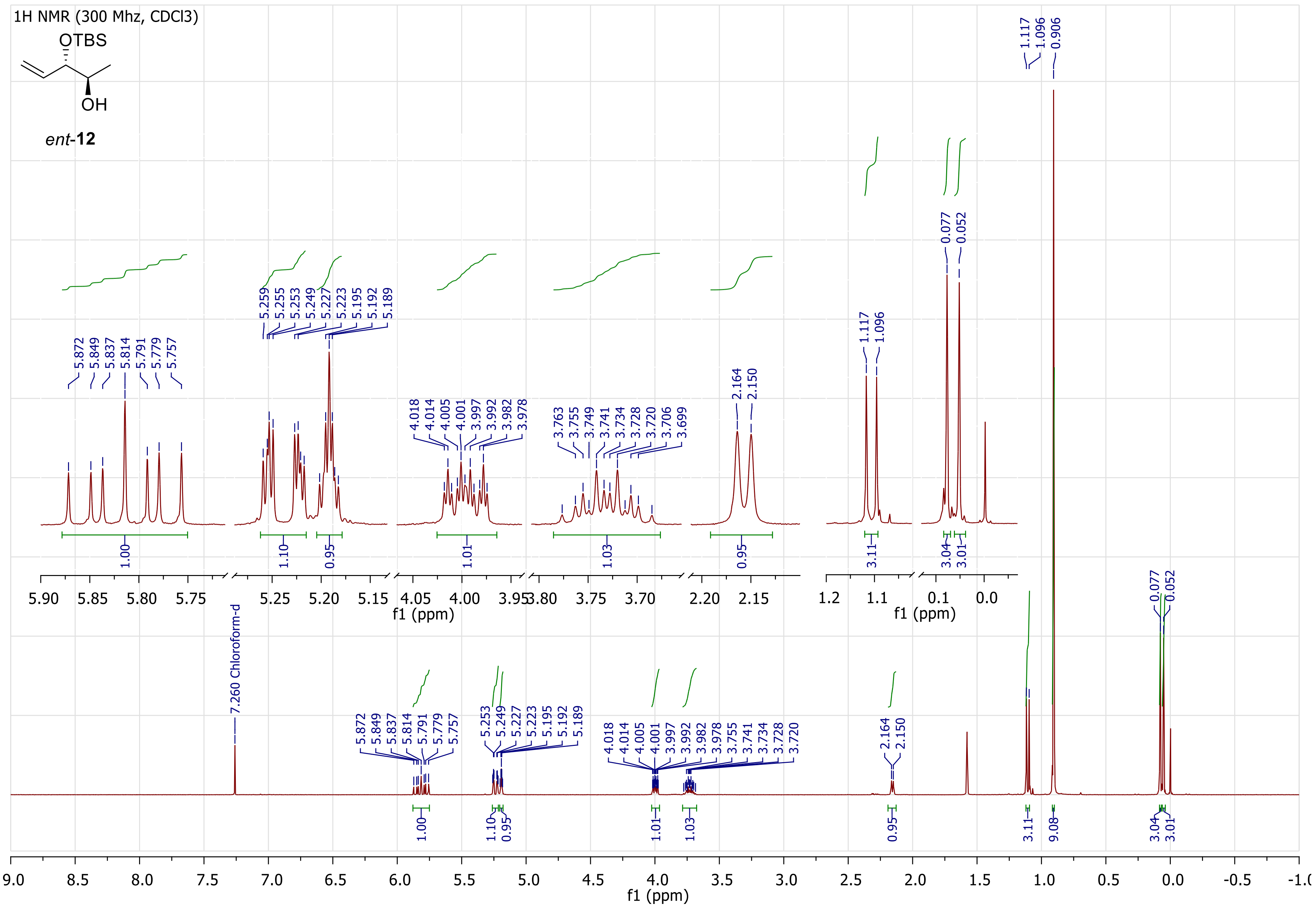

Figure S16. ^{1}H NMR spectrum of *ent*-**12**.

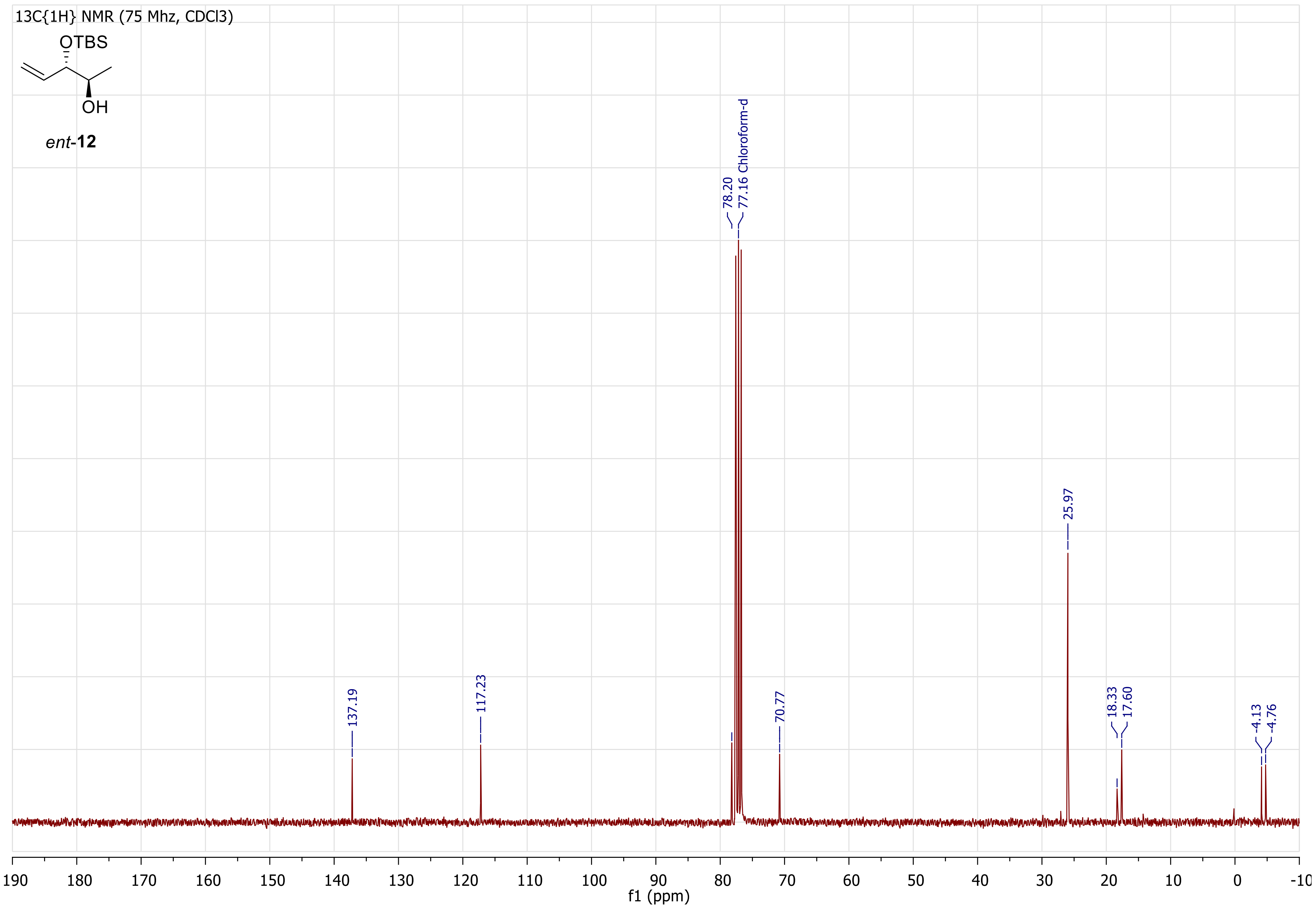

Figure S17. ^{13}C NMR spectrum of *ent*-**12**.

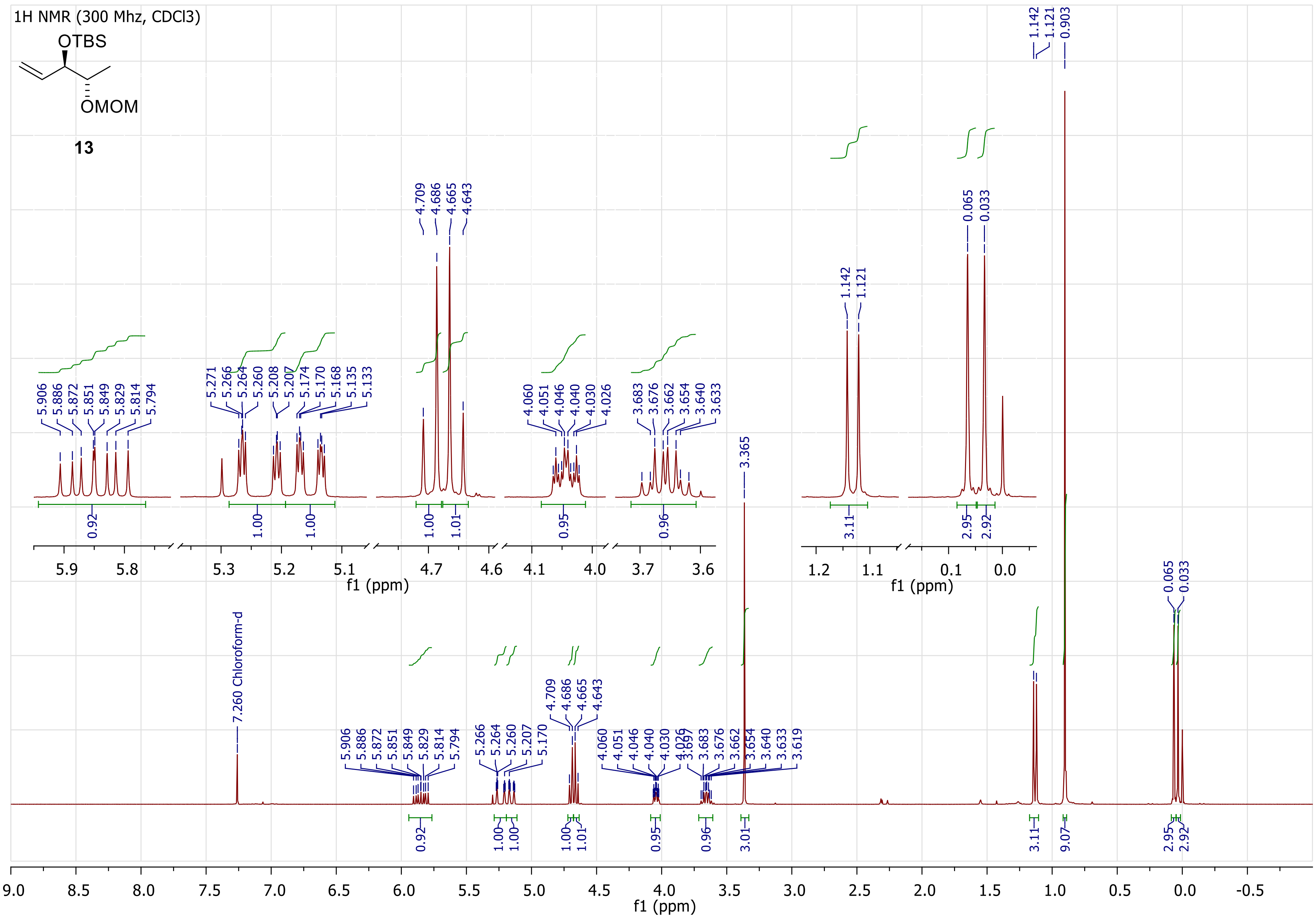

Figure S18. ^{1}H NMR spectrum of **13**.

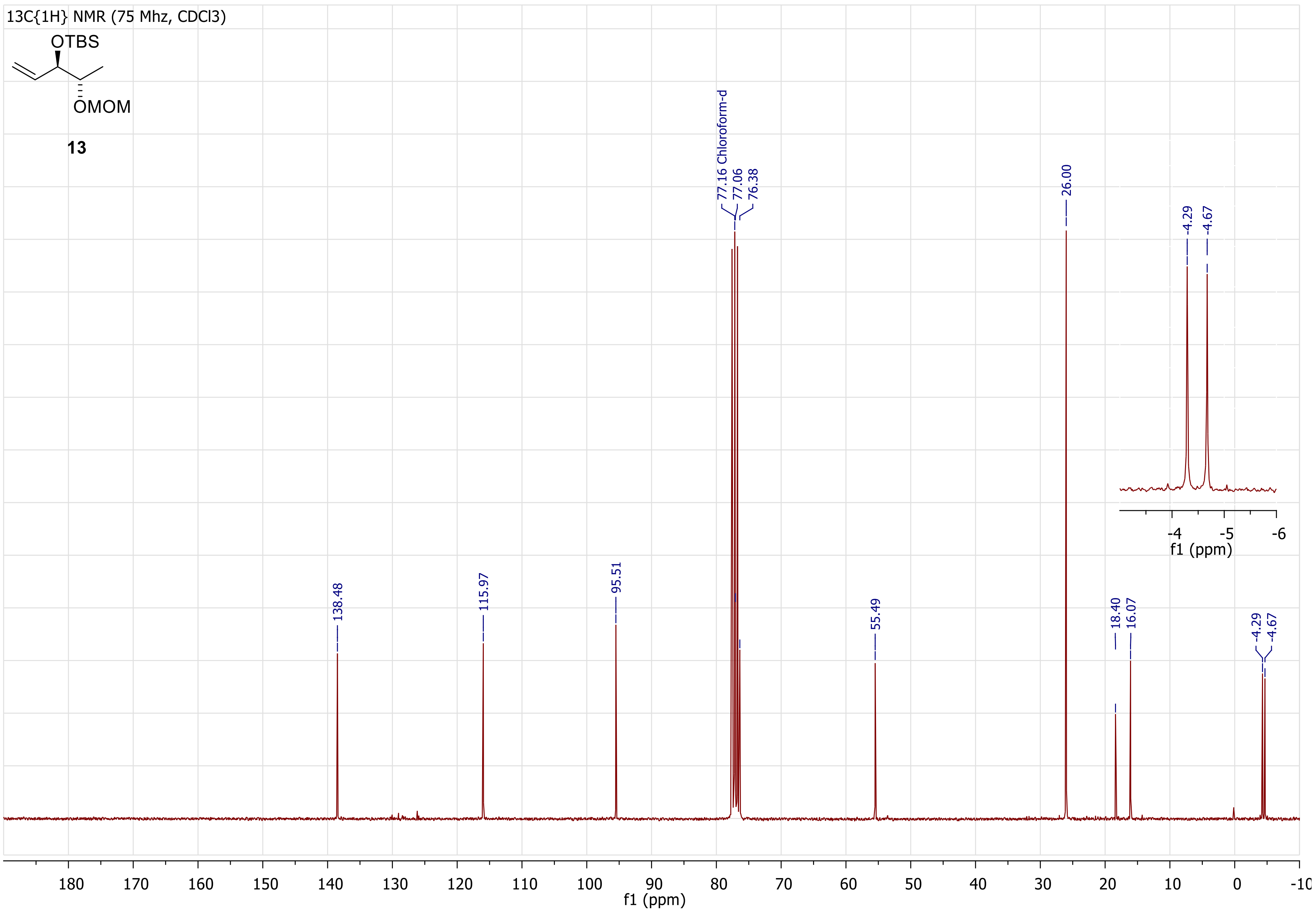

Figure S19. ^{13}C NMR spectrum of **13**.

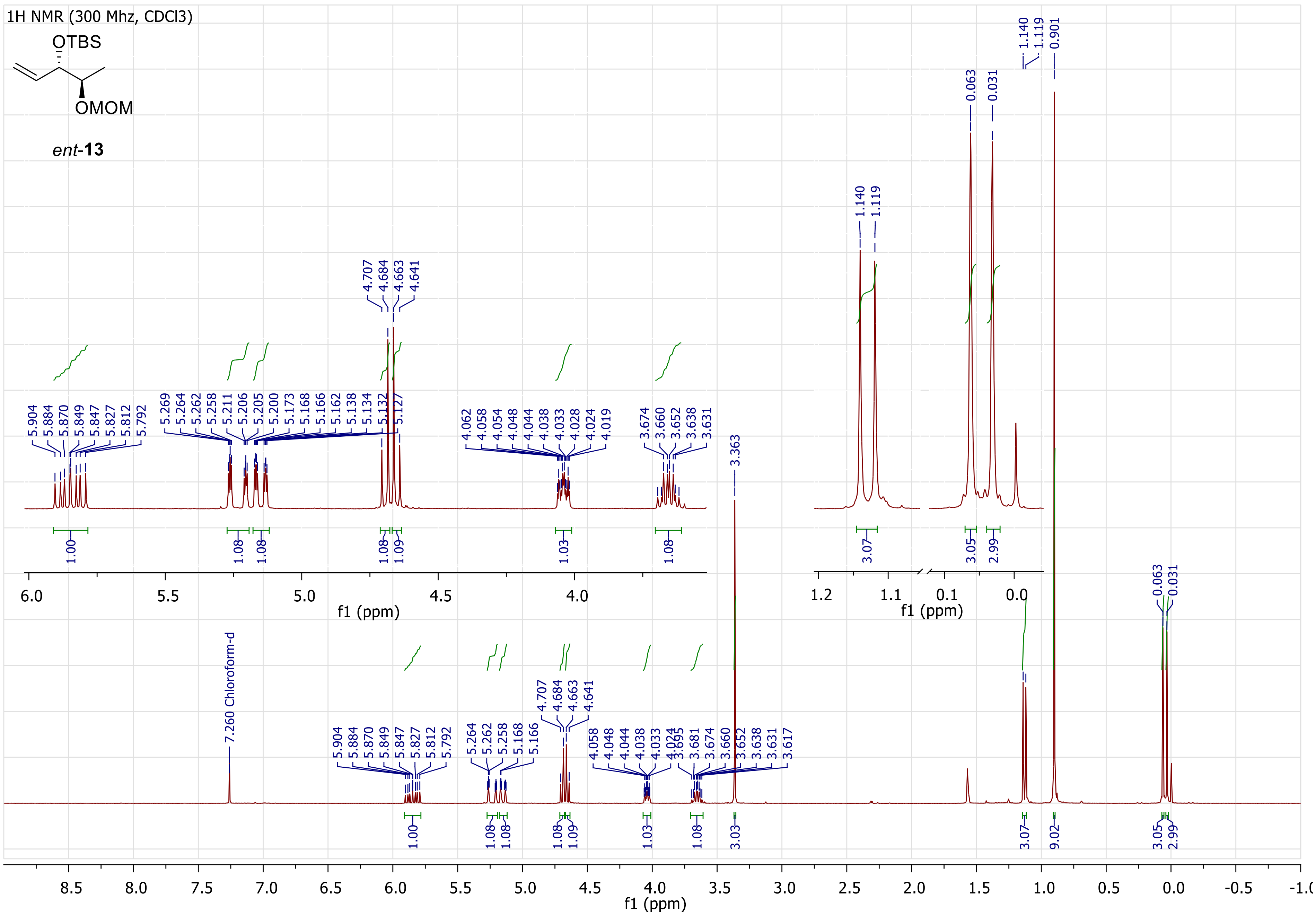

Figure S20. ¹H NMR spectrum of *ent*-**13**.

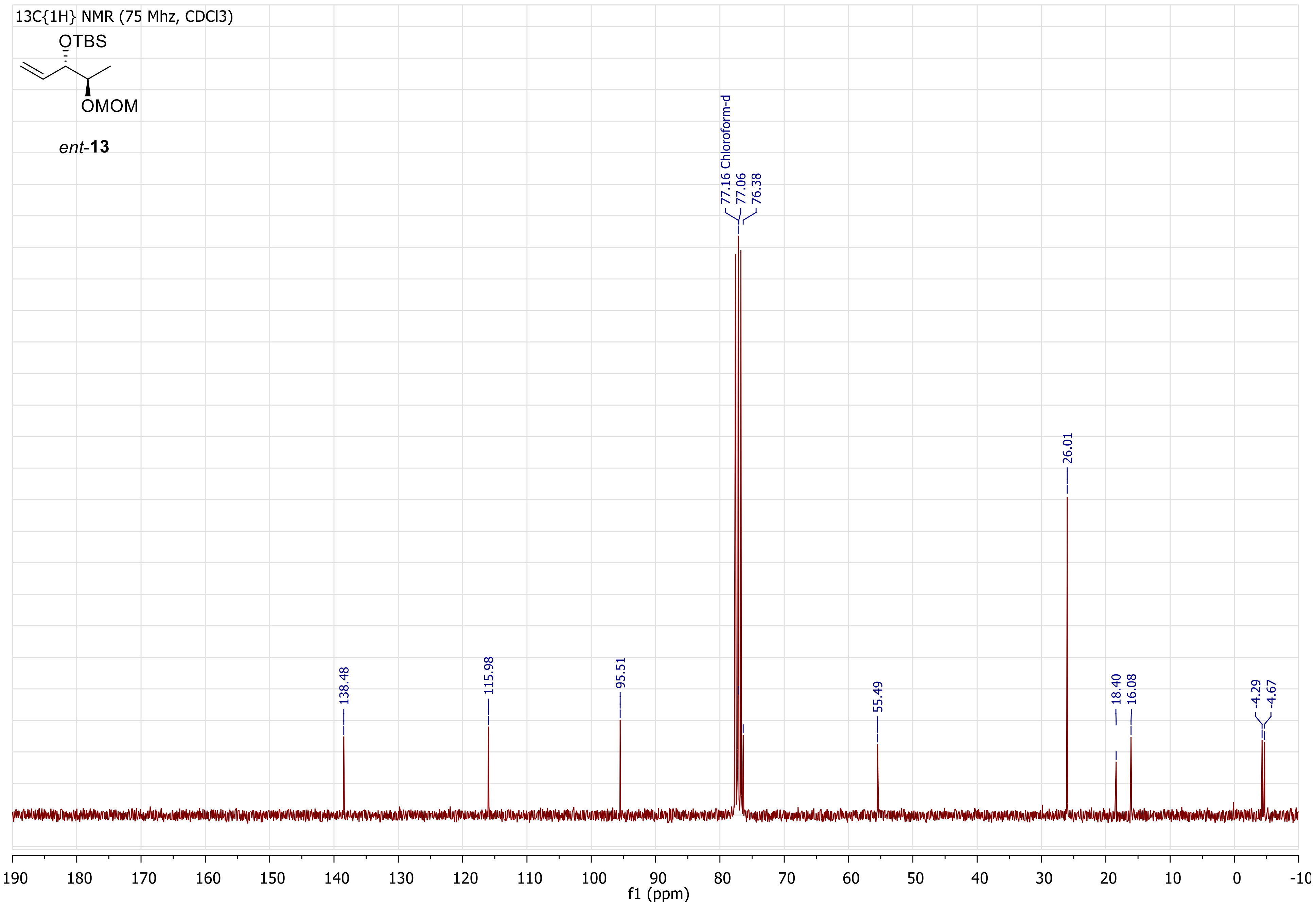

Figure S21. ^{13}C NMR spectrum of *ent*-**13**.

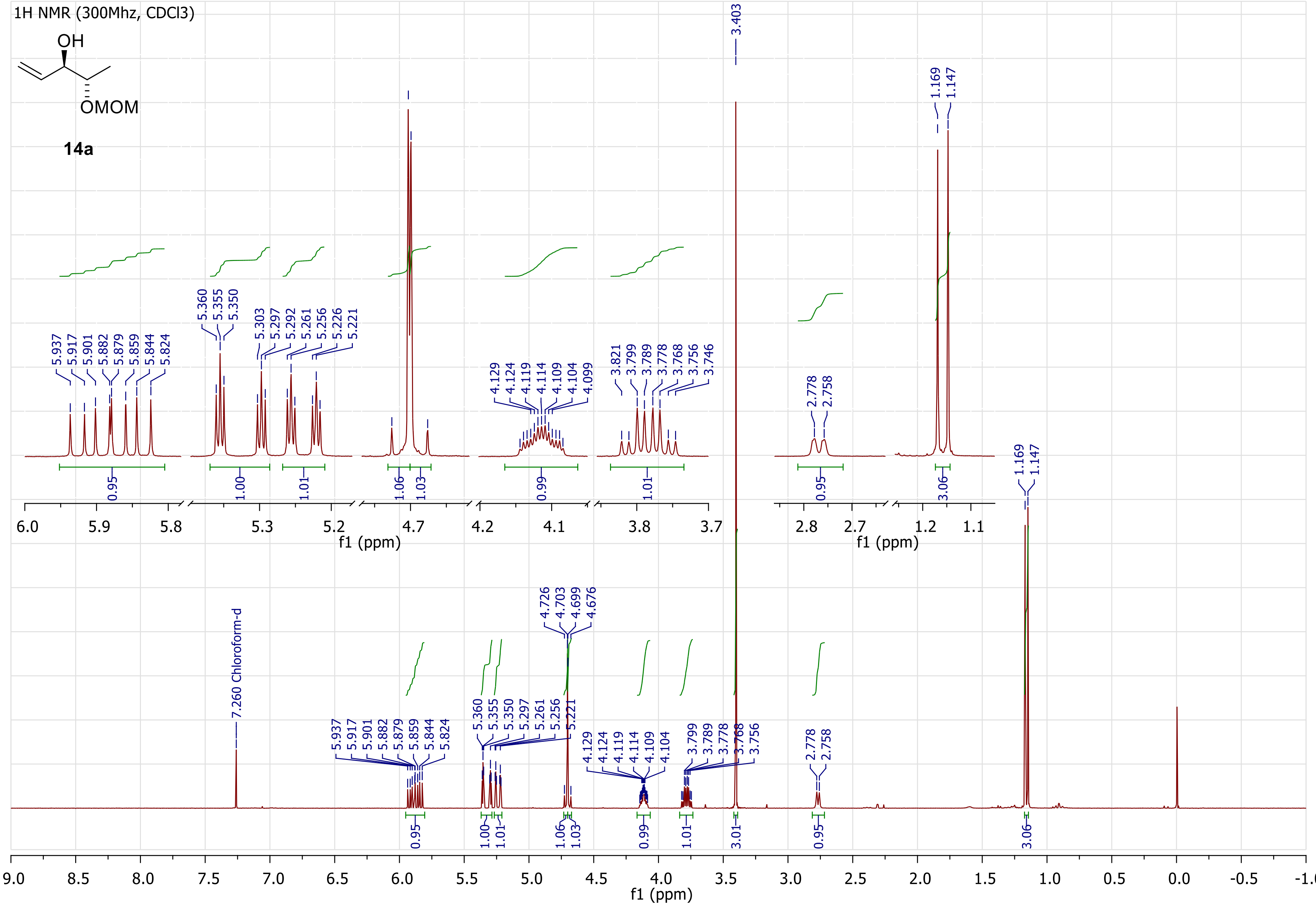

Figure S22. ^{1}H NMR spectrum of **14a**.

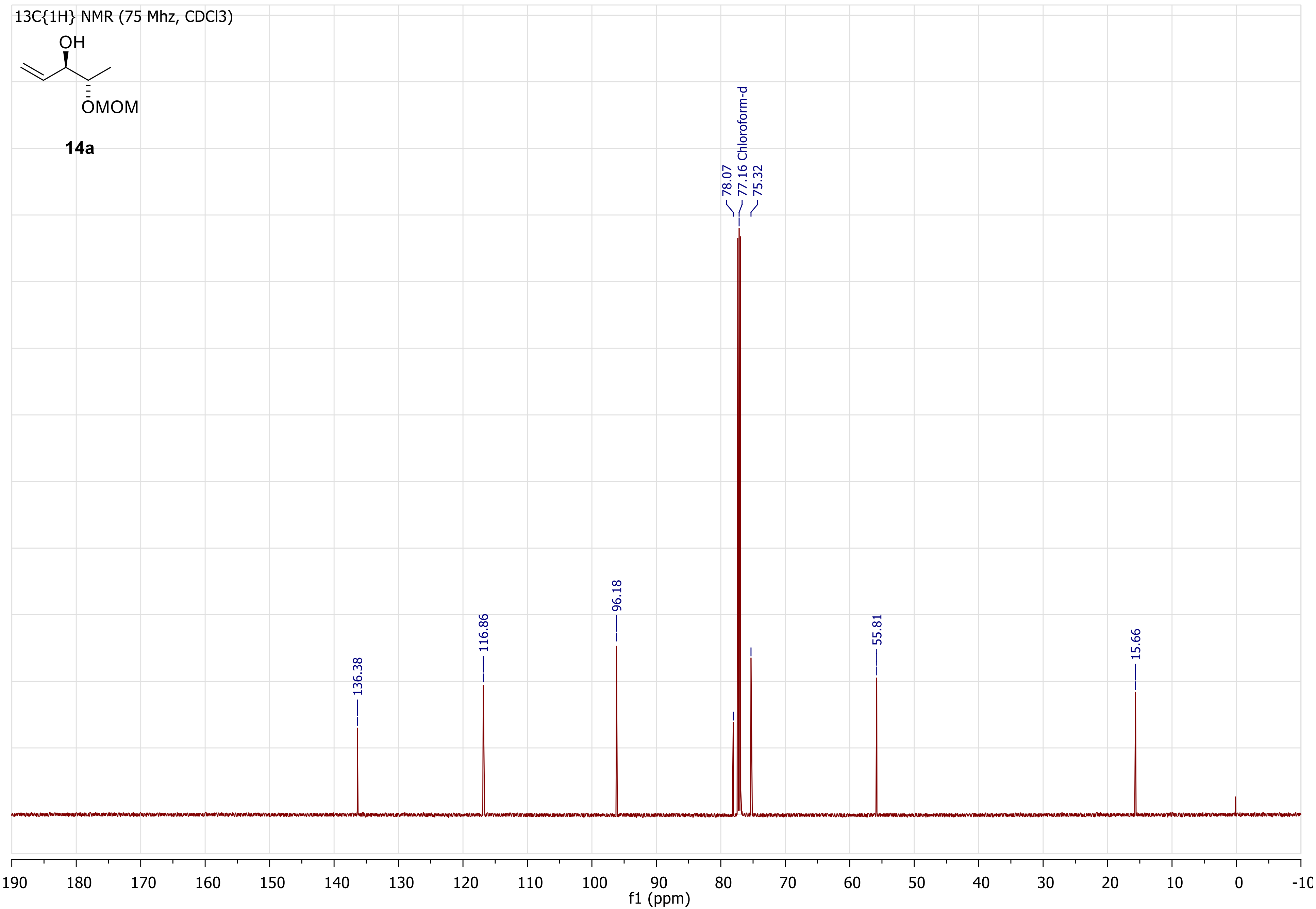

Figure S23. ^{13}C NMR spectrum of **14a**.

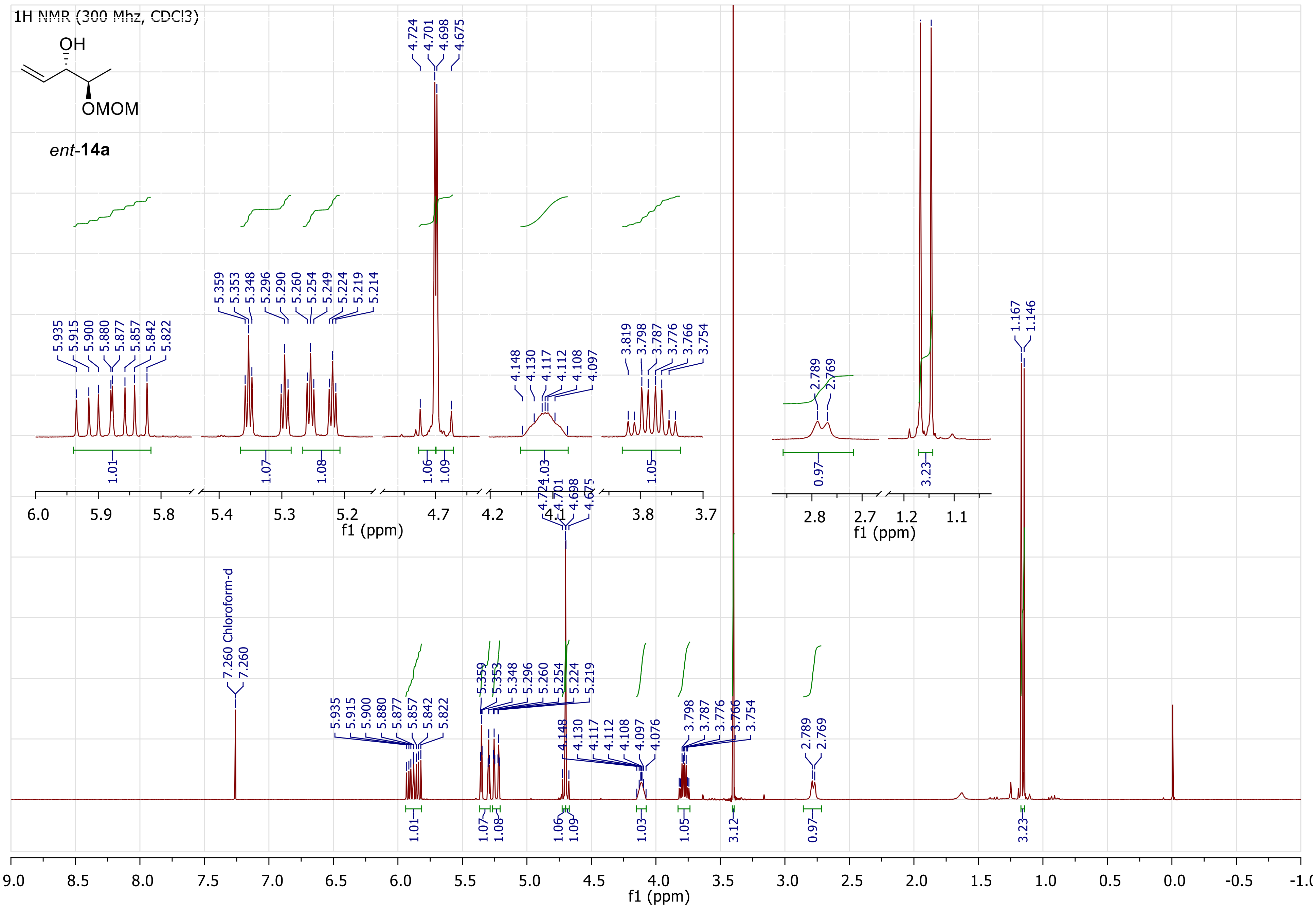

Figure S24. ^{1}H NMR spectrum of *ent*-**14a**.

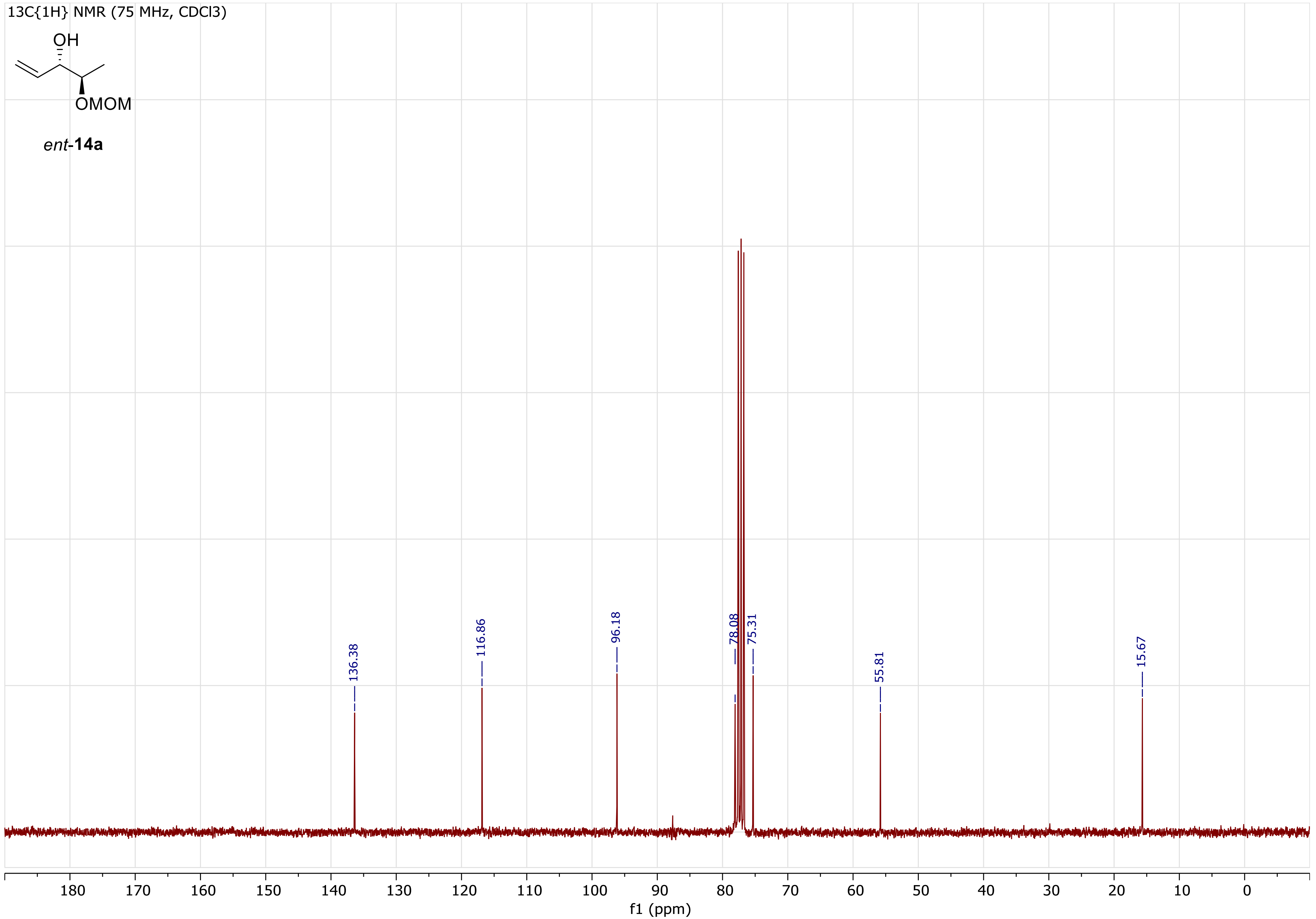

Figure S25. ^{1}H NMR spectrum of *ent*-**14a**.

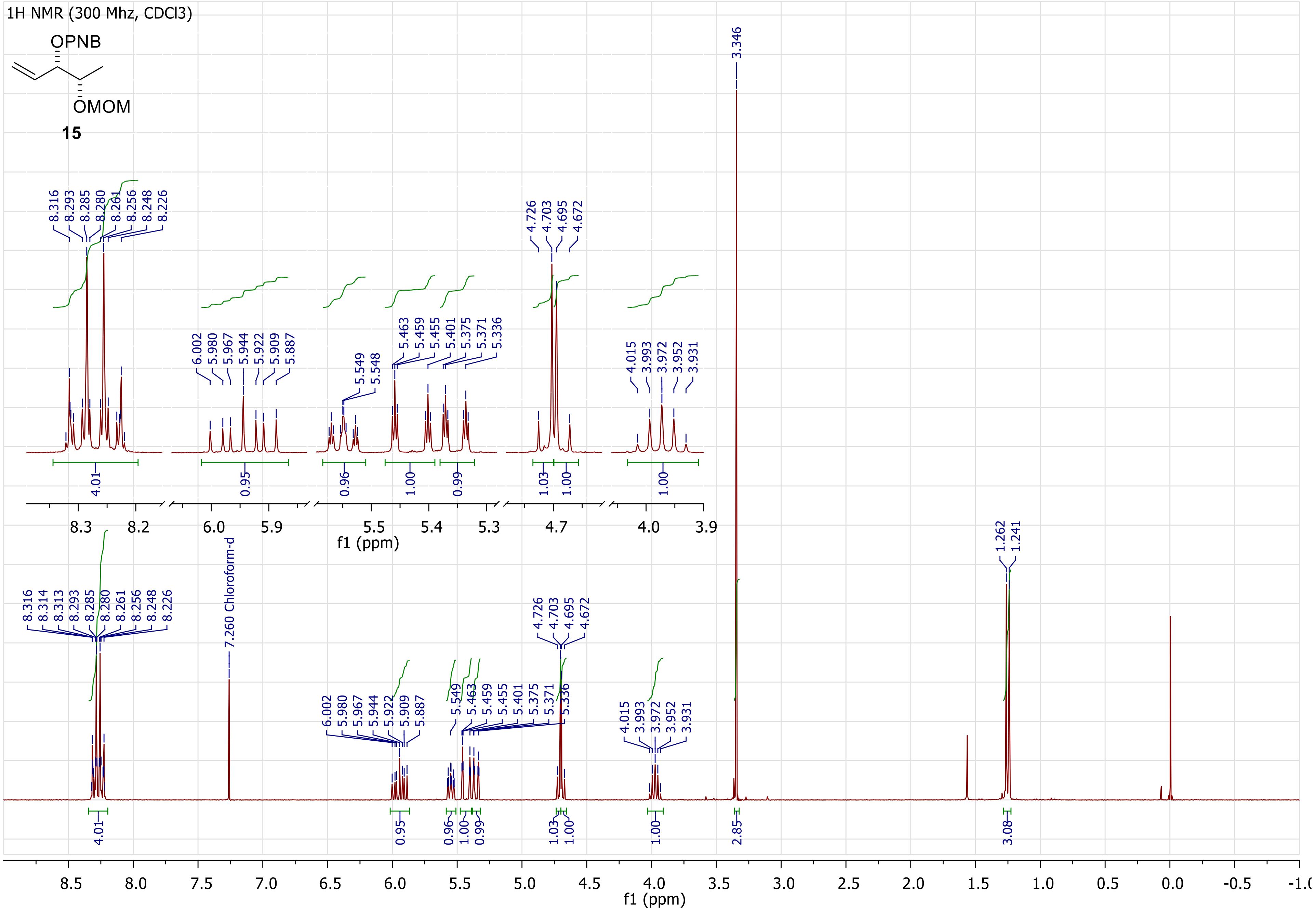

Figure S26. 1H NMR spectrum of **15**.

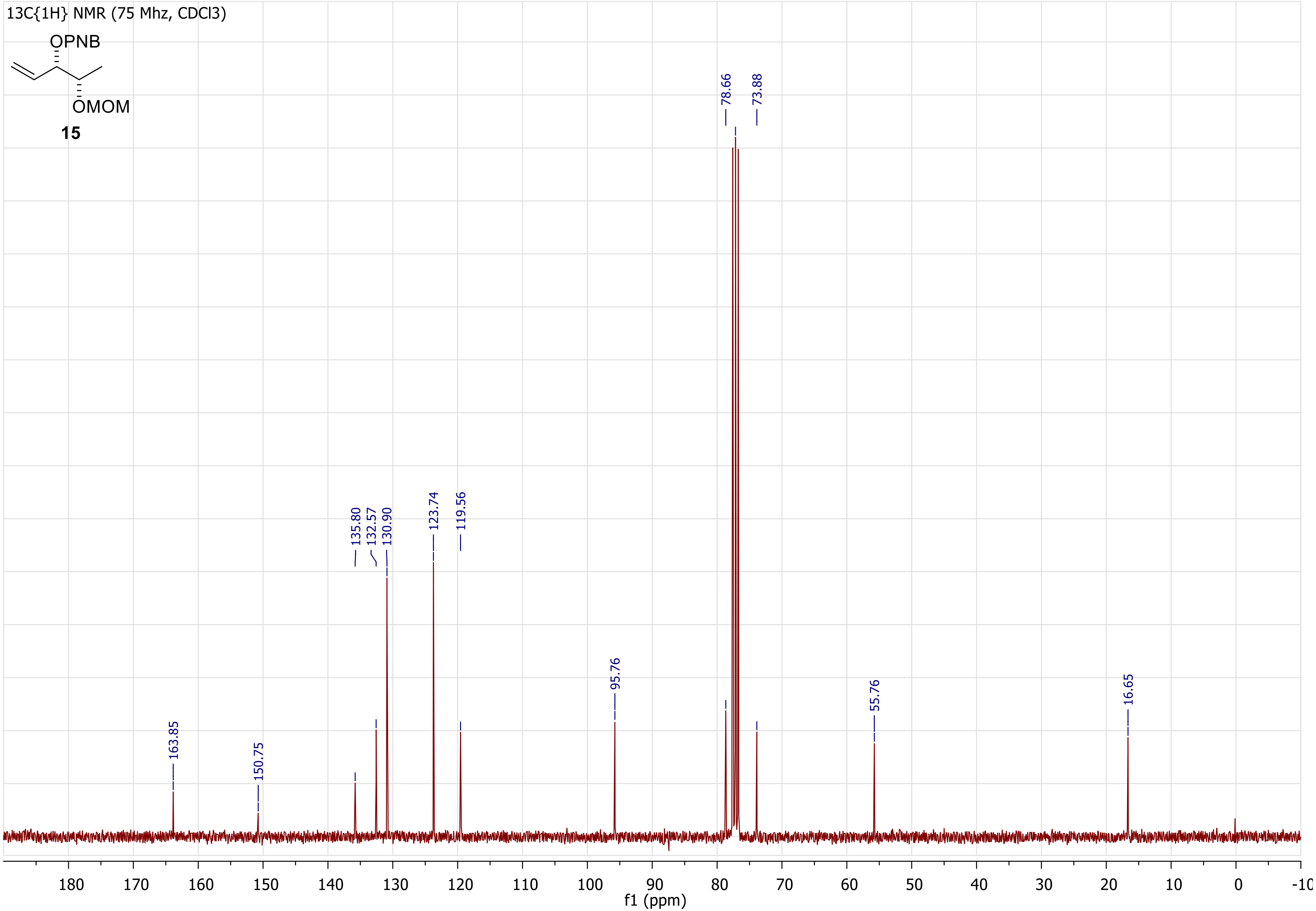

Figure S27. ^{13}C NMR spectrum of **15**.

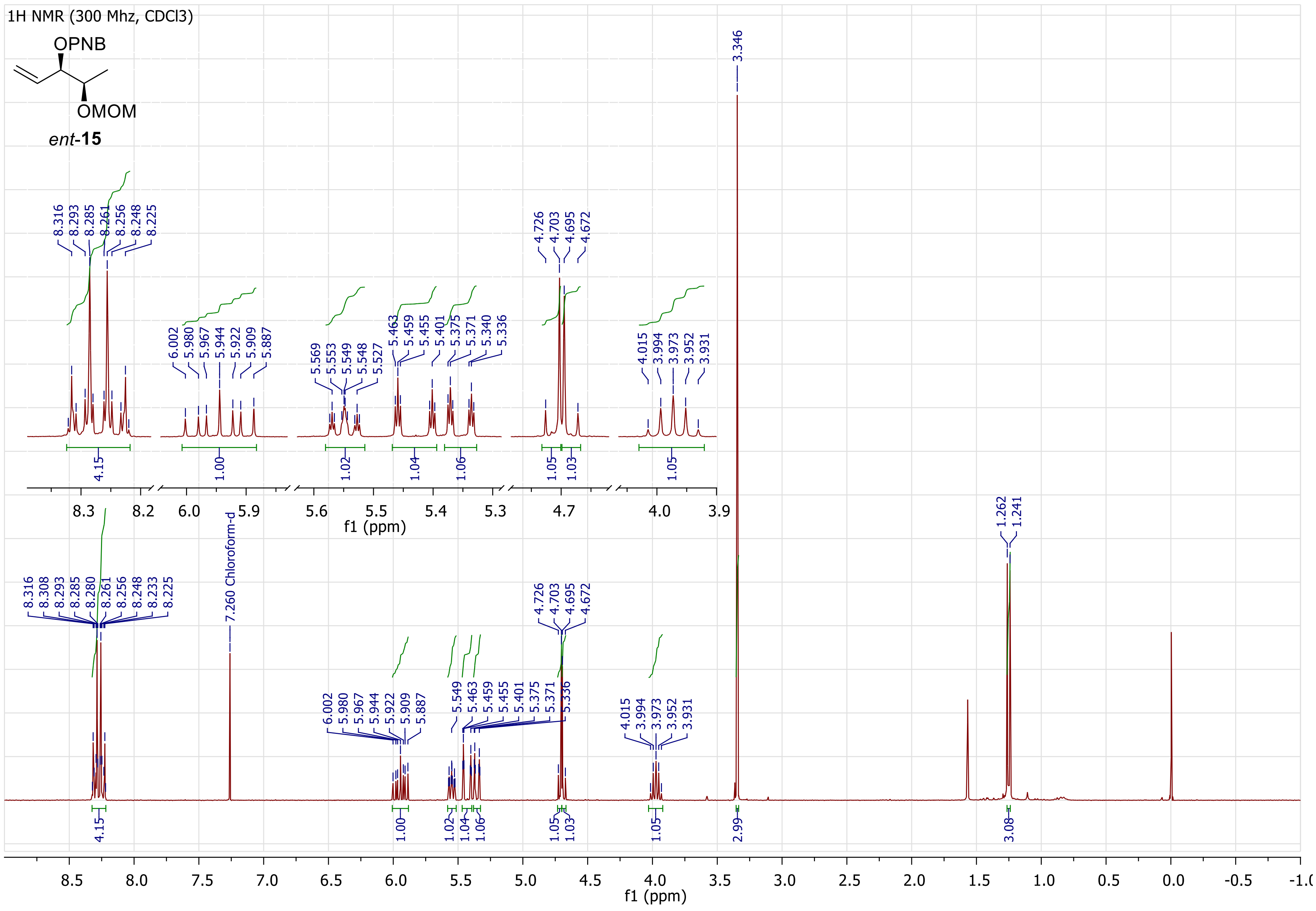

Figure S28. ^{1}H NMR spectrum of *ent*-**15**.

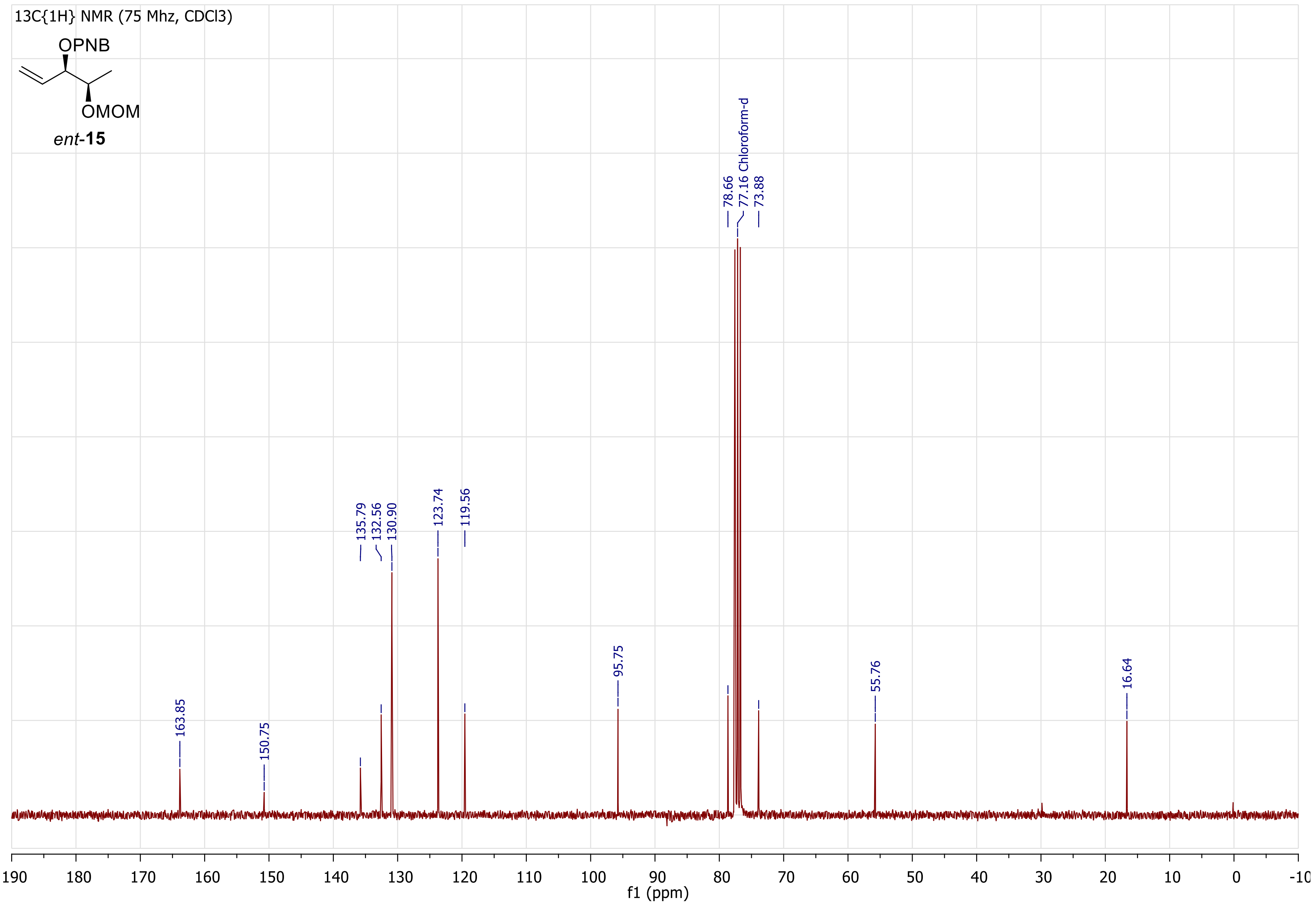

Figure S29. ^{13}C NMR spectrum of *ent*-**15**.

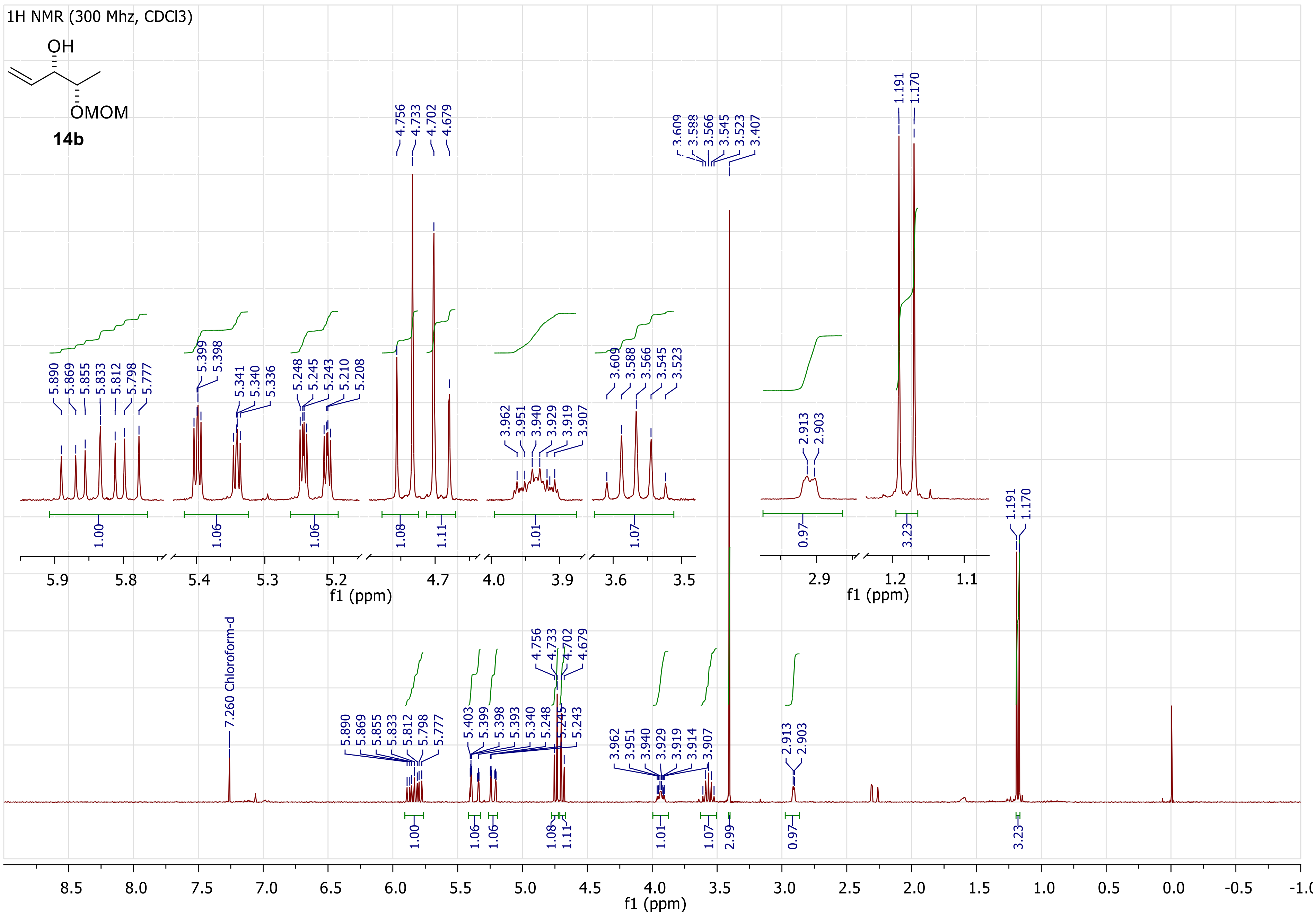

Figure S30. ^{1}H NMR spectrum of **14b**.

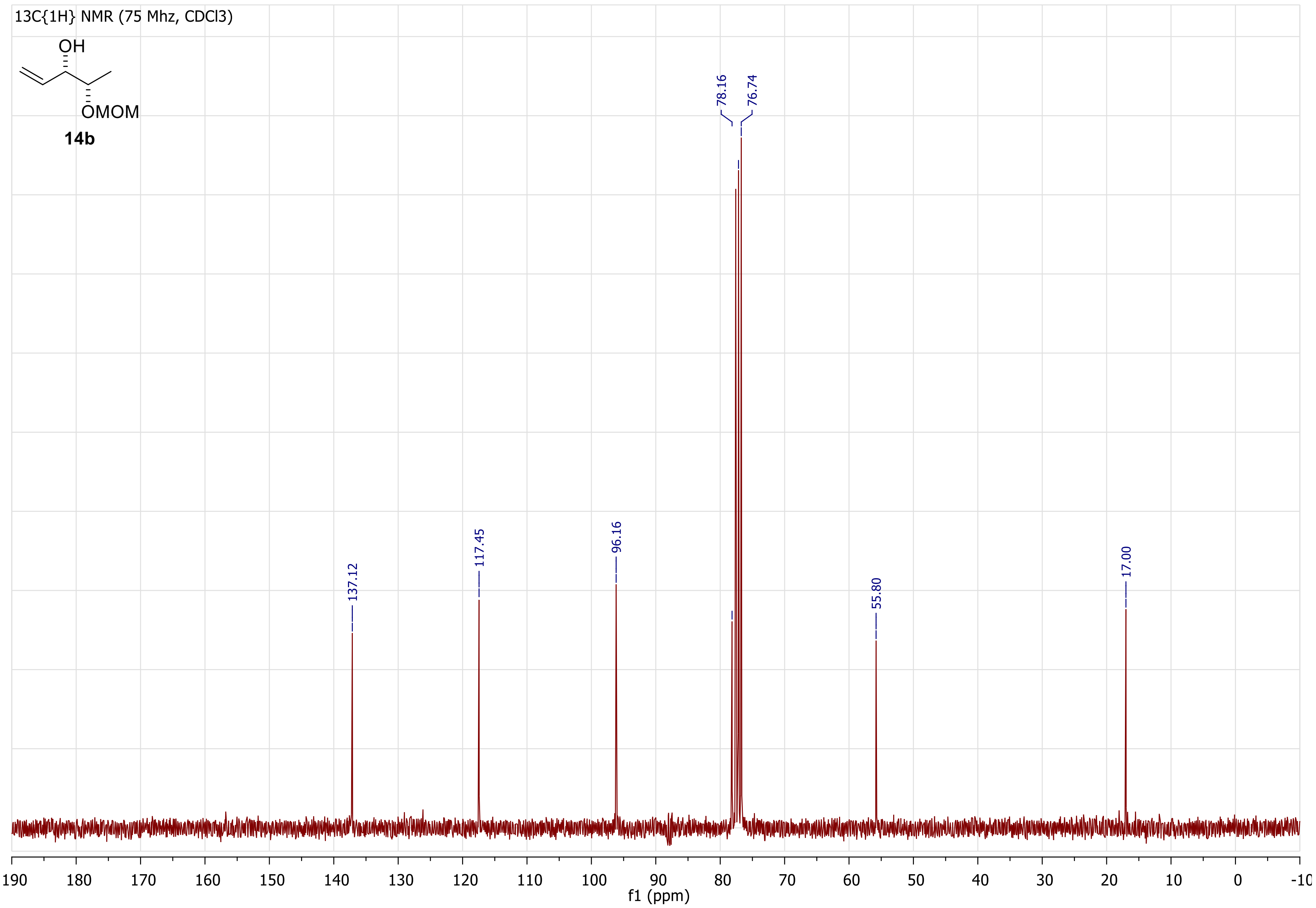

Figure S31. ^{13}C NMR spectrum of **14b**.

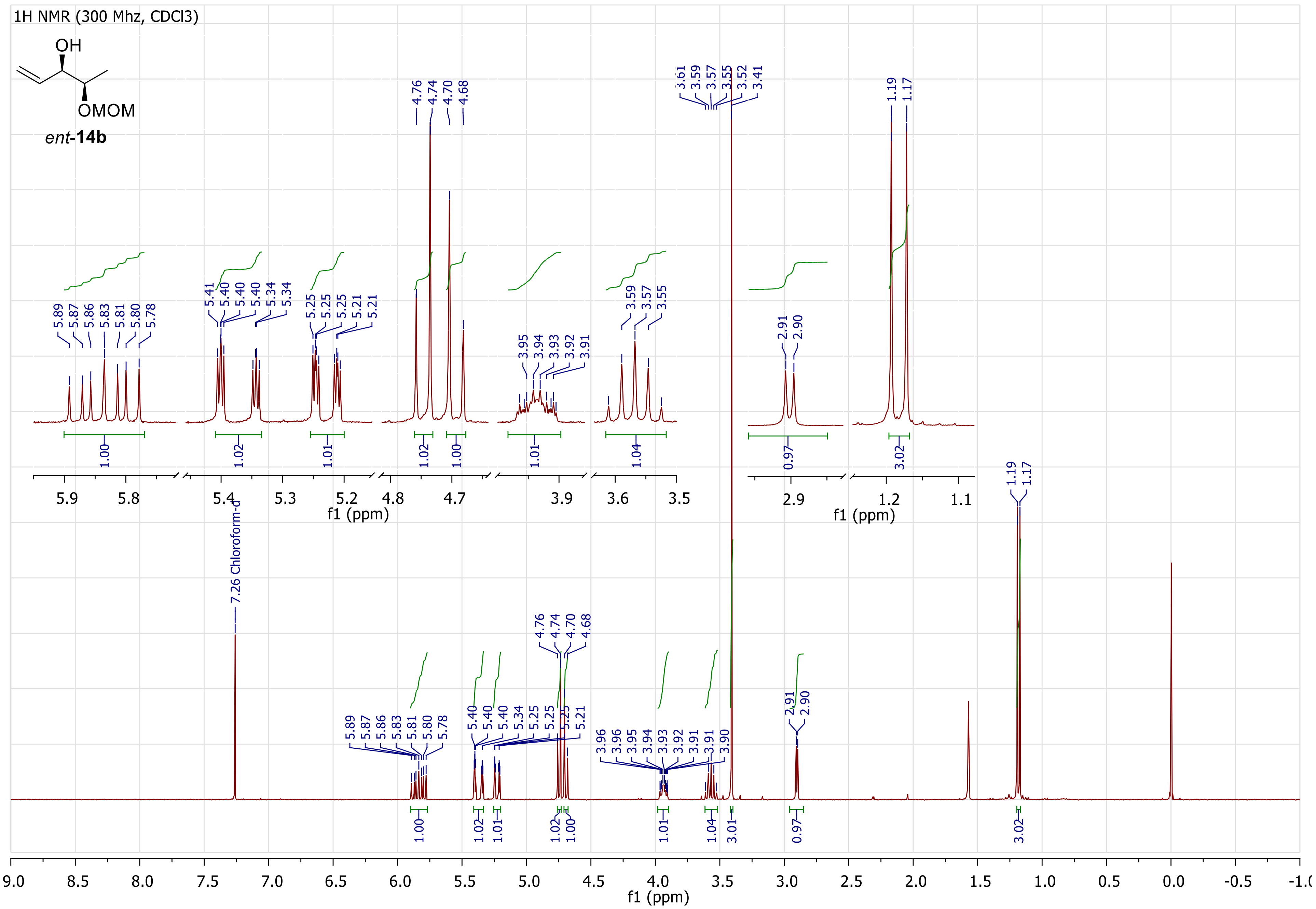

Figure S32. ^{1}H NMR spectrum of *ent*-**14b**.

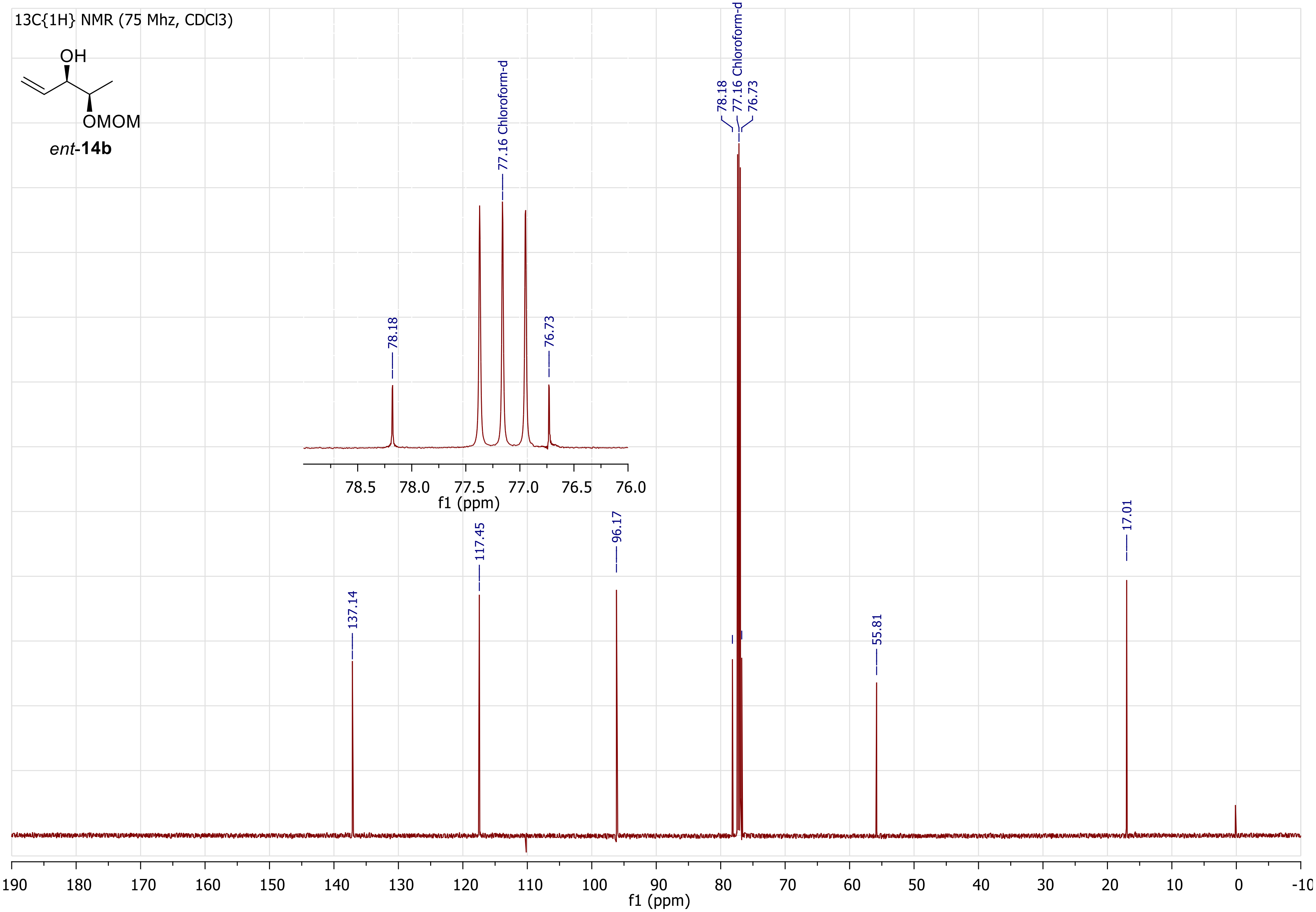

Figure S33. ^{13}C NMR spectrum of *ent*-**14b**.

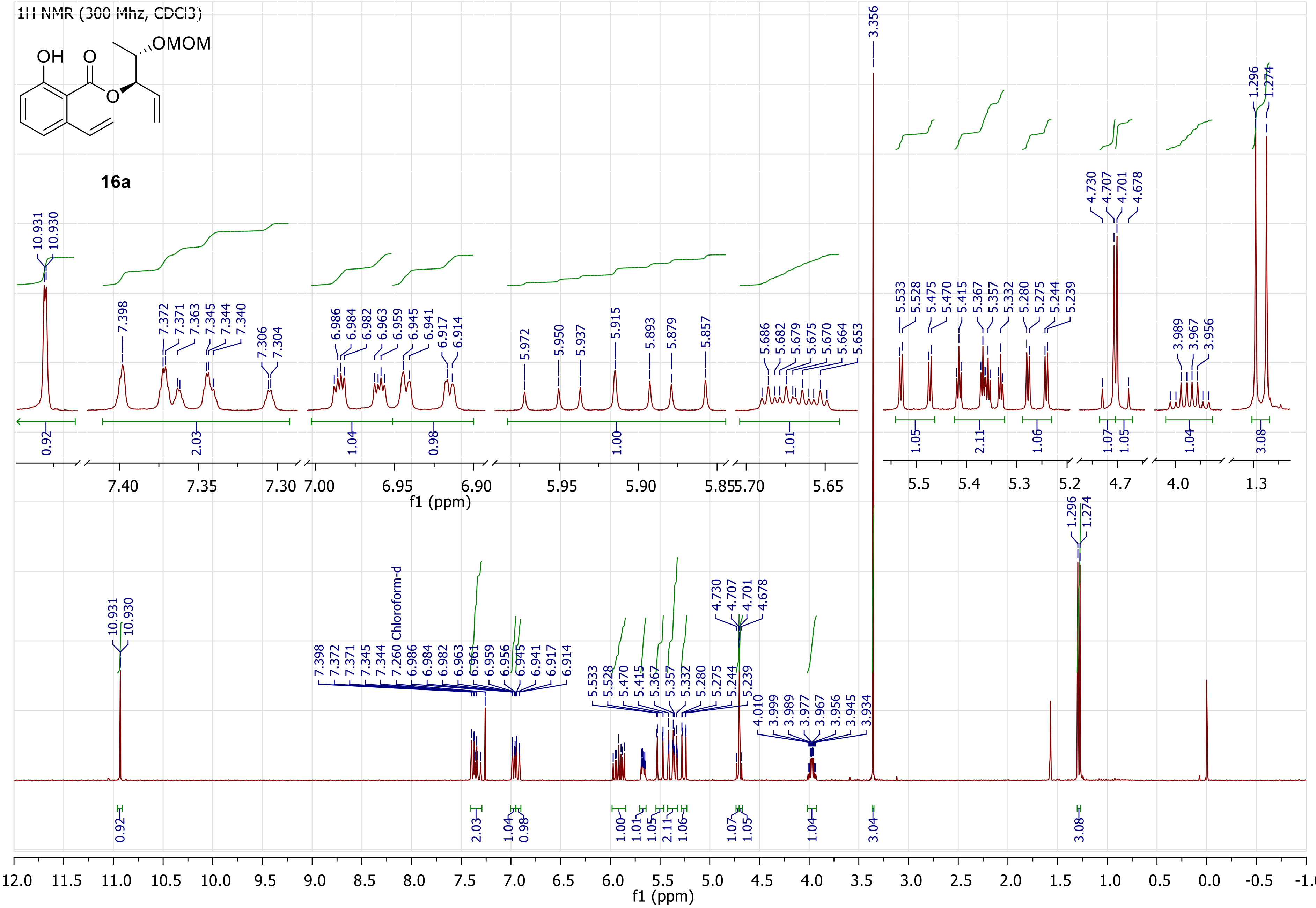

Figure S34. ^{1}H NMR spectrum of **16a**.

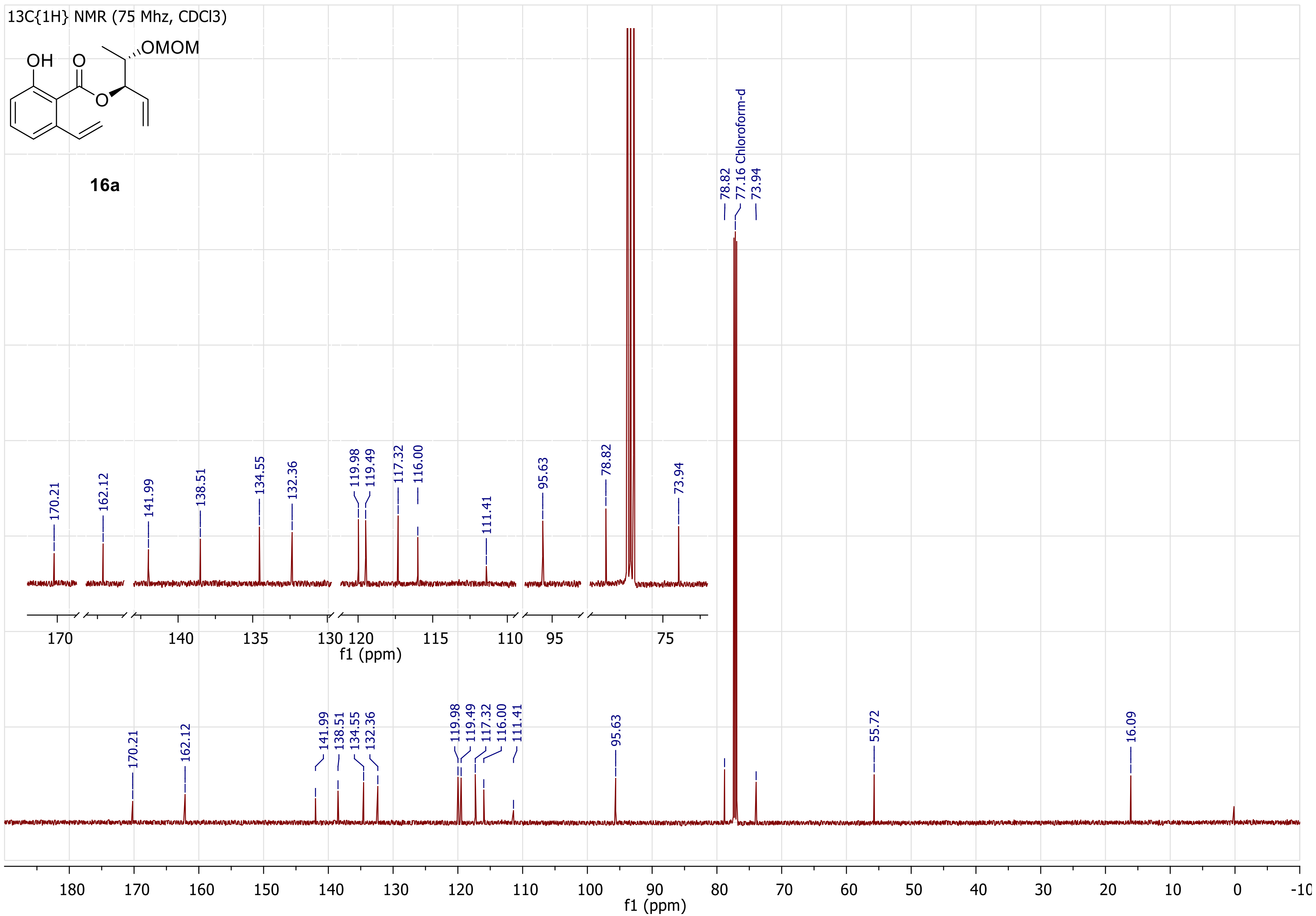

Figure S35. ^{13}C NMR spectrum of **16a**.

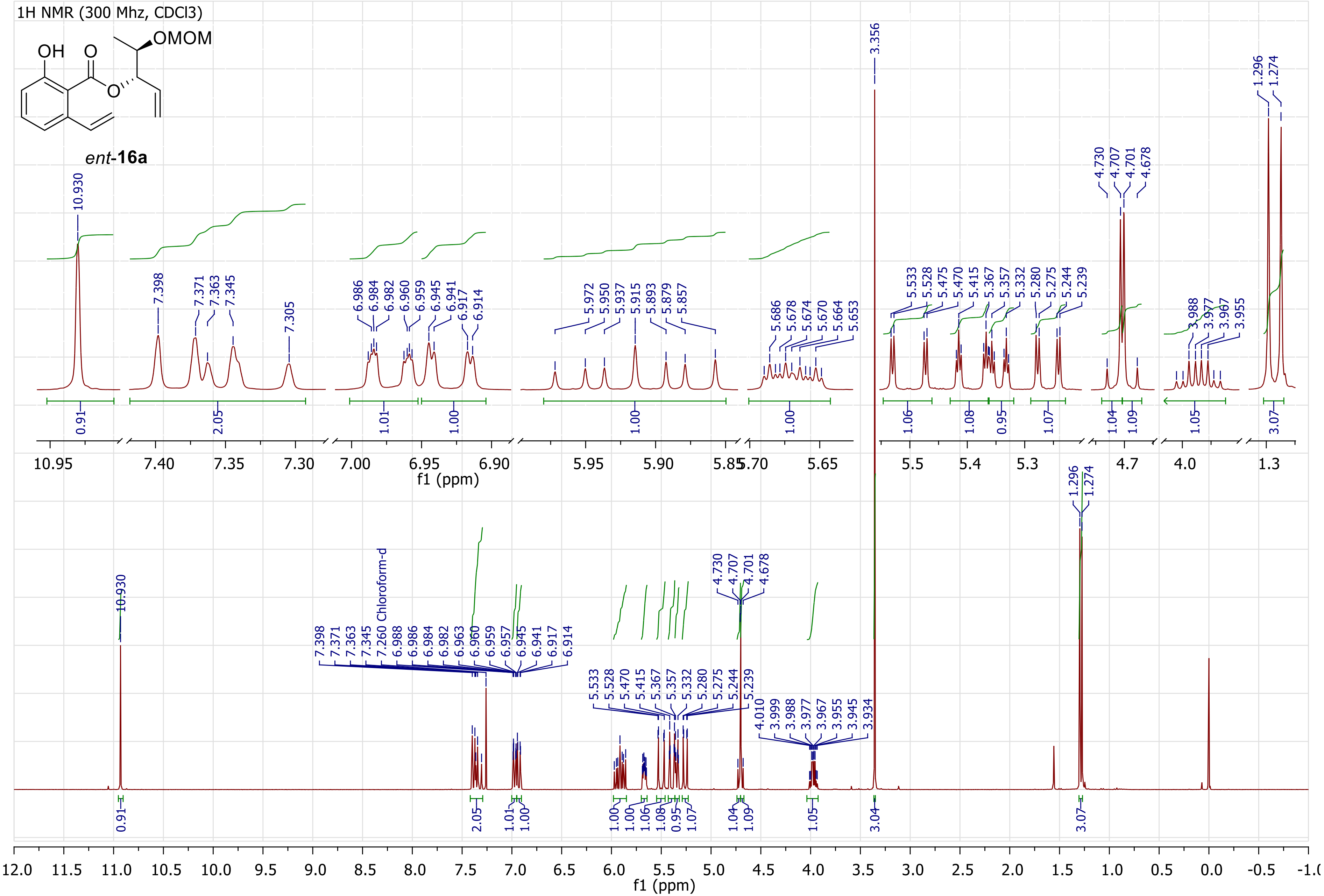

Figure S36. ¹H NMR spectrum of *ent*-**16a**.

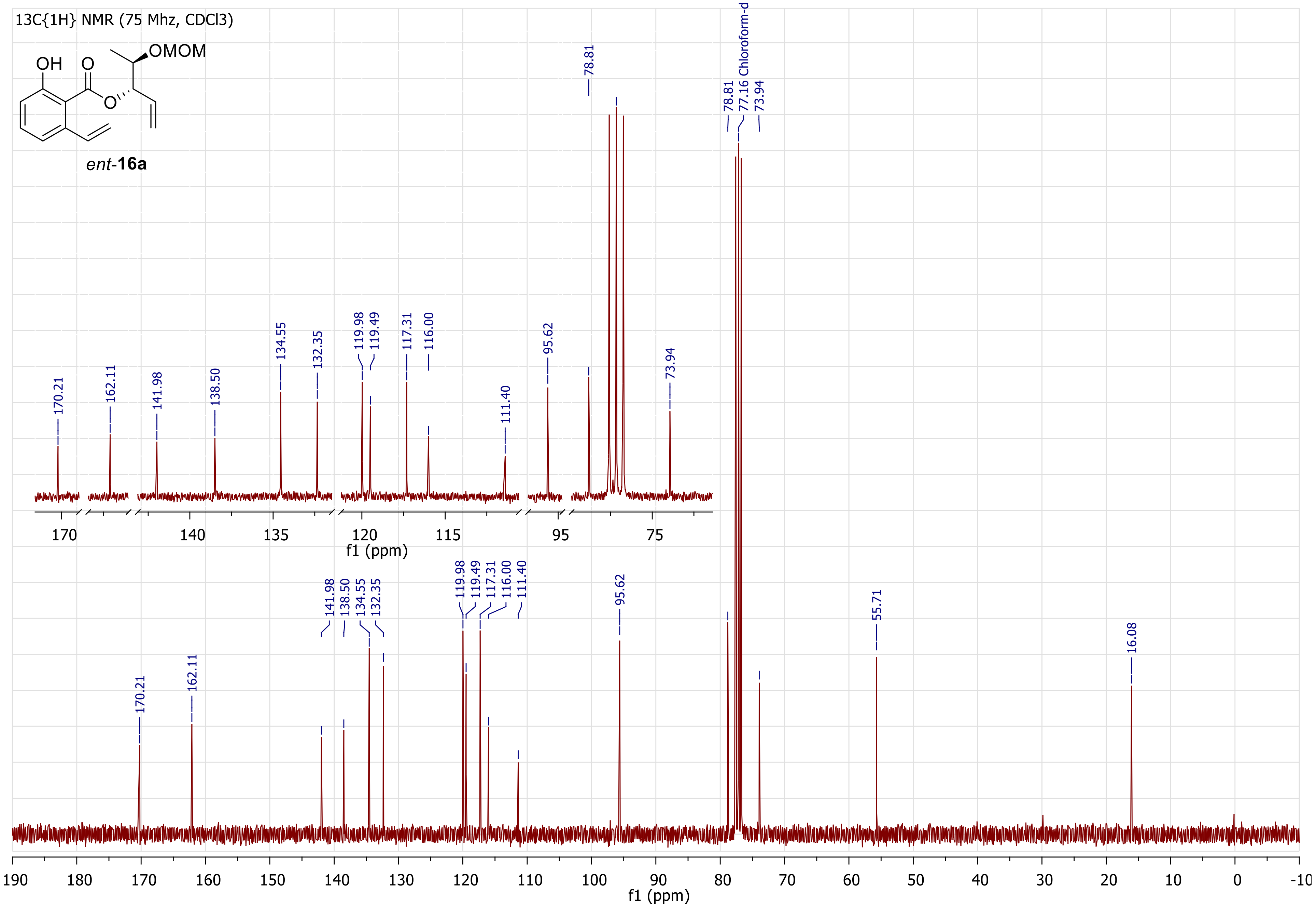

Figure S37. ^{13}C NMR spectrum of *ent*-**16a**.

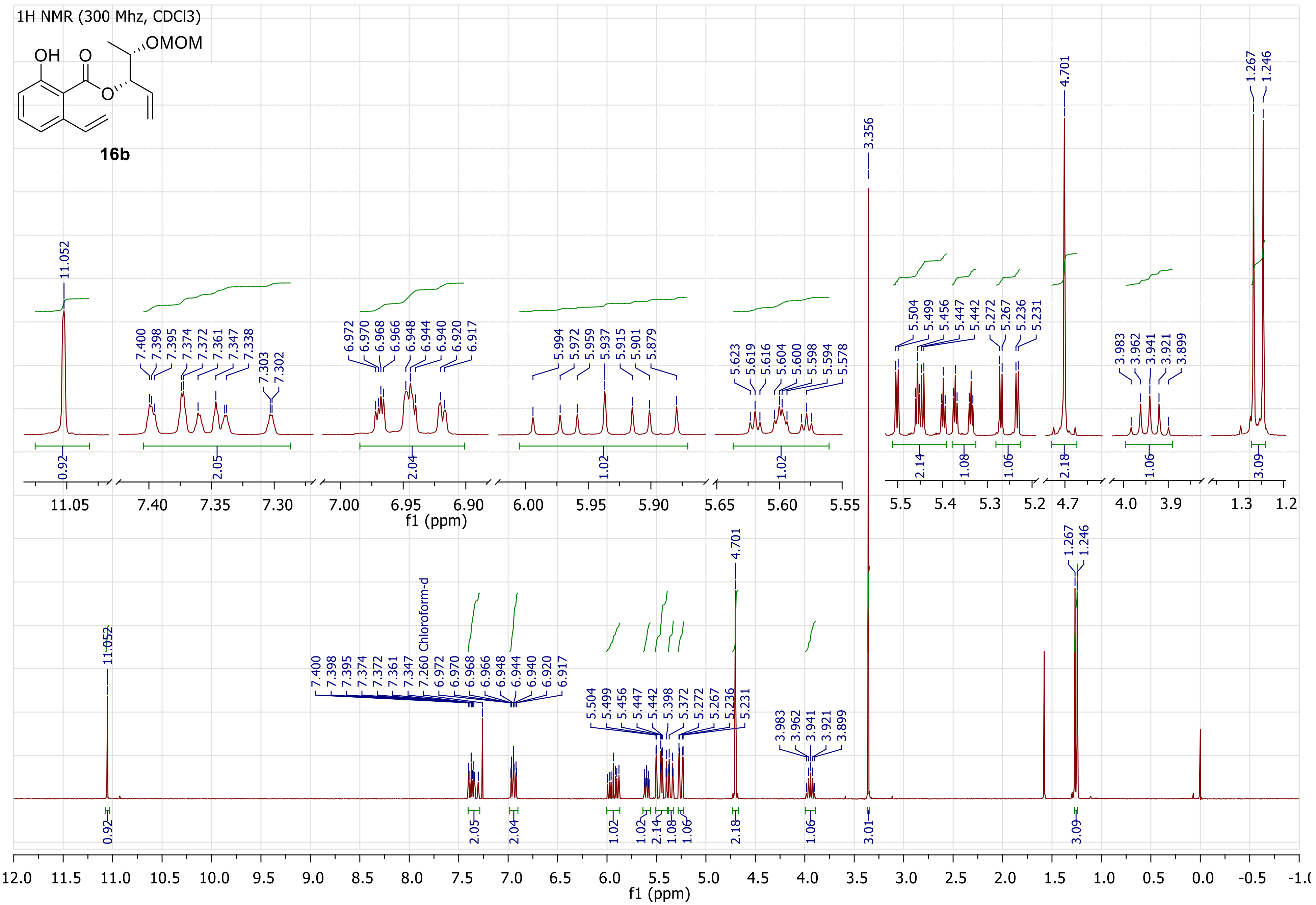

Figure S38. ^{1}H NMR spectrum of **16b**.

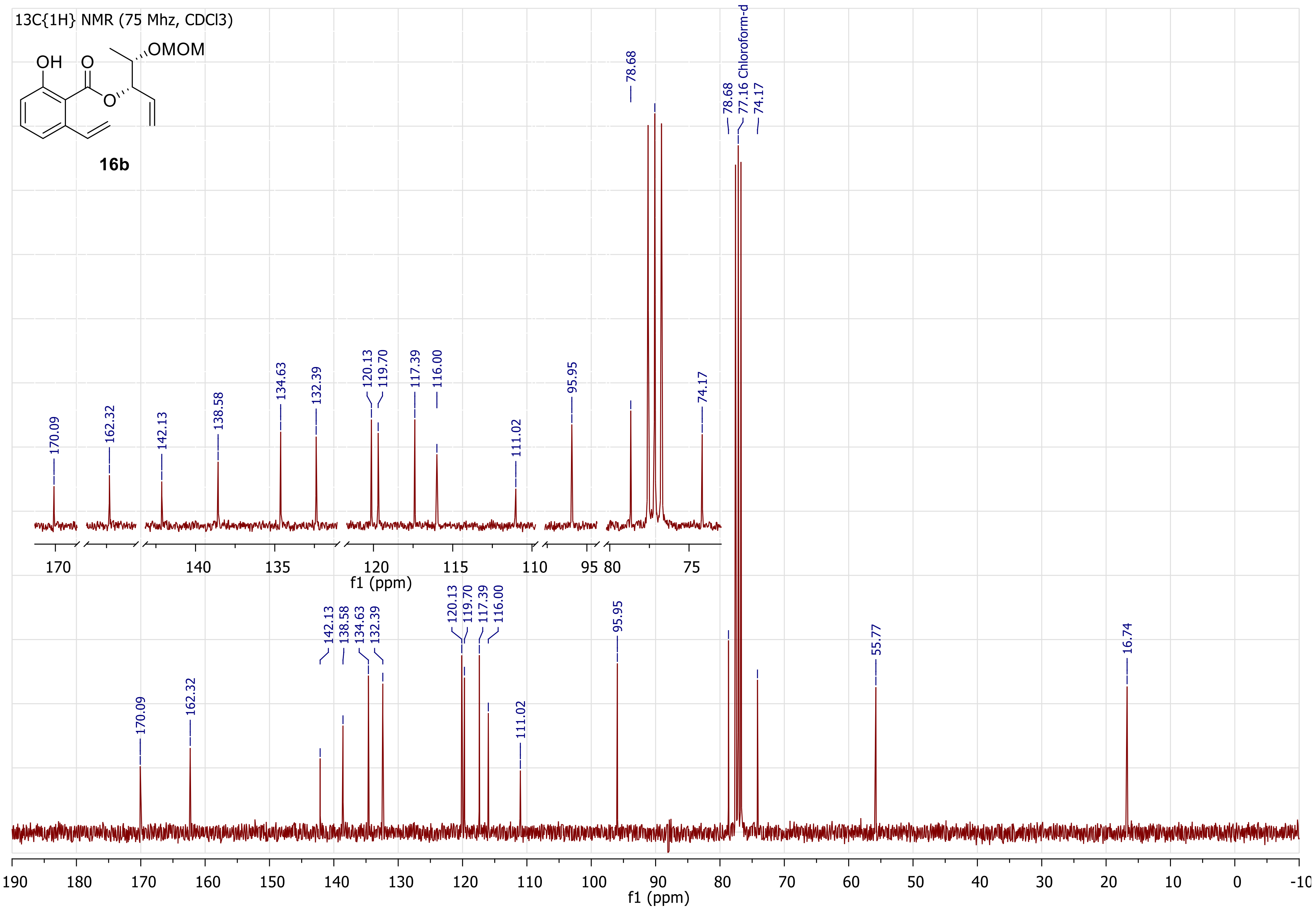

Figure S39. ^{1}H NMR spectrum of **16b**.

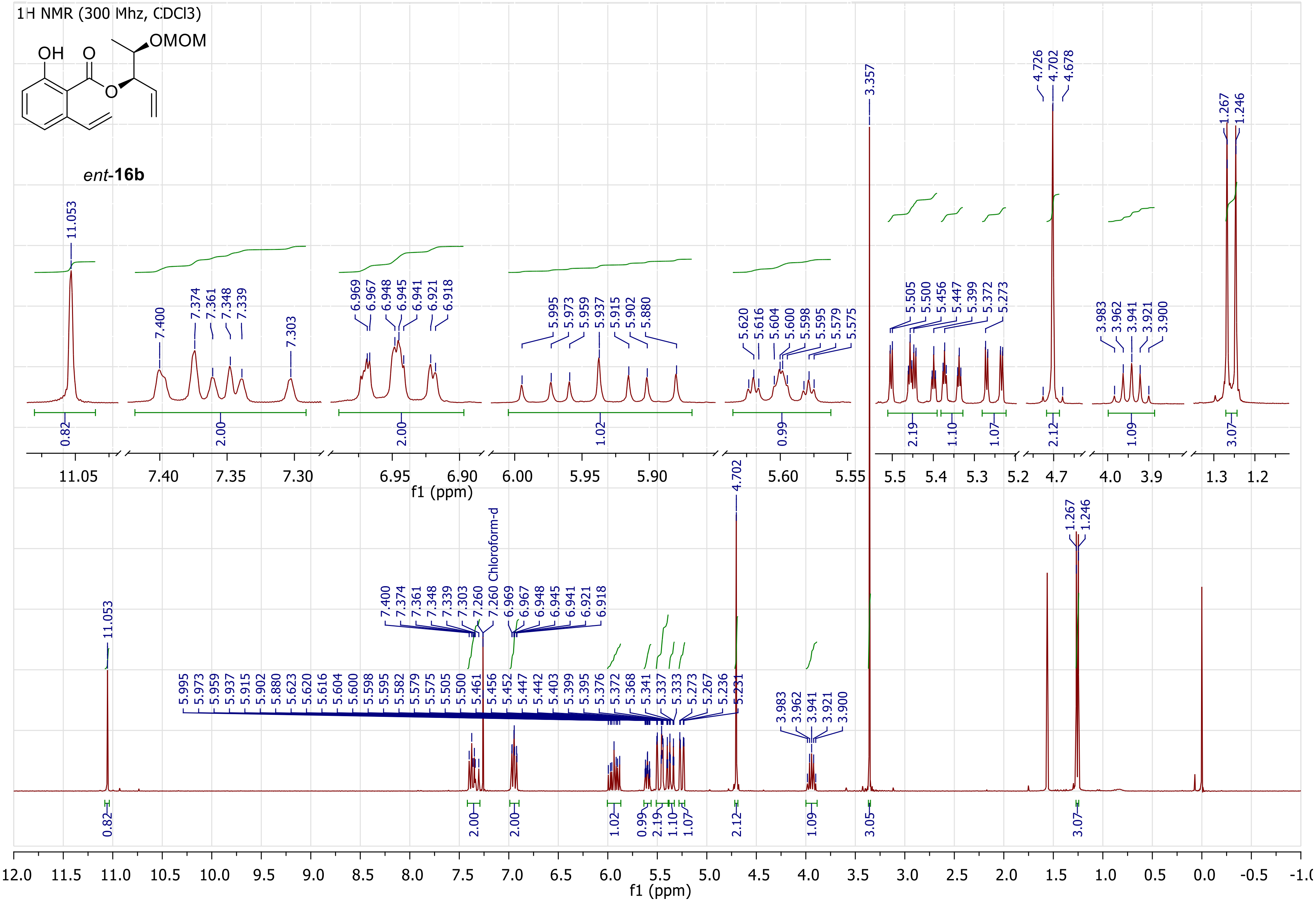

Figure S40. ^{1}H NMR spectrum of *ent*-**16b**.

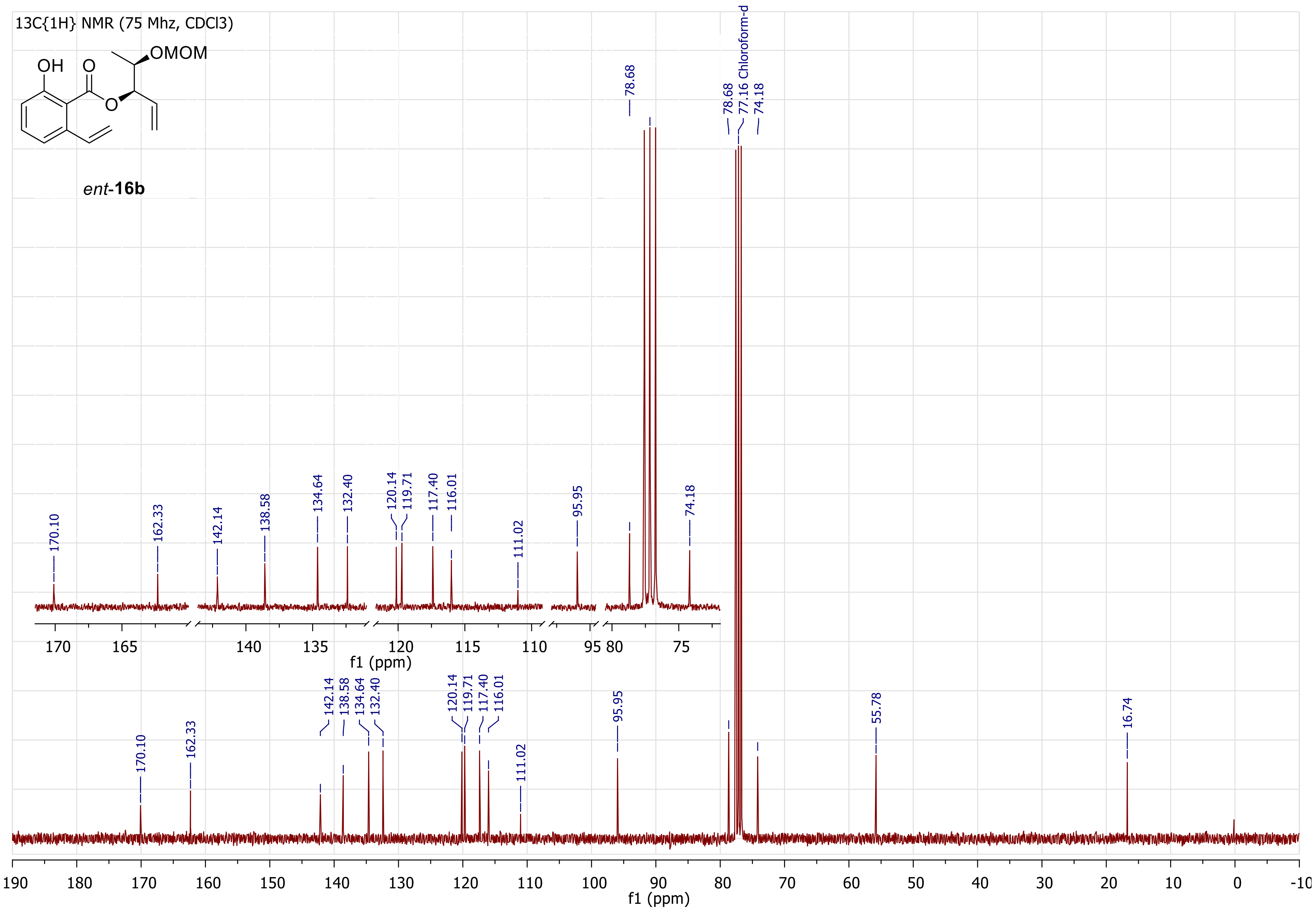

Figure S41. ^{1}H NMR spectrum of *ent*-**16b**.

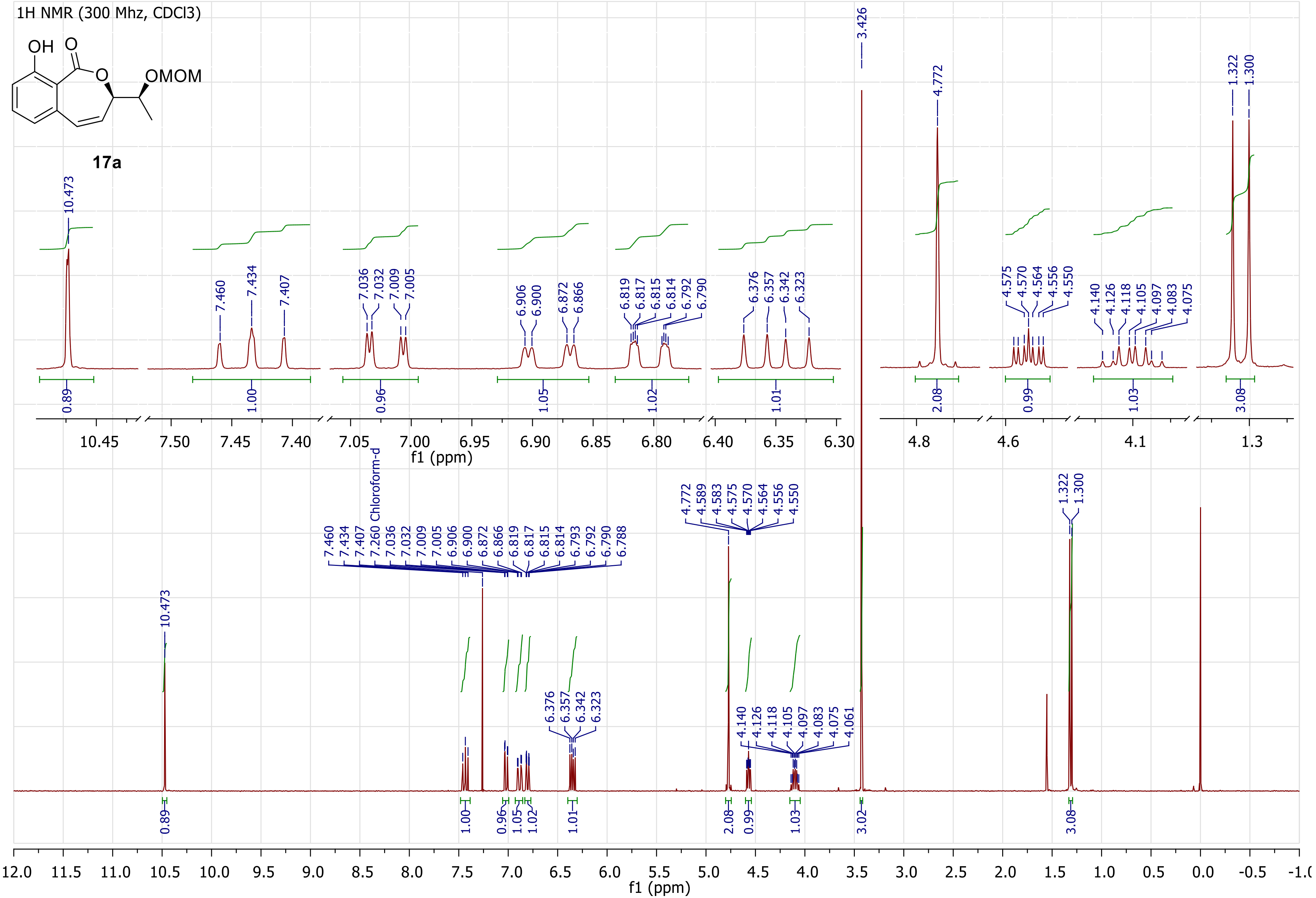

Figure S42. ^{1}H NMR spectrum of **17a**.

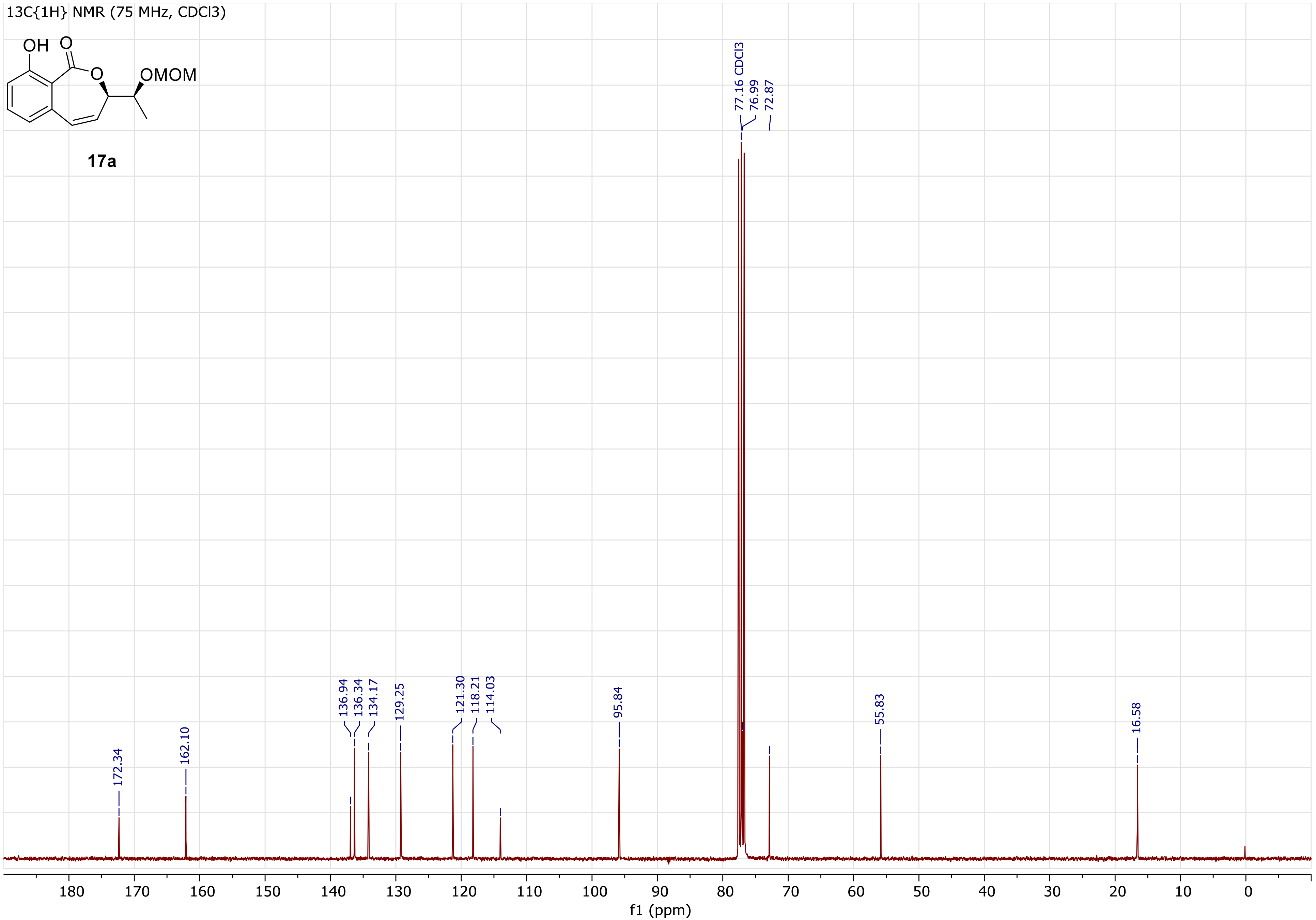

Figure S43. ^{13}C NMR spectrum of **17a**.

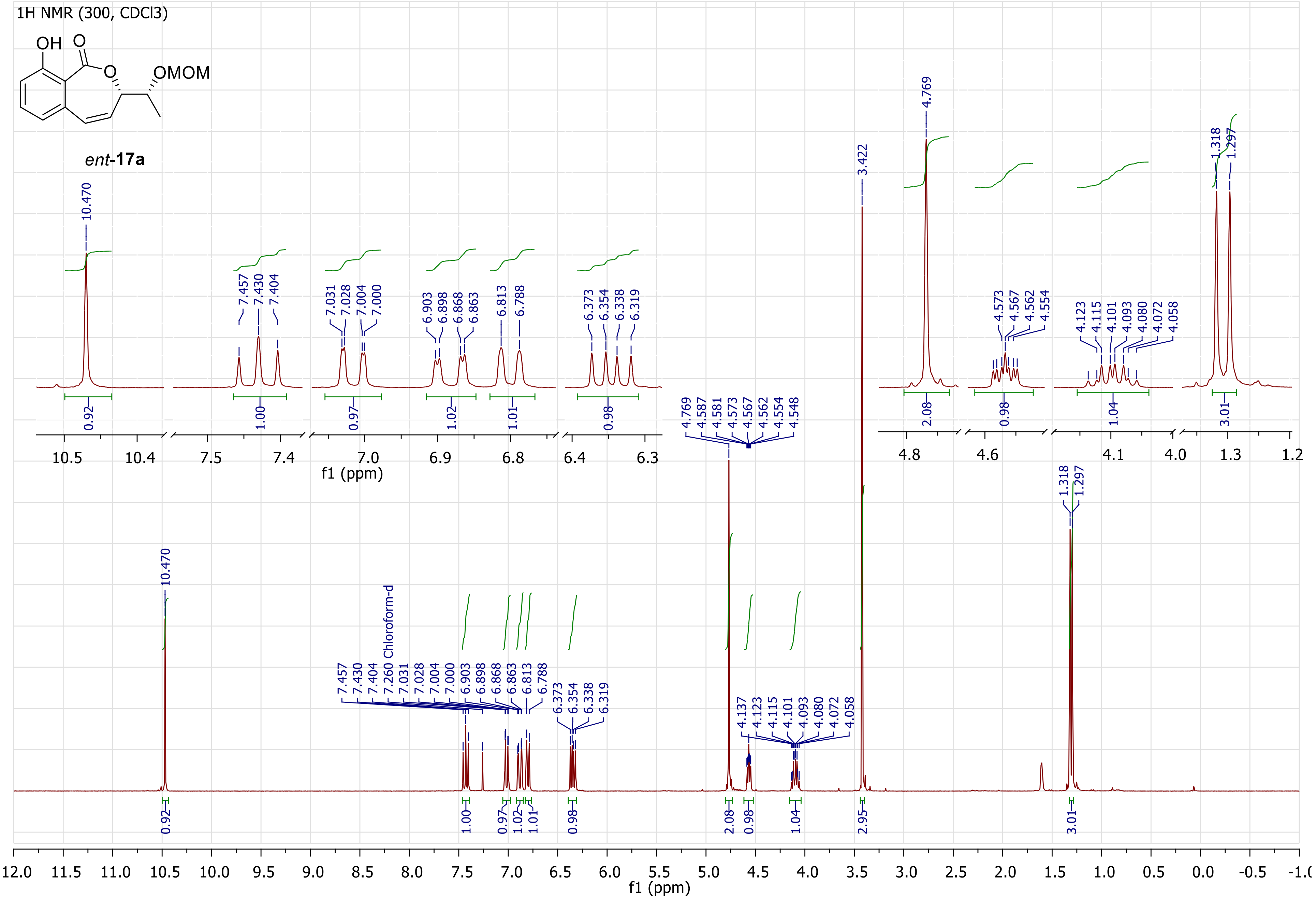

Figure S44. ^{1}H NMR spectrum of *ent*-**17a**.

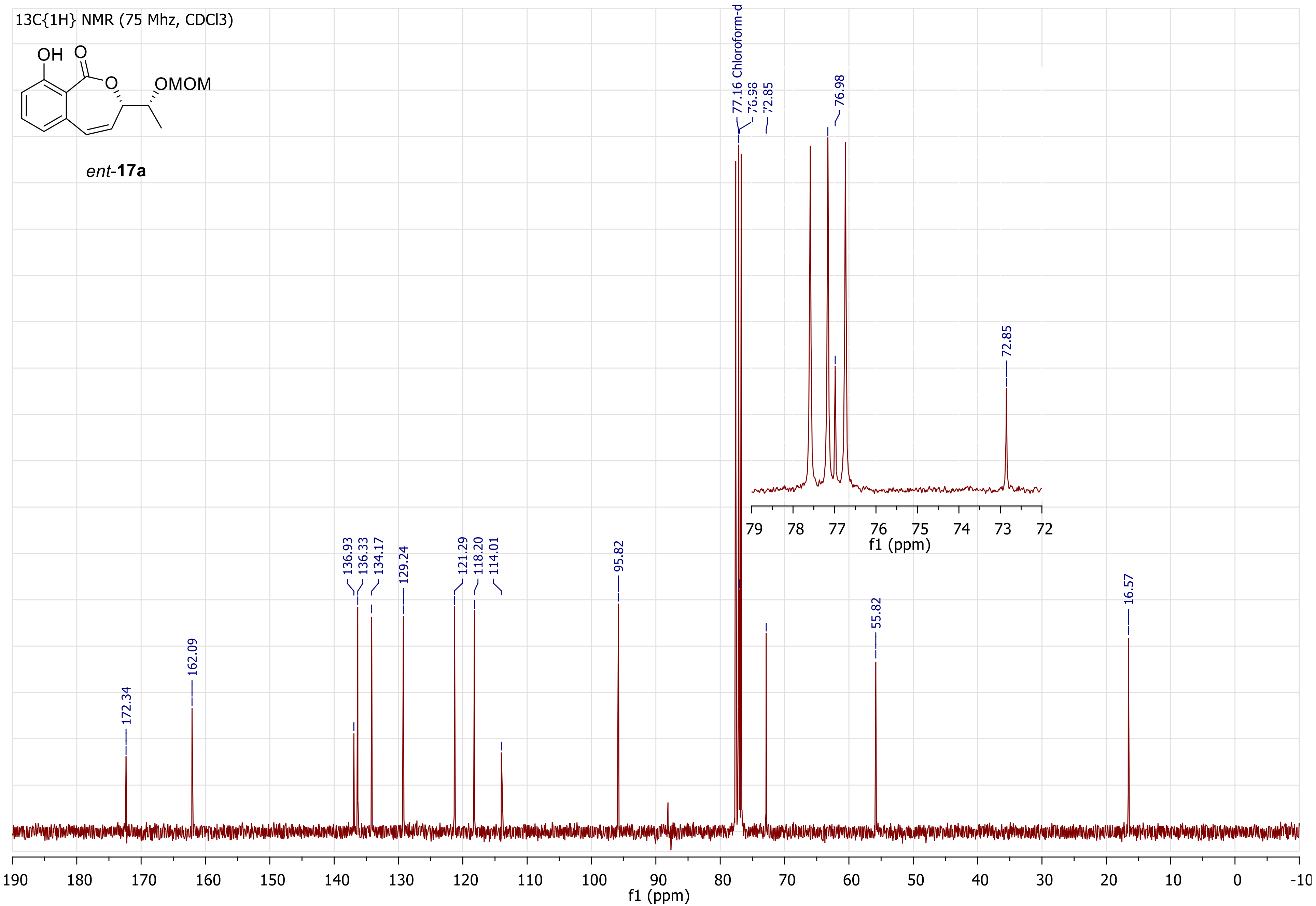

Figure S45. ^{13}C NMR spectrum of *ent*-**17a**.

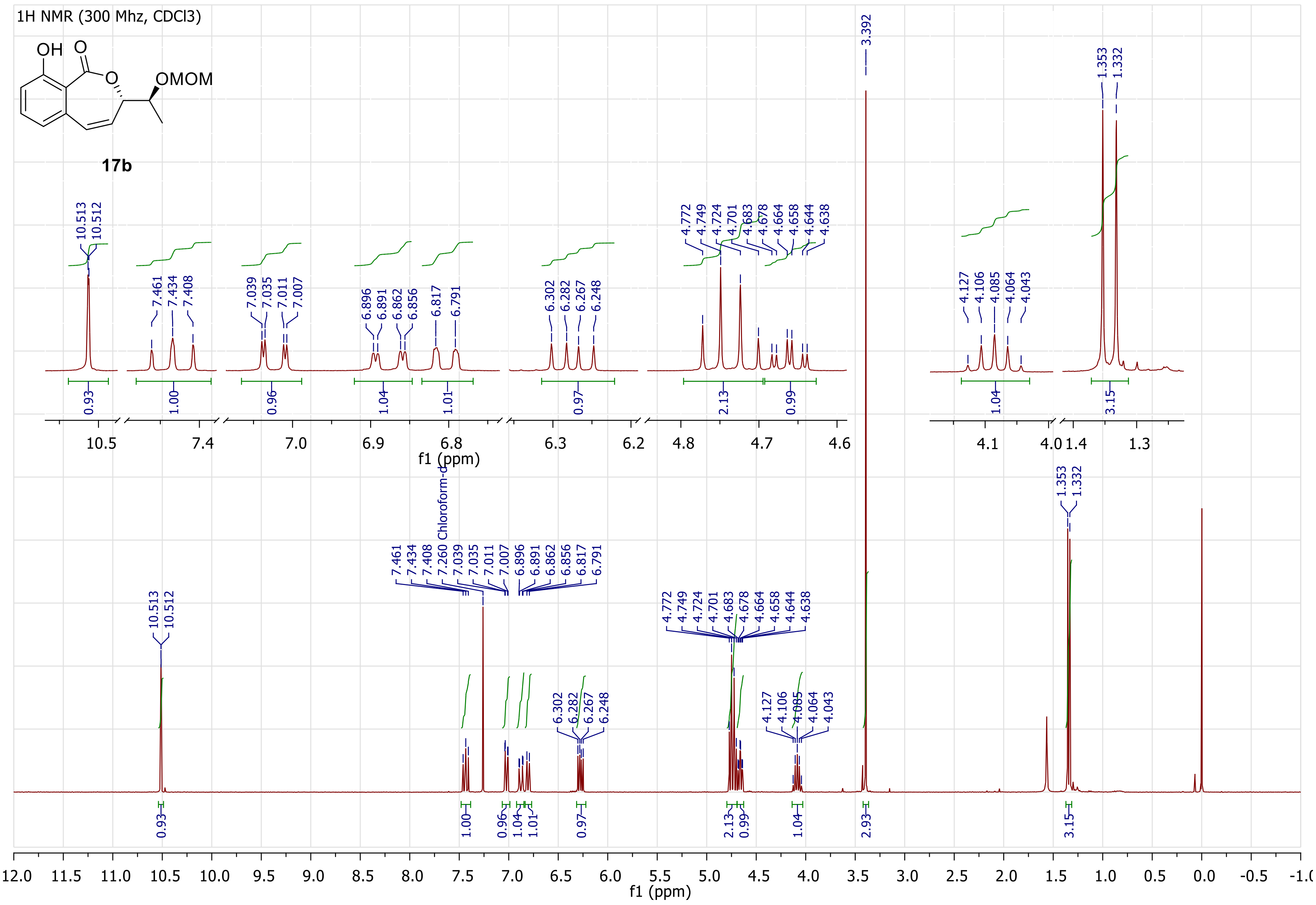

Figure S46. ^{1}H NMR spectrum of **17b**.

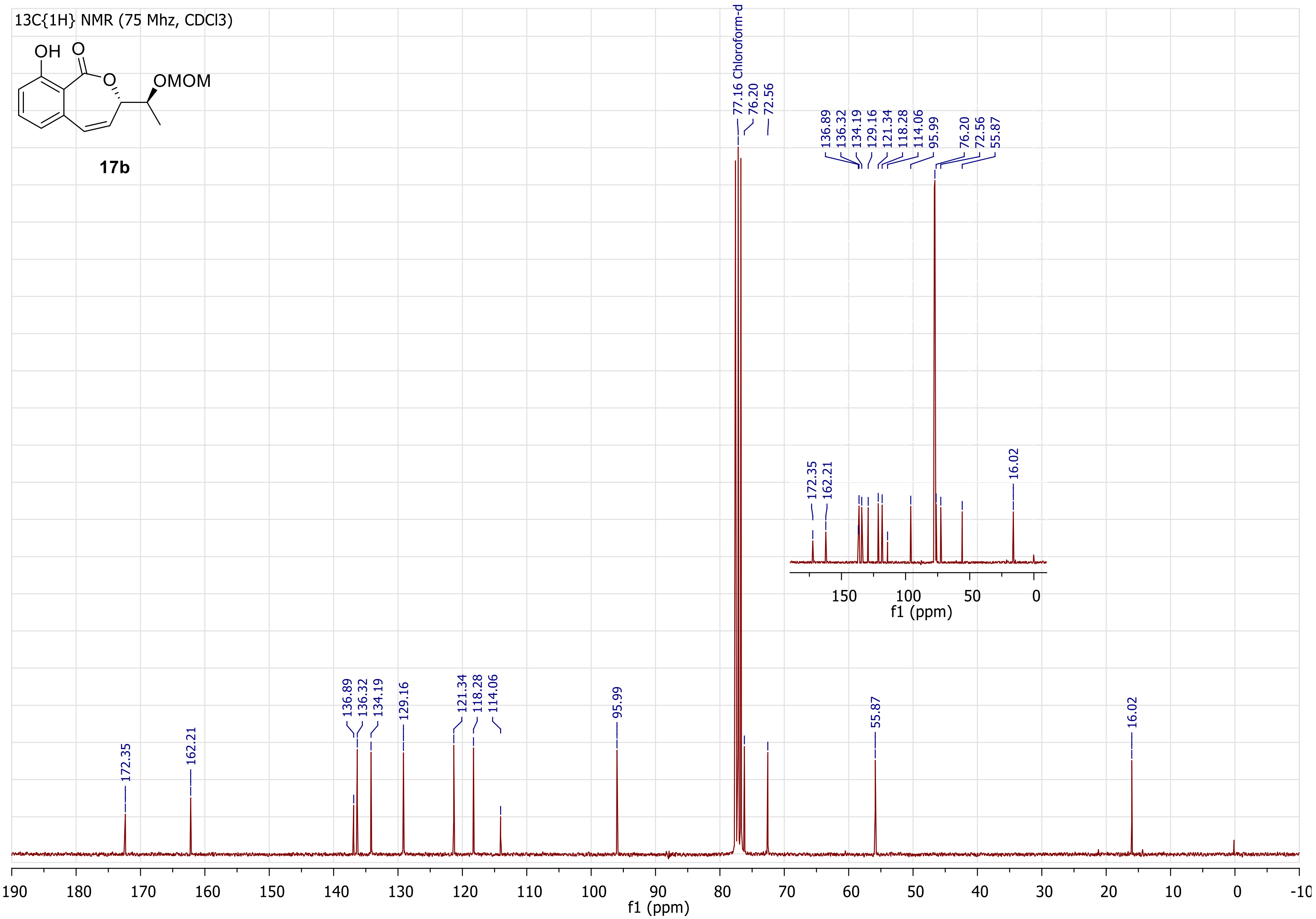

Figure S47. ^{13}C NMR spectrum of **17b**.

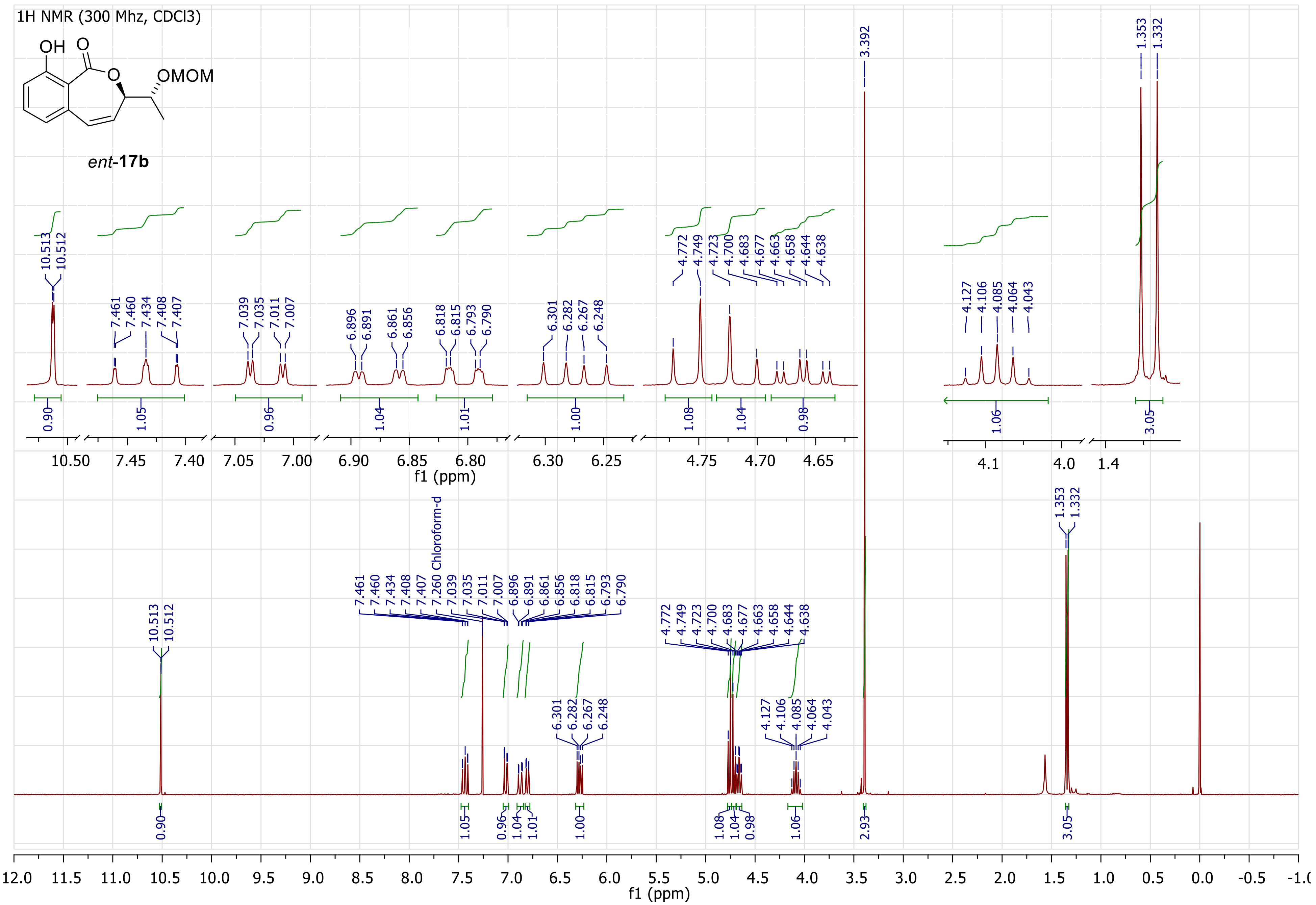

Figure S48. ^{1}H NMR spectrum of *ent*-**17b**.

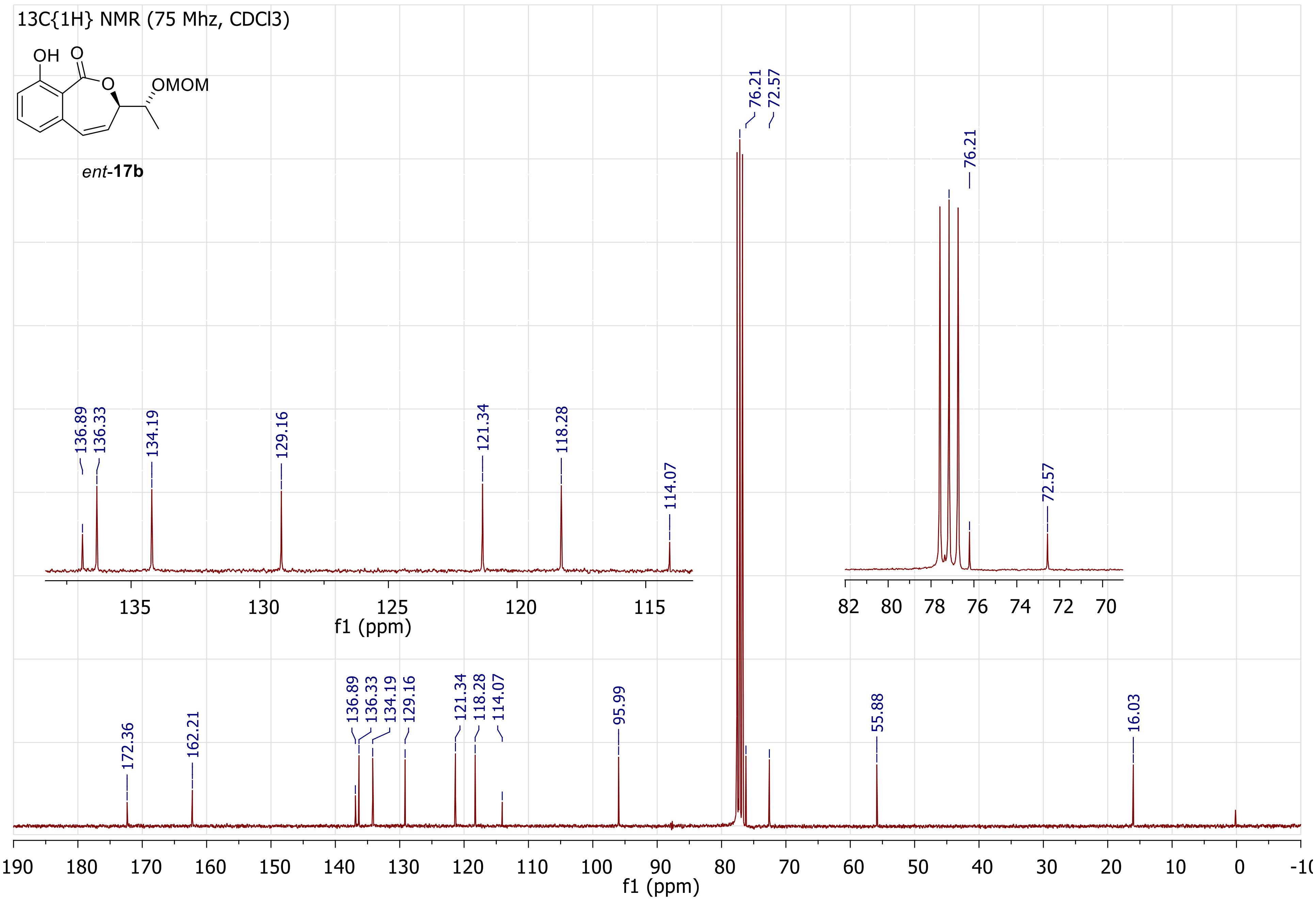

Figure S49. ^{13}C NMR spectrum of *ent*-**17b**.

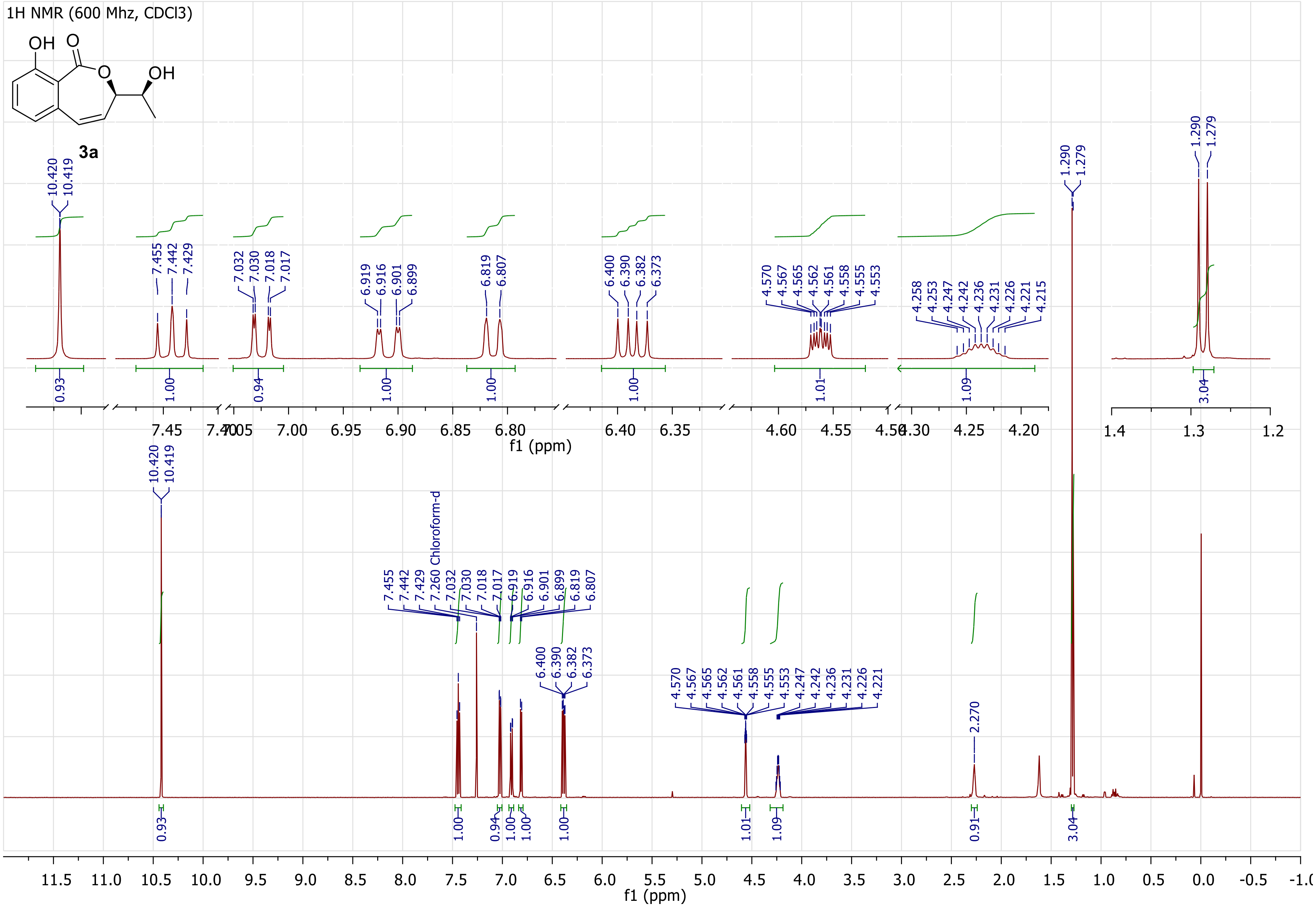

Figure S50. ^{1}H NMR spectrum of **3a** in $CDCl_3$.

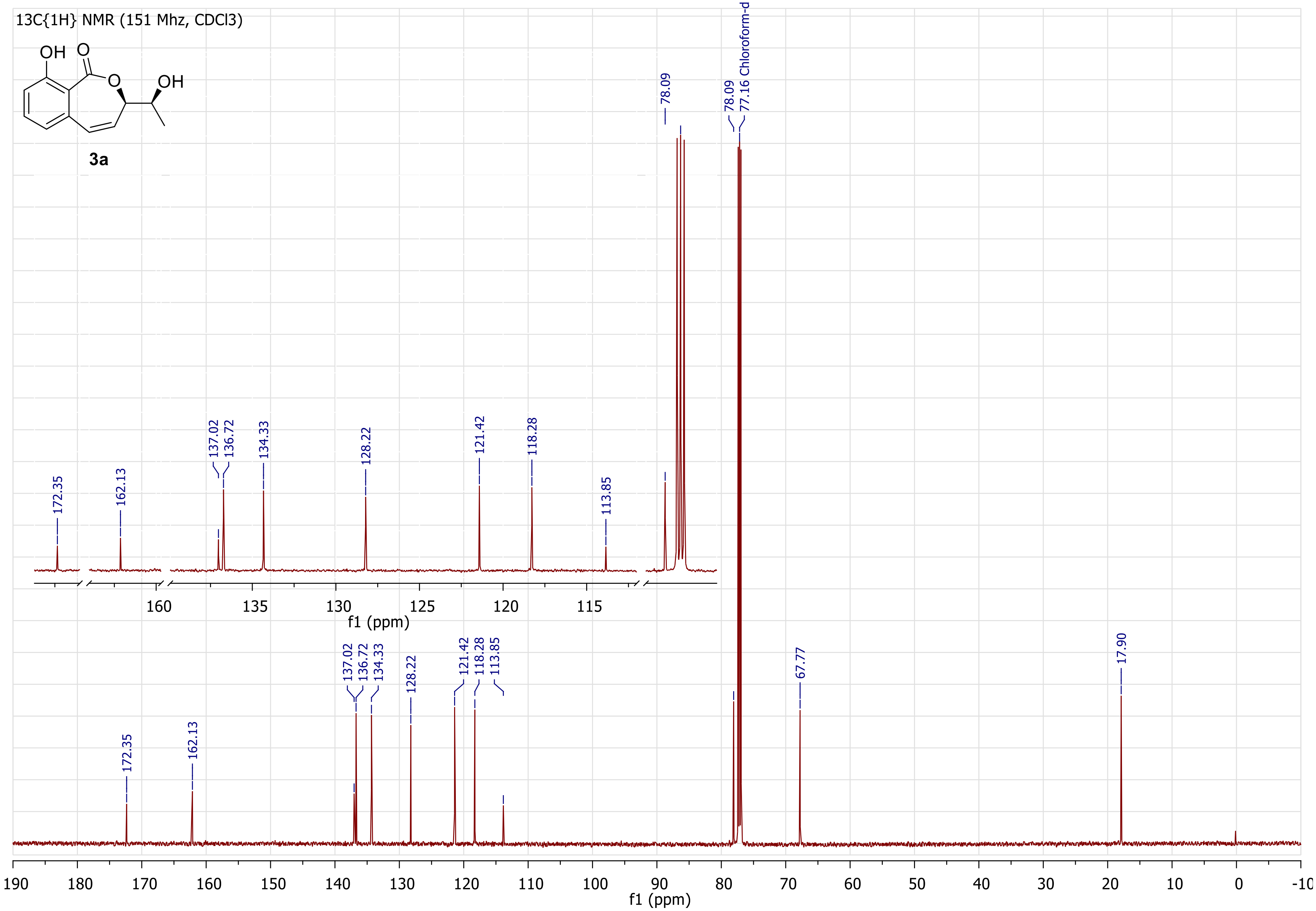

Figure S51. ^{13}C NMR spectrum of **3a** in $CDCl_3$.

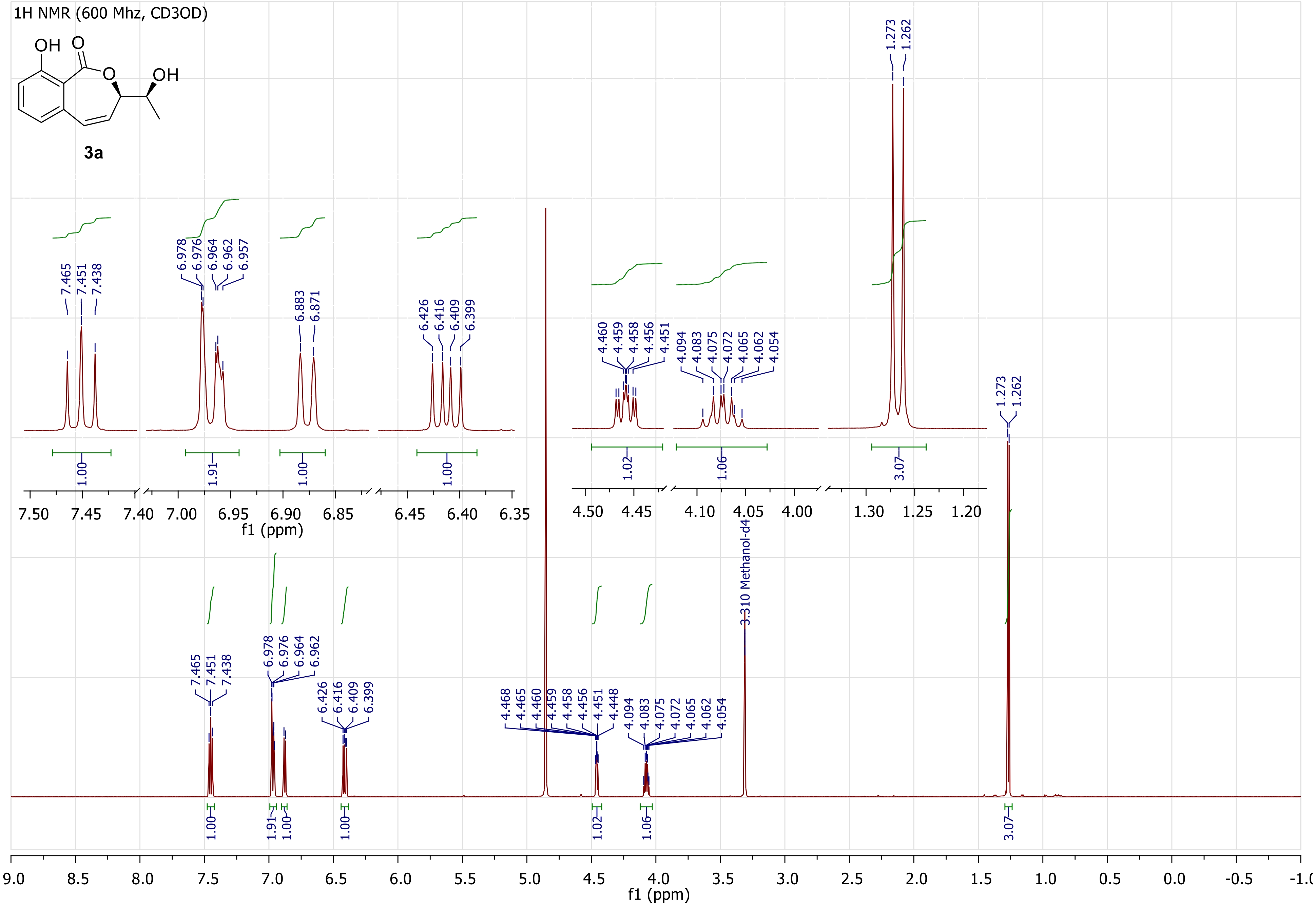

Figure S52. ¹H NMR spectrum of **3a** in CD_3OD.

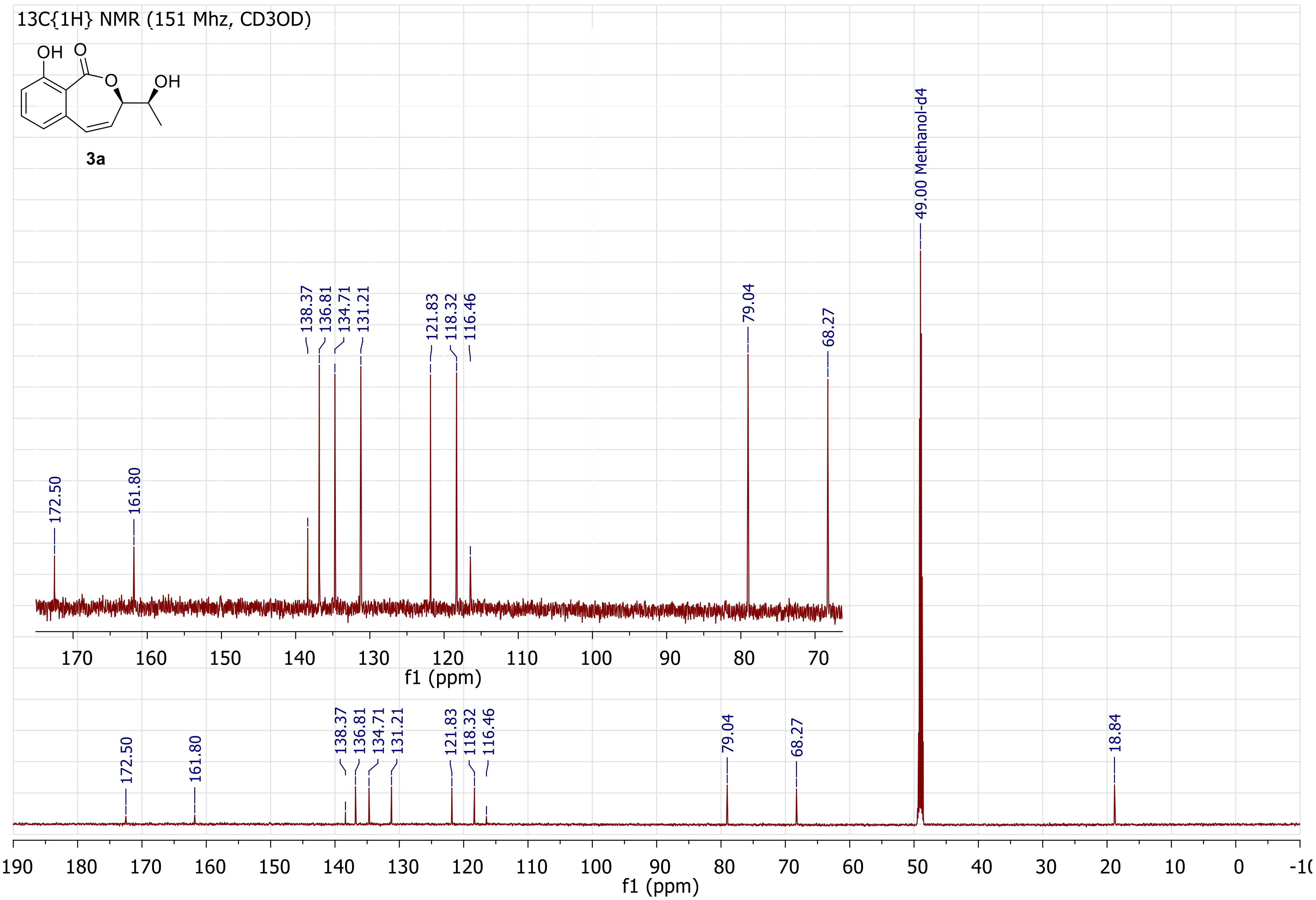

Figure S53. ^{13}C NMR spectrum of **3a** in CD_3OD.

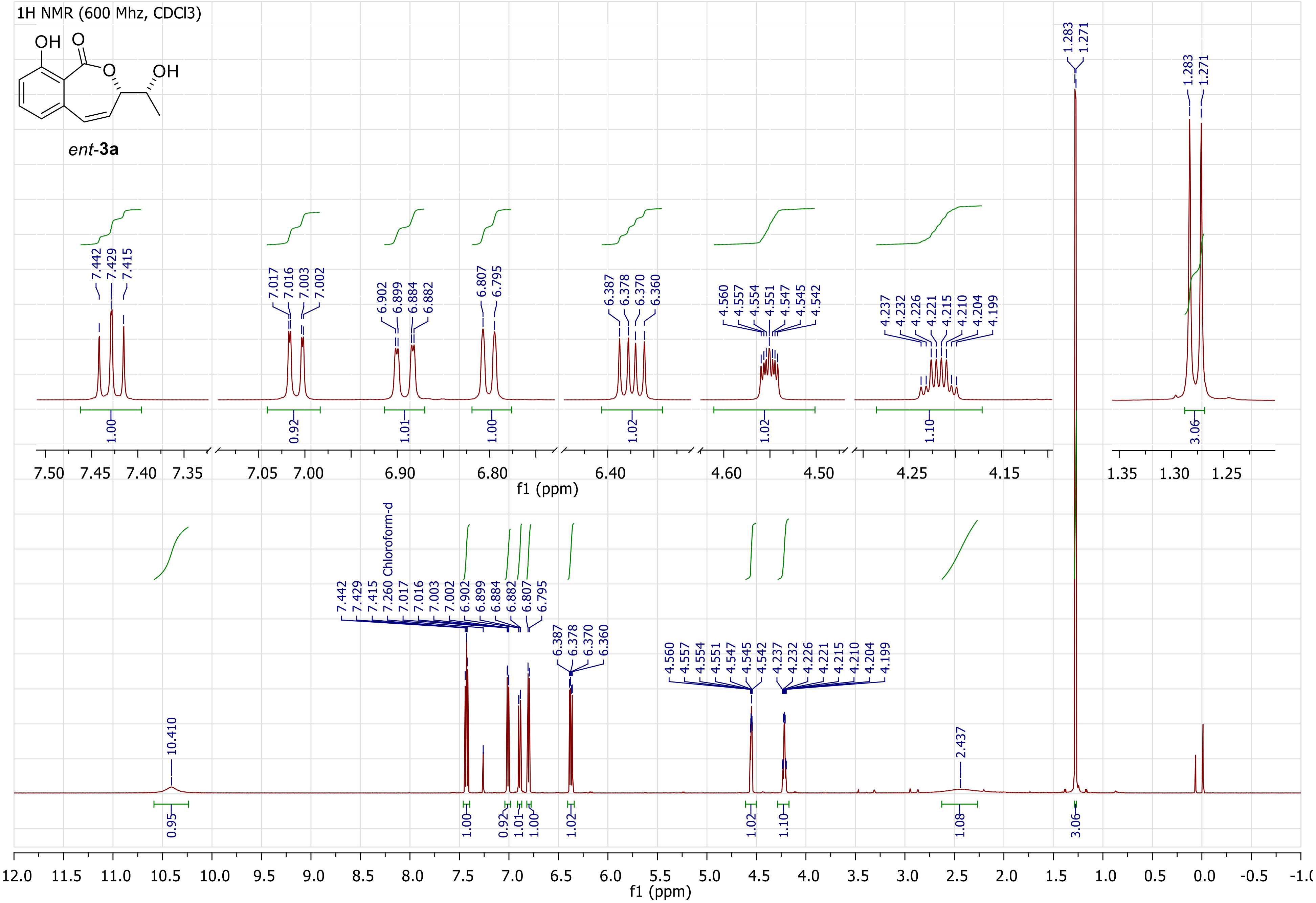

Figure S54. ^{1}H NMR spectrum of *ent*-**3a** in $CDCl_3$.

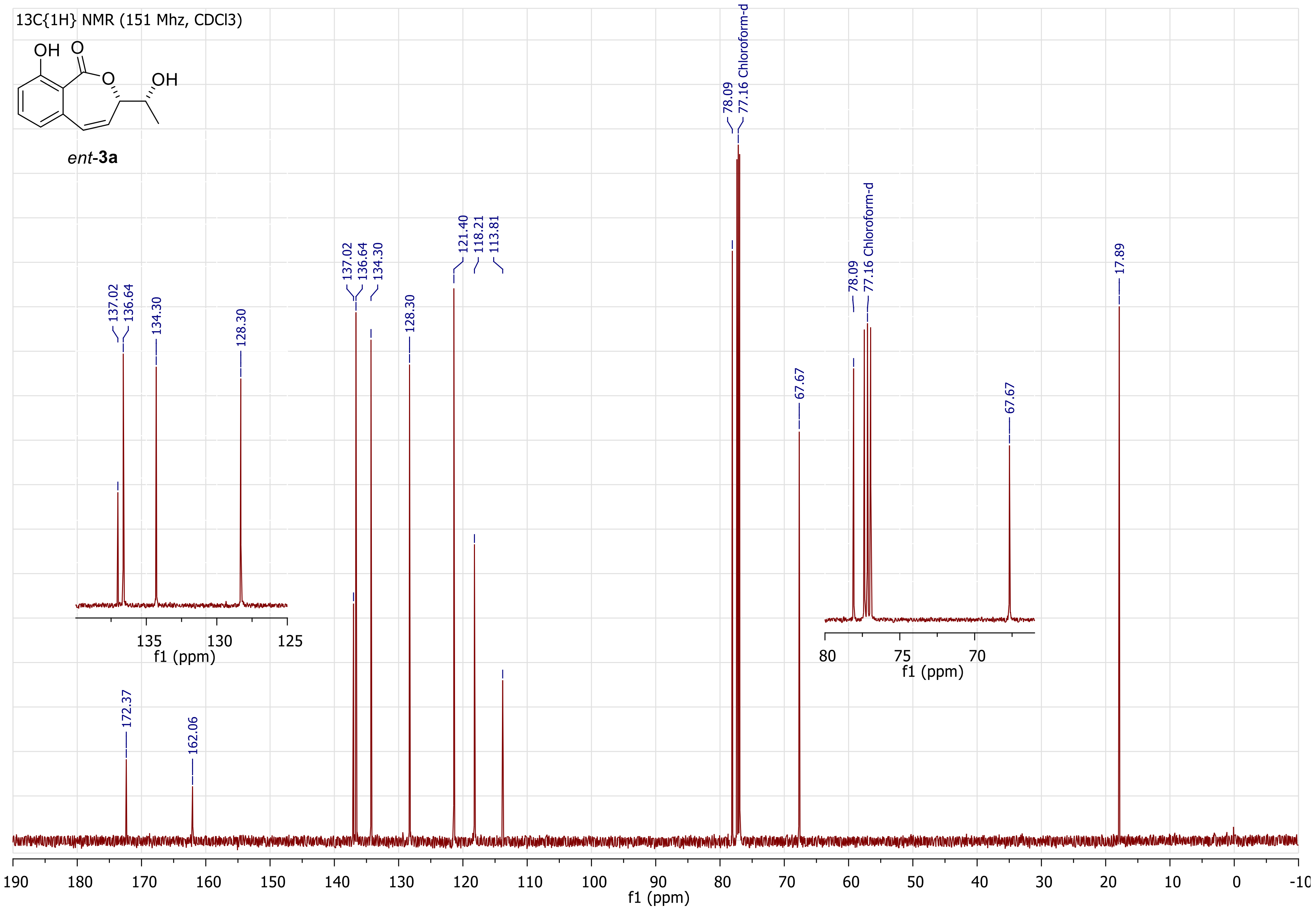

Figure S55. ^{13}C NMR spectrum of *ent*-**3a** in $CDCl_3$.

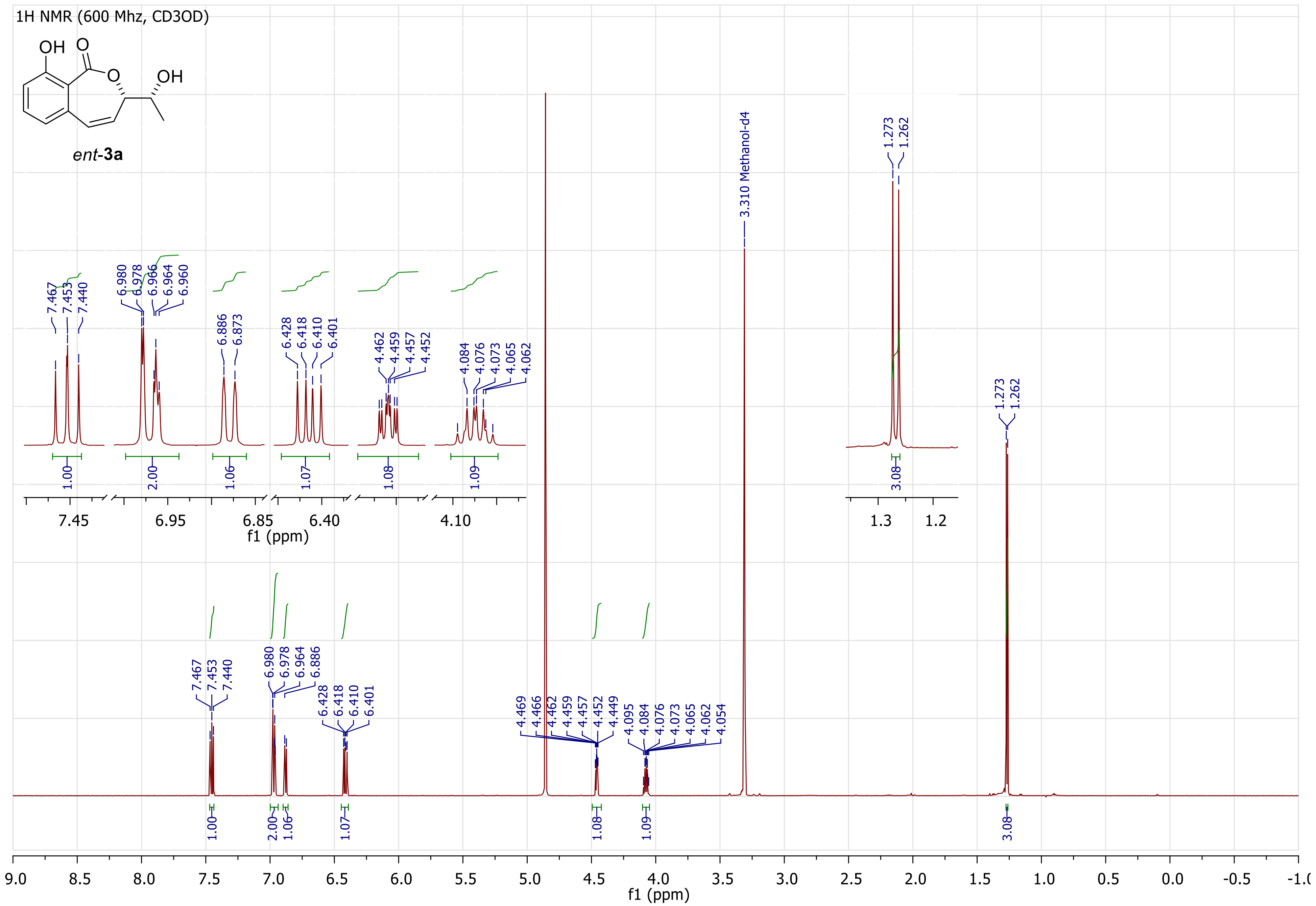

Figure S56. ^{1}H NMR spectrum of *ent*-**3a** in CD_3OD.

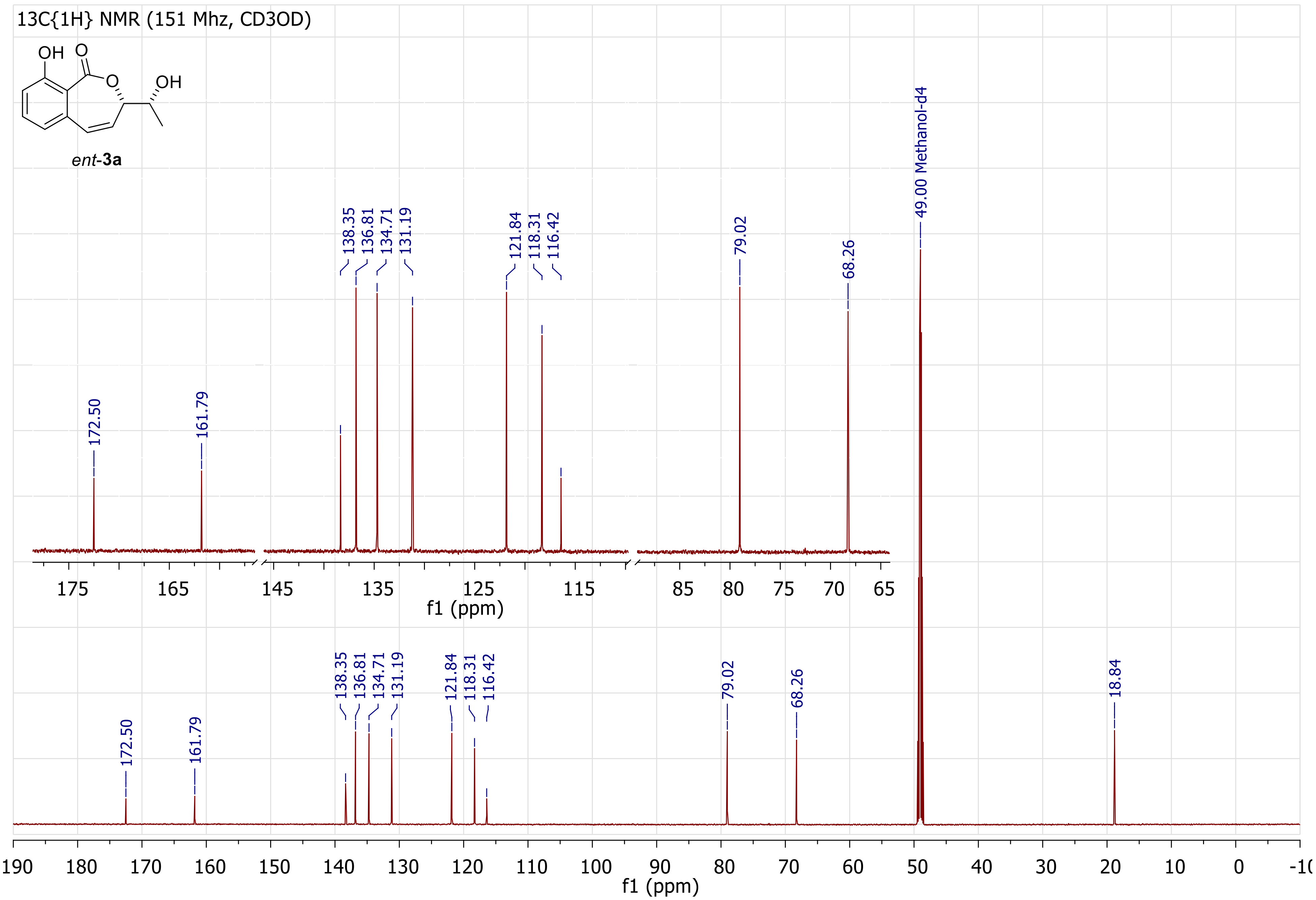

Figure S57. ^{13}C NMR spectrum of *ent*-**3a** in CD_3OD.

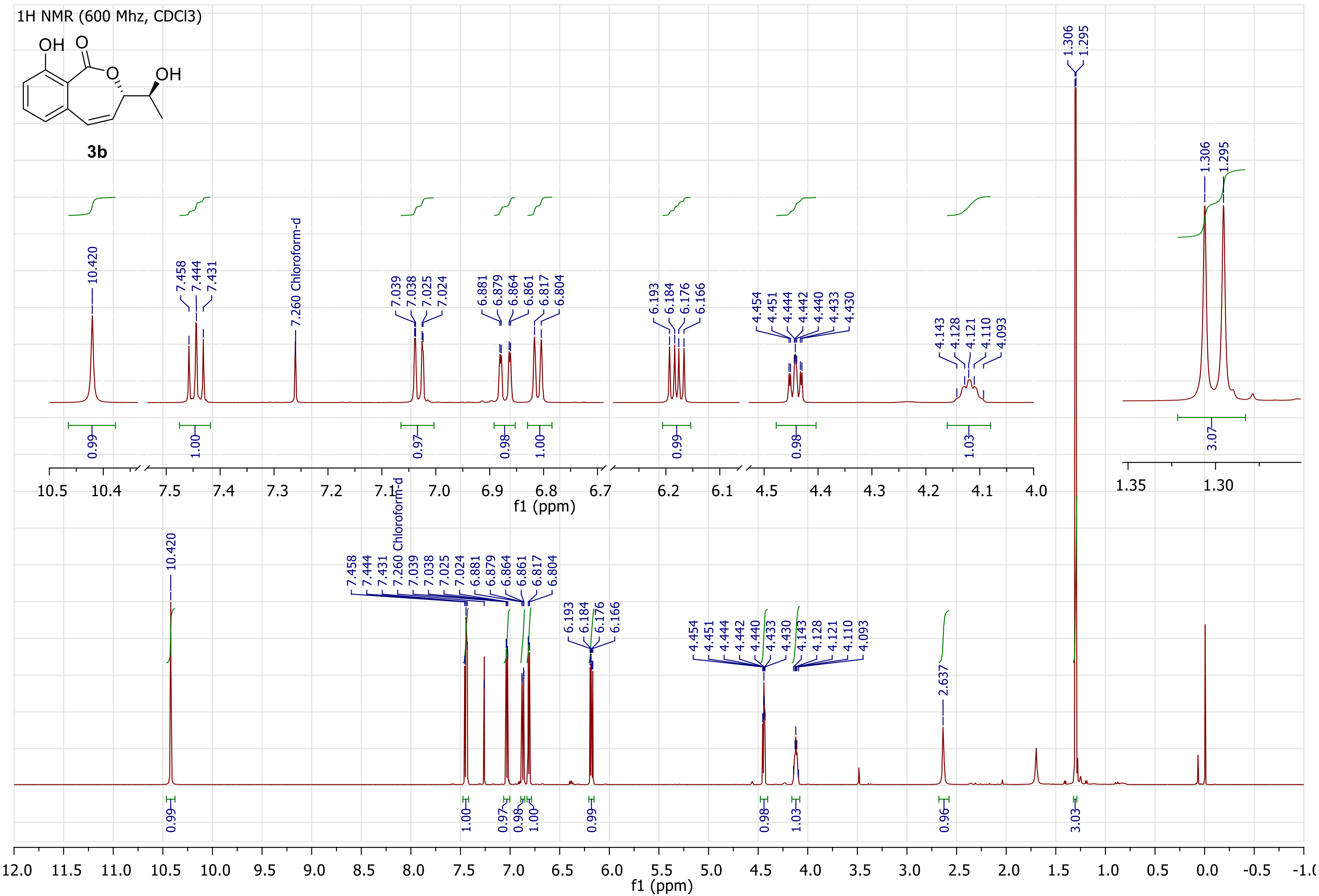

Figure S58. ^{1}H NMR spectrum of **3b** in $CDCl_3$.

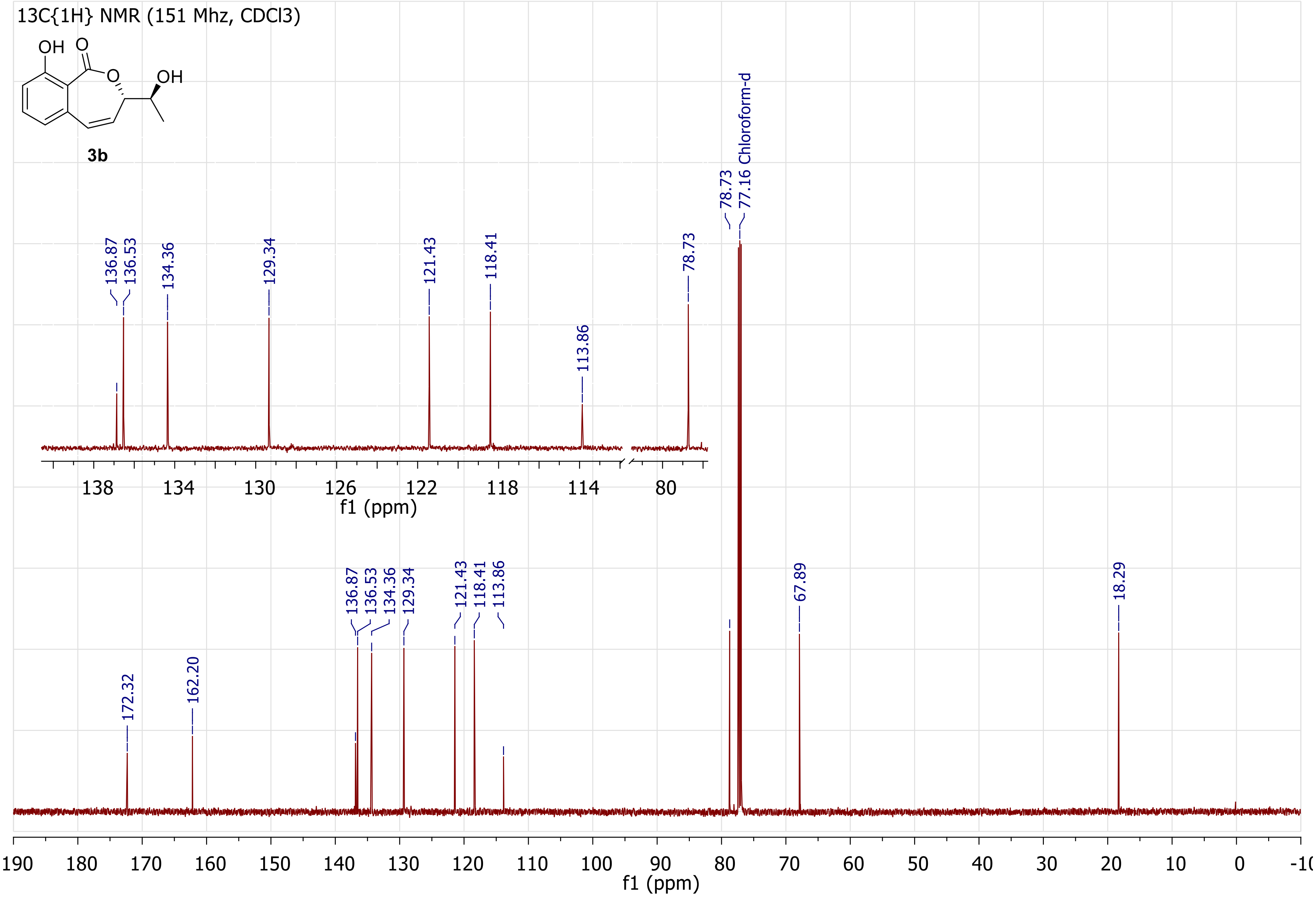

Figure S59. ^{13}C NMR spectrum of **3b** in $CDCl_3$.

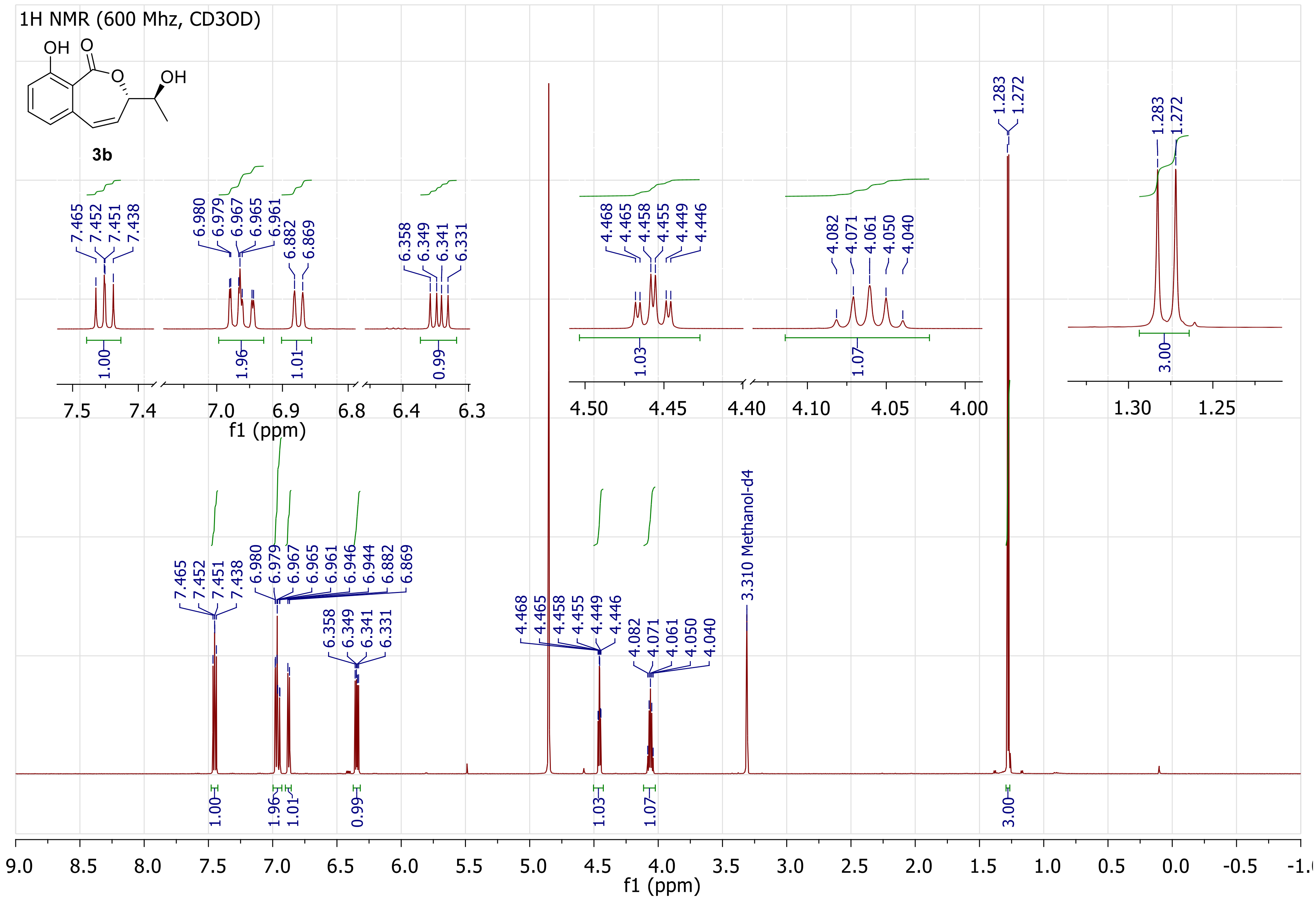

Figure S60. ^{1}H NMR spectrum of **3b** in CD_3OD.

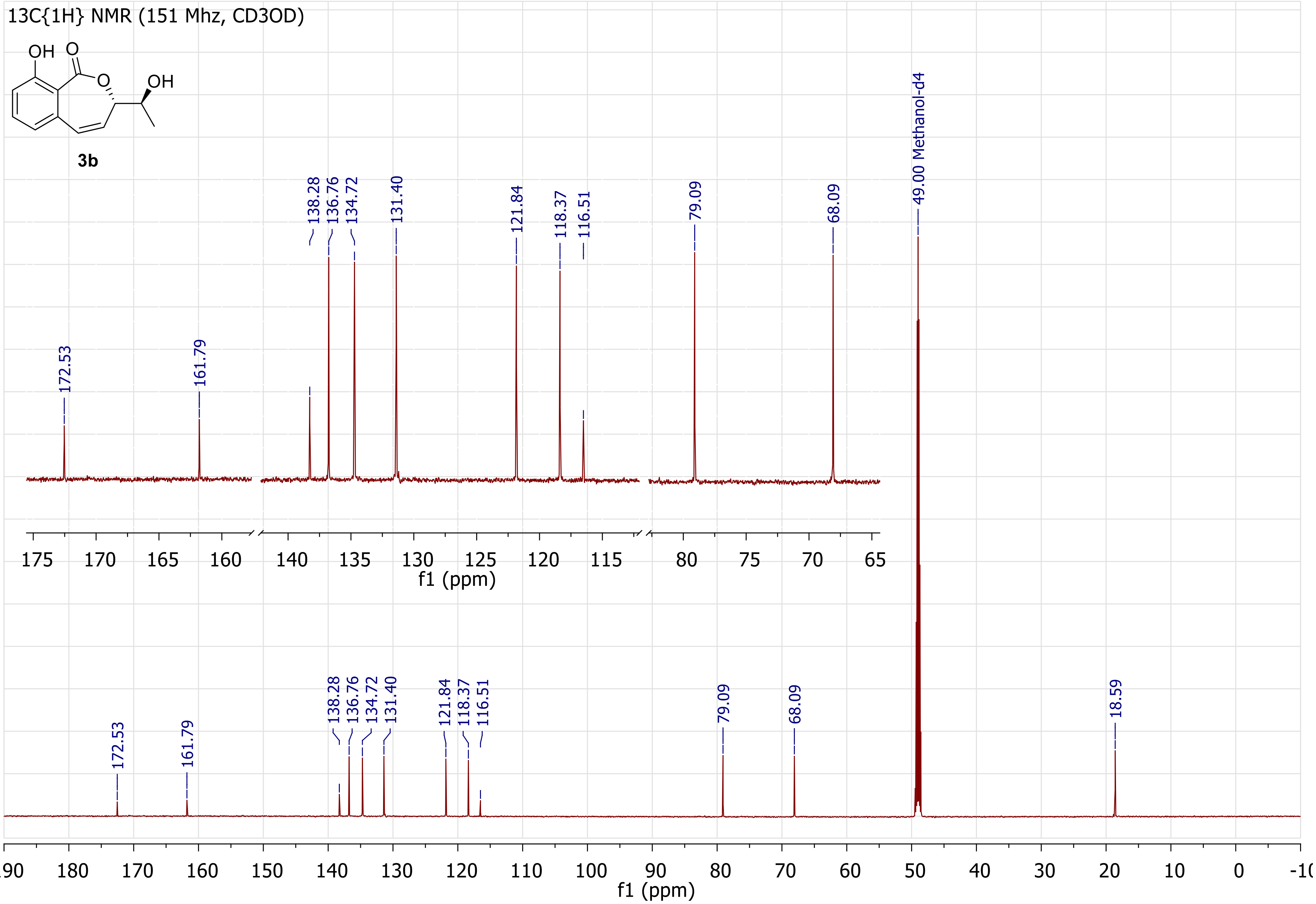

Figure S61. ^{13}C NMR spectrum of **3b** in CD_3OD.

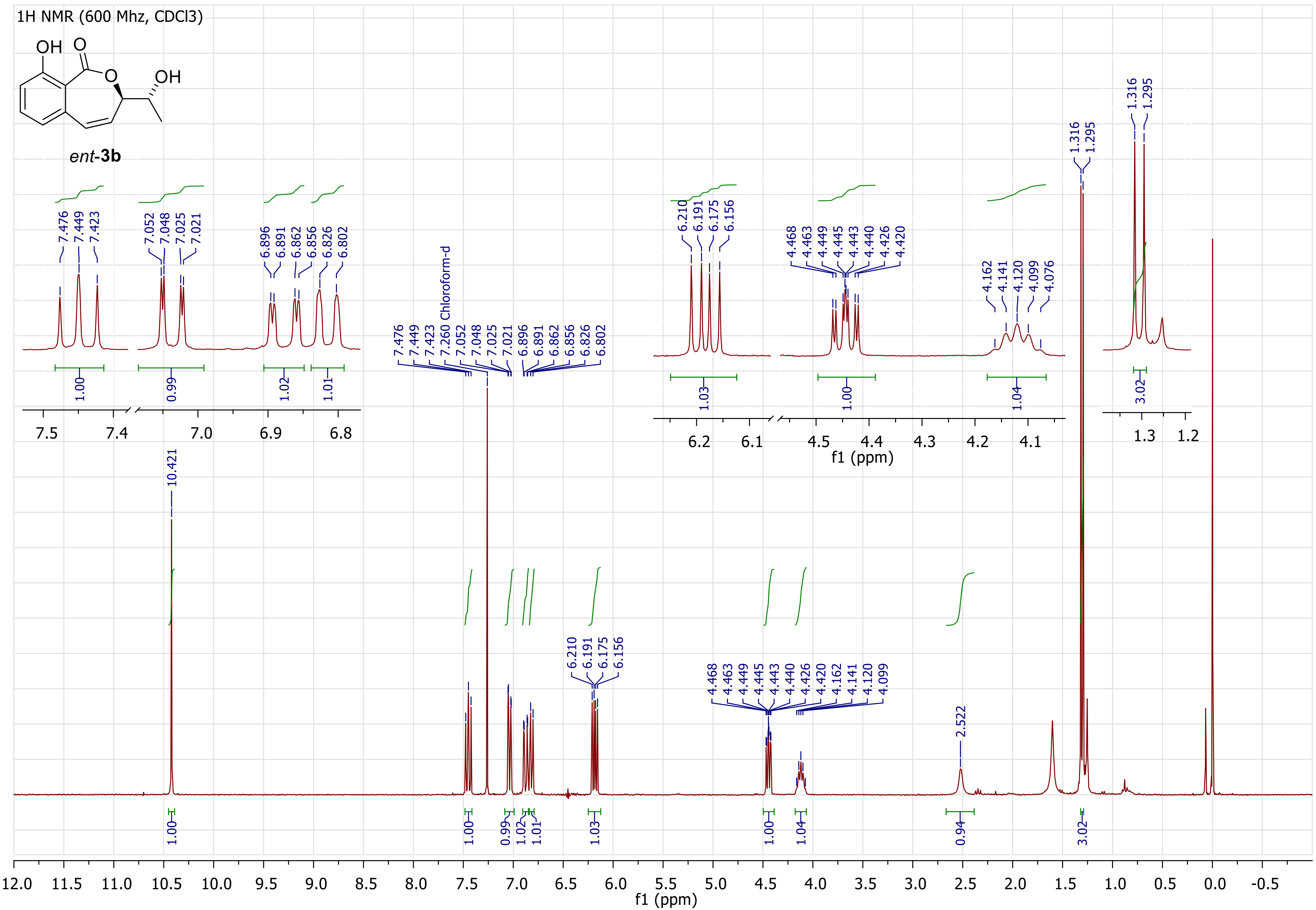

Figure S62. ^{1}H NMR spectrum of *ent*-**3b** in $CDCl_3$.

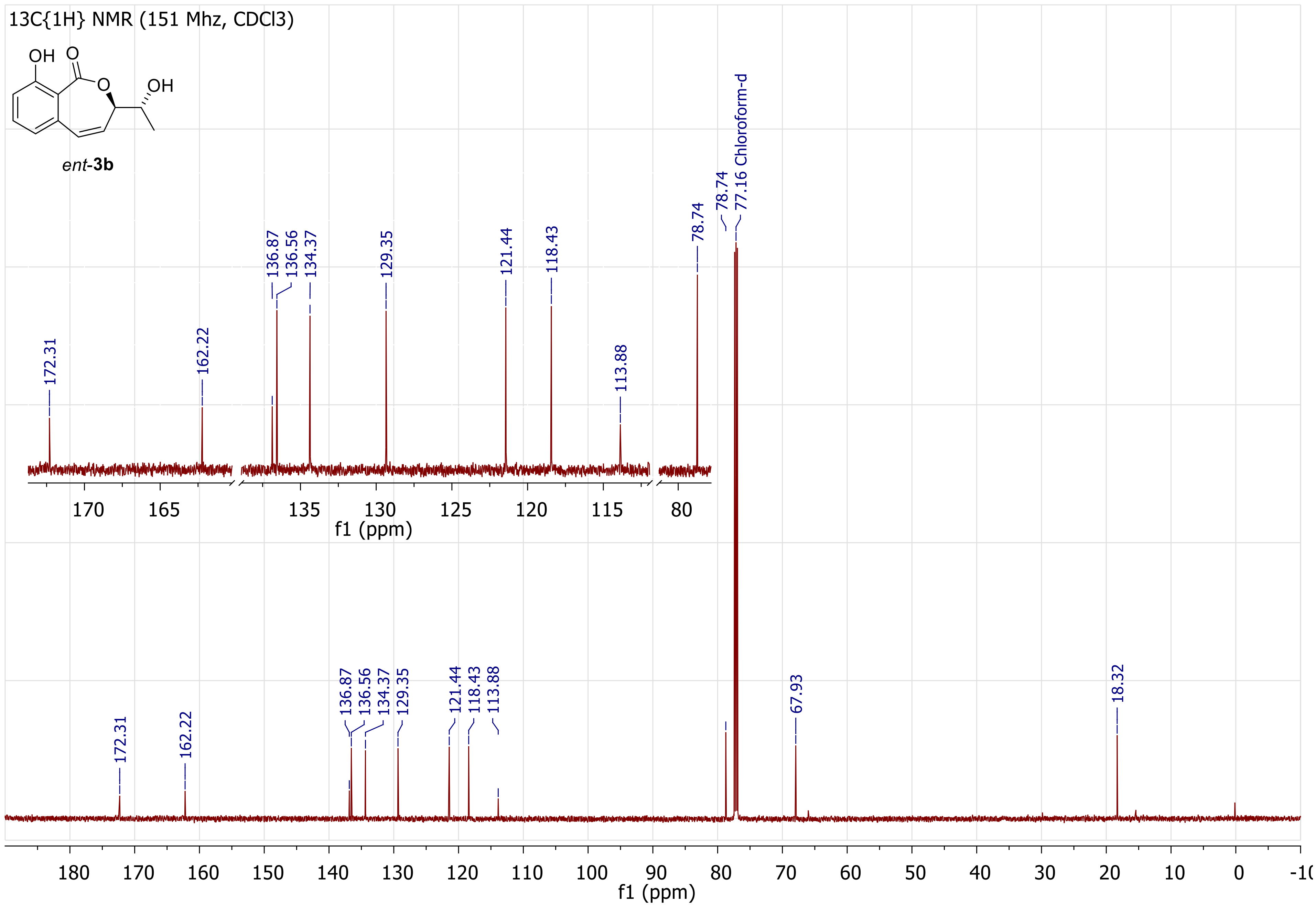

Figure S63. ^{13}C NMR spectrum of *ent*-**3b** in $CDCl_3$.

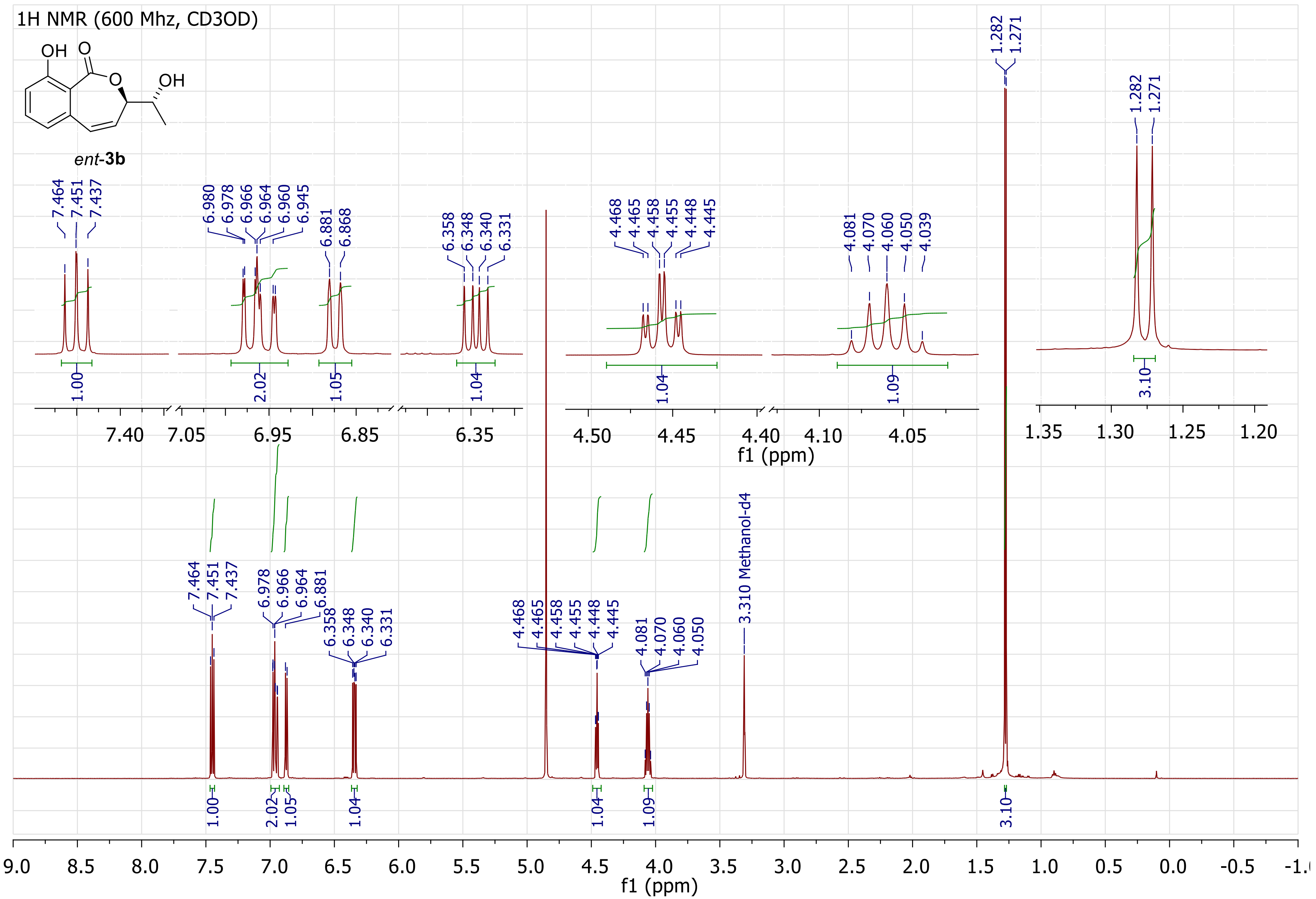

Figure S64. ^{1}H NMR spectrum of *ent*-**3b** in CD_3OD.

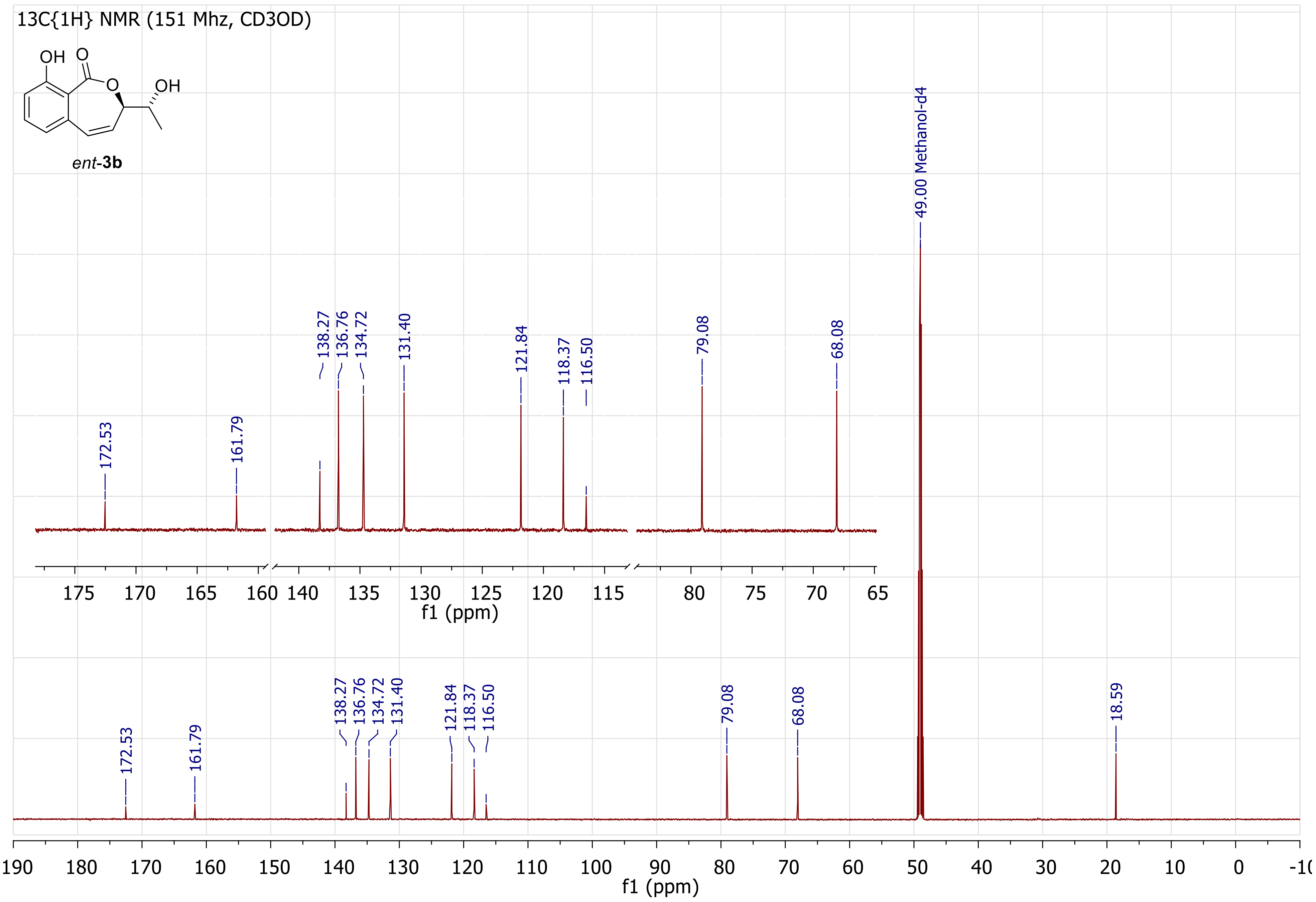

Figure S65. ^{13}C NMR spectrum of *ent*-**3b** in CD_3OD.

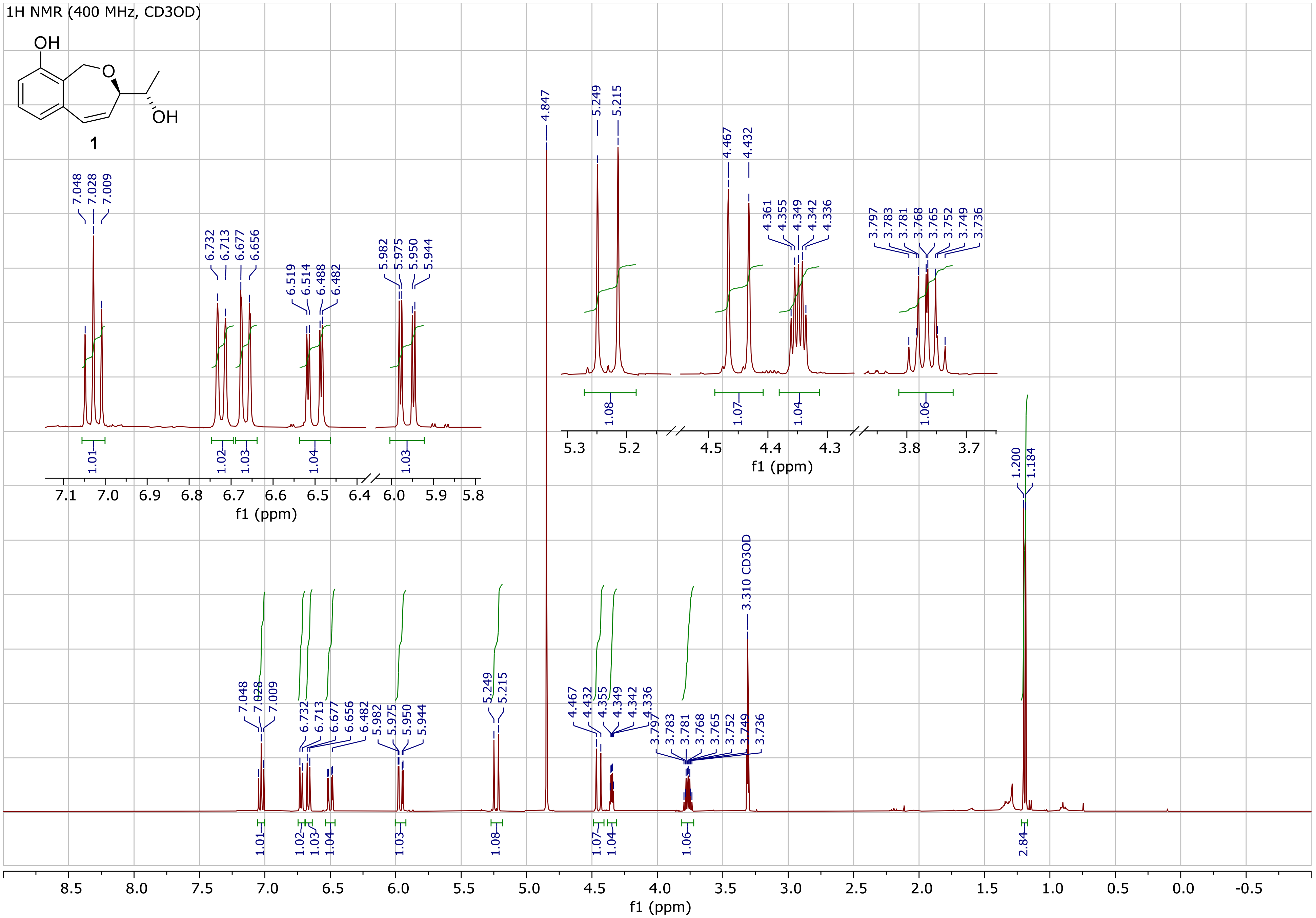

Figure S66. ^{1}H NMR spectrum of **1**.

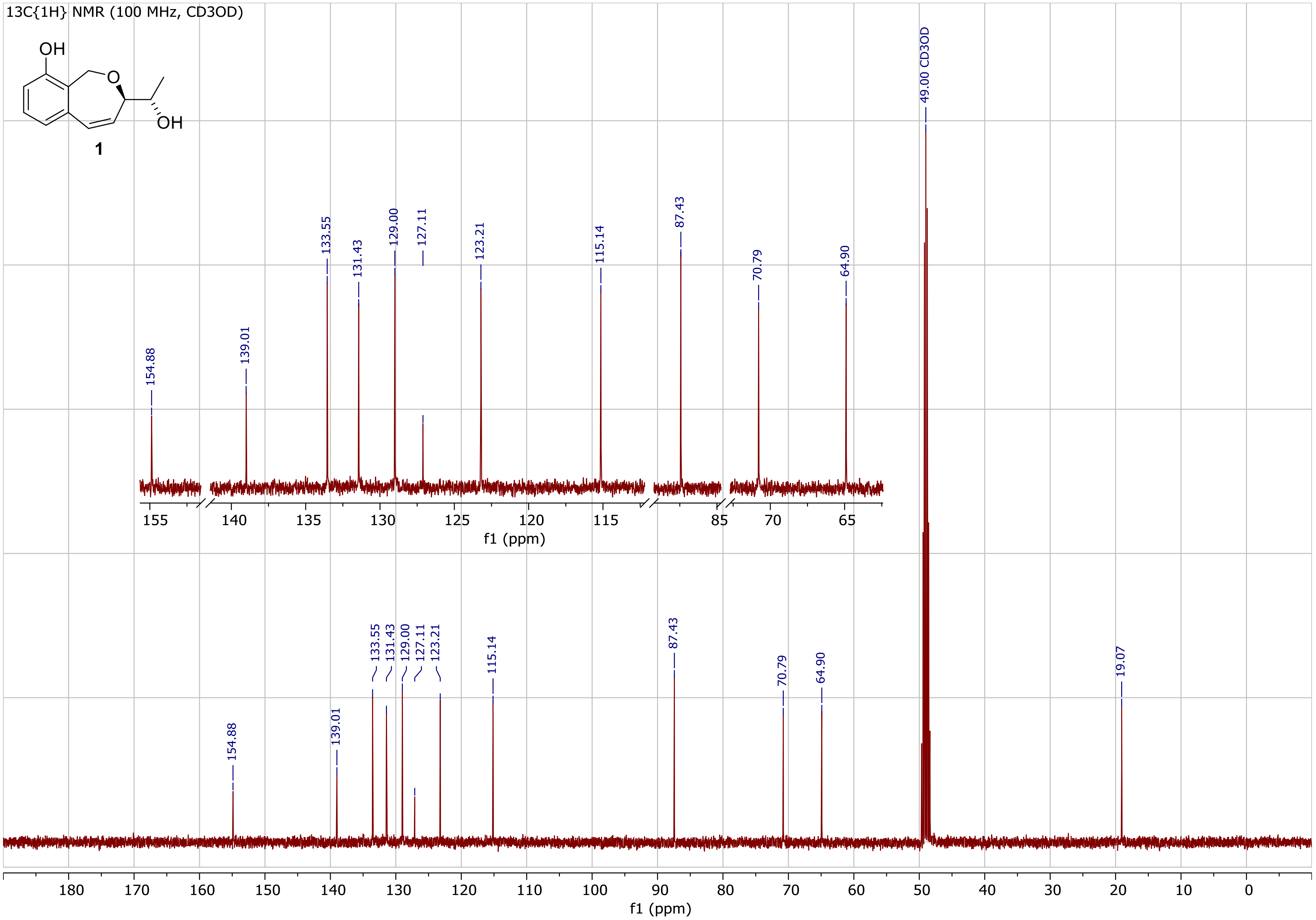

Figure S67. ^{13}C NMR spectrum of **1**.

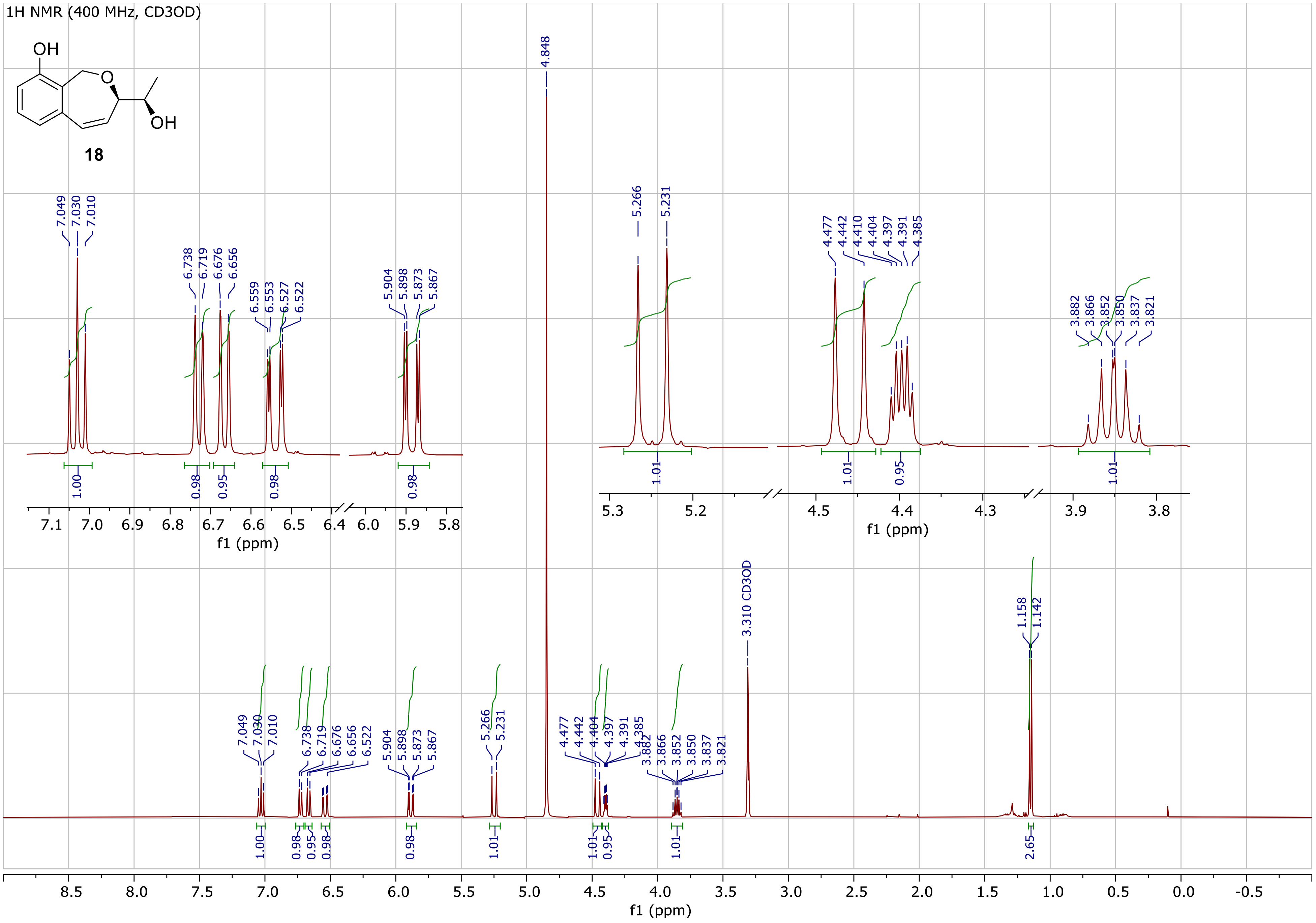

Figure S68. ^{1}H, NMR spectrum of **18**.

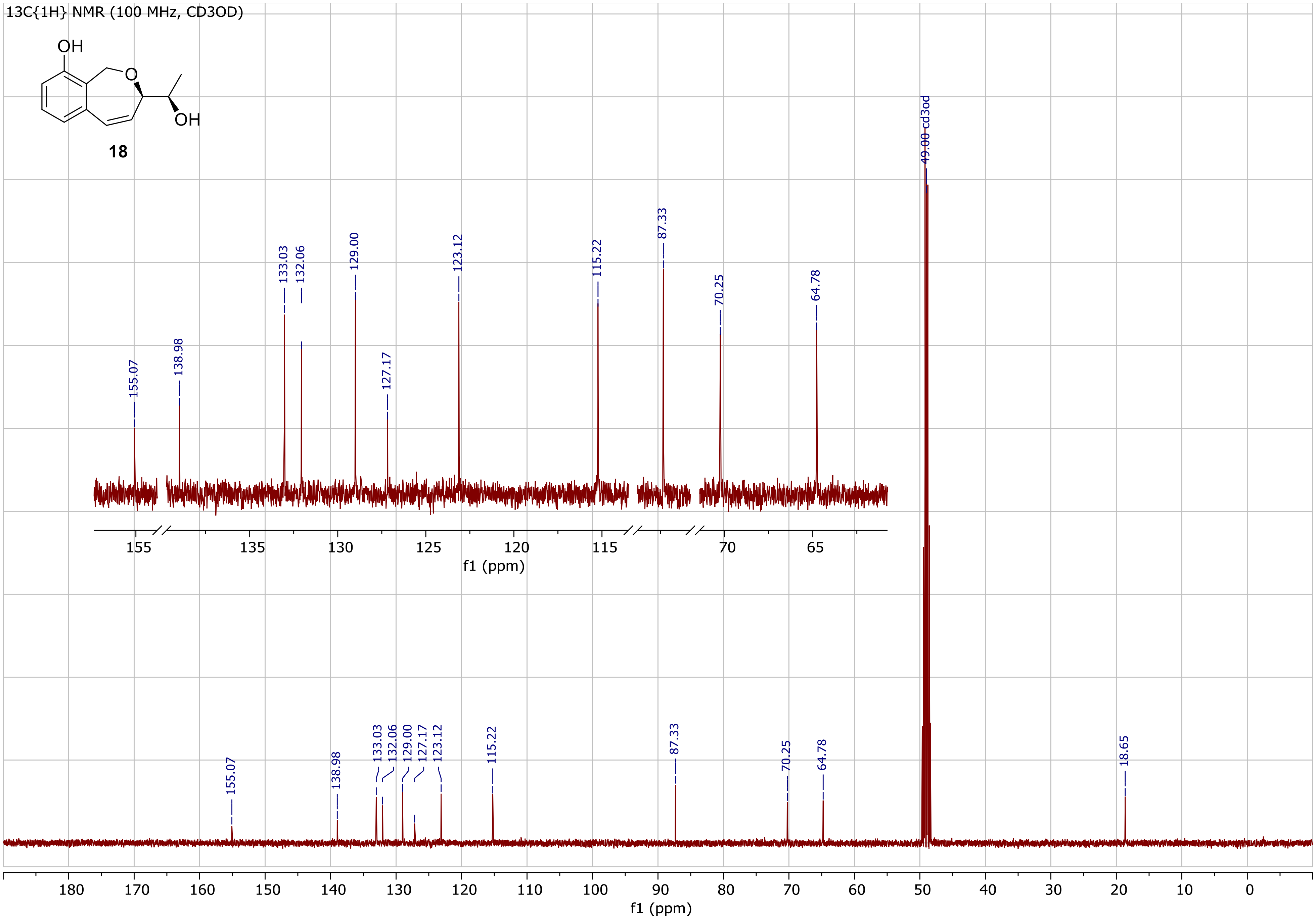

Figure S69. ^{13}C NMR spectrum of **18**.

HPLC Analytical method

Enantiomeric excesses were determined by HPLC (Agilent Technologies) using Chiralpak IC column (Daicel Chiral Technologies, dimensions 4.6 Ø x 250 mm, particle size 5 µm) using *n*-hexane/propan-2-ol (isocratic 90/10) as a mobile phase and detected by UV at 254 nm.

UV detector: 254 nm
Sample injection: 12 µL (C = 1mg/mL)
Isocratic 90/10 n-hexane/iPrOH
Flow rate: 0.6 mL/min
CHIRALPAK® IC

Retention times: **3a** 45.8 min, *ent*-**3a** 49.2 min,
3b 42.5 min, *ent*-**3b** 35.8 min.

Data File C:\CHEM32\1\DATA\MICHAL\TCA-7-ANTI.D
Sample Name: IC, 90/10, 0.6ml, 12micoL, 254nm

===

Acq. Operator : tomas
Acq. Instrument : Instrument 1 Location : -
Injection Date : 1/18/2023 2:36:19 AM
Acq. Method : C:\CHEM32\1\METHODS\ROBO.M
Last changed : 1/18/2023 2:23:18 AM by tomas
(modified after loading)
Analysis Method : C:\CHEM32\1\METHODS\ROBO.M
Last changed : 1/19/2023 2:16:41 AM by tomas
(modified after loading)
Additional Info : Peak(s) manually integrated

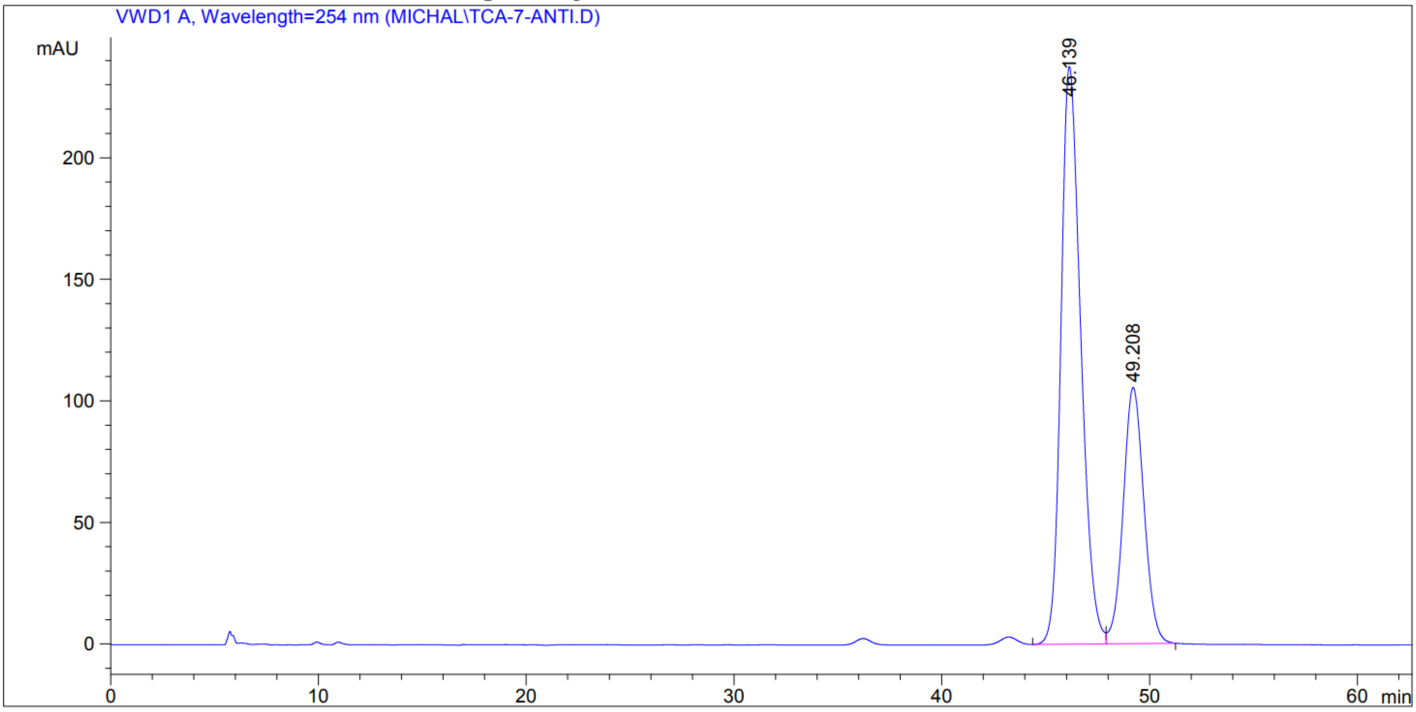

===

Area Percent Report

===

Sorted By : Signal
Multiplier: : 1.0000
Dilution: : 1.0000
Use Multiplier & Dilution Factor with ISTDs

Signal 1: VWD1 A, Wavelength=254 nm

Peak #	RetTime [min]	Type	Width [min]	Area mAU *s	Height [mAU]	Area %
1	46.139	VV	1.0138	1.57859e4	237.63568	68.2822
2	49.208	VB	1.0617	7332.72803	105.44589	31.7178

Totals : 2.31187e4 343.08157

===

*** End of Report ***

Figure S70. *HPLC analysis of a mixture of 3a and ent-3a.*

```
Data File C:\CHEM32\1\DATA\MICHAL\TCA-8-TC-464.D
Sample Name: IC, 90/10, 0.6ml, 12micoL, 254nm

=====================================================================
Acq. Operator   : tomas
Acq. Instrument : Instrument 1                    Location :   -
Injection Date  : 1/18/2023 3:44:19 AM
Acq. Method     : C:\CHEM32\1\METHODS\ROBO.M
Last changed    : 1/18/2023 3:39:17 AM by tomas
                  (modified after loading)
Analysis Method : C:\CHEM32\1\METHODS\ROBO.M
Last changed    : 1/19/2023 2:16:41 AM by tomas
                  (modified after loading)
Additional Info : Peak(s) manually integrated
```

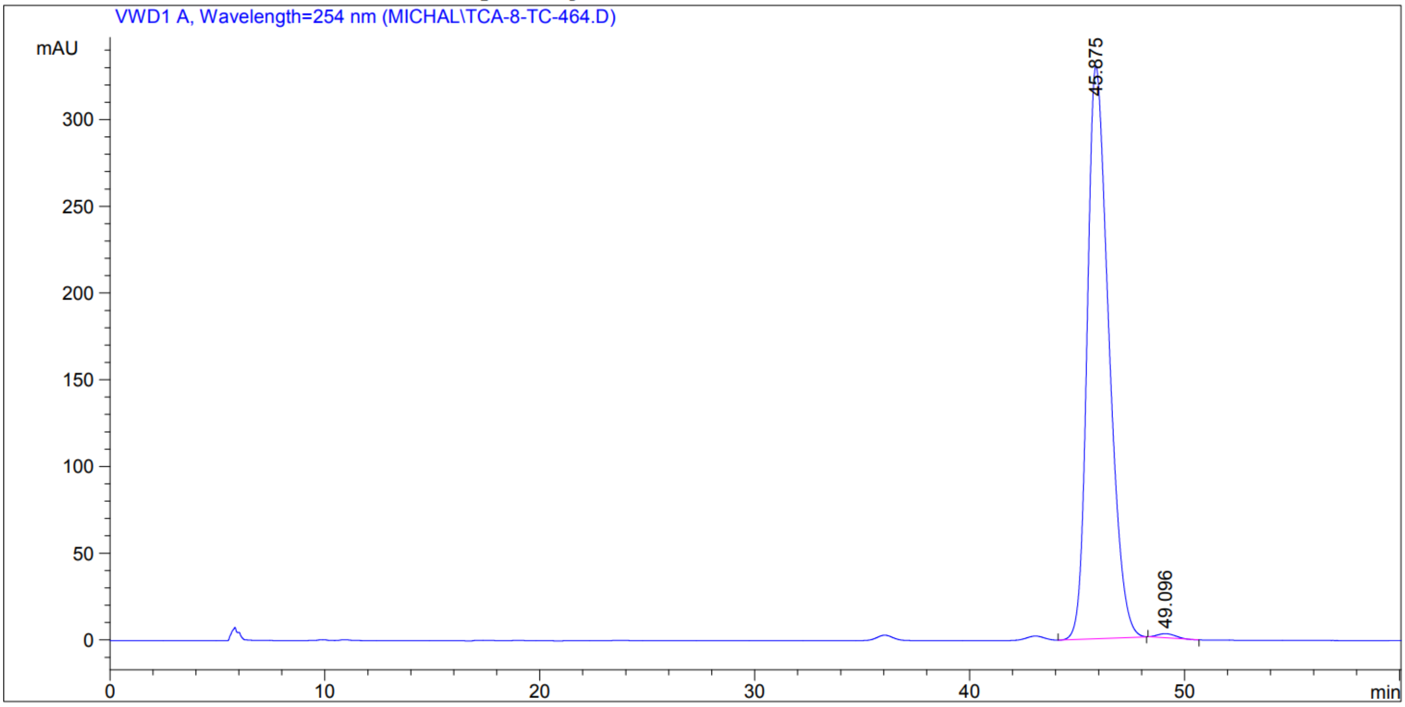


```
=====================================================================
                         Area Percent Report
=====================================================================

Sorted By             :      Signal
Multiplier:                   :      1.0000
Dilution:                     :      1.0000
Use Multiplier & Dilution Factor with ISTDs


Signal 1: VWD1 A, Wavelength=254 nm

Peak RetTime Type  Width     Area      Height     Area
  #   [min]        [min]  mAU    *s   [mAU    ]     %
----|-------|----|-------|----------|----------|--------|
   1  45.875 VB    1.0570 2.29933e4  330.19550  99.4045
   2  49.096 BB    0.6901  137.74297    2.38924   0.5955

Totals :                  2.31311e4  332.58474


=====================================================================
                          *** End of Report ***
```

Figure S71. *HPLC analysis of synthetic 3a.*

```
Data File C:\CHEM32\1\DATA\MICHAL\TCA-15-SYN.D
Sample Name: IC, 90/10, 0.6ml, 12micoL, 254nm

=====================================================================
Acq. Operator   : tomas
Acq. Instrument : Instrument 1                    Location :   -
Injection Date  : 1/19/2023 2:07:27 AM
Acq. Method     : C:\CHEM32\1\METHODS\ROBO.M
Last changed    : 1/19/2023 2:01:18 AM by tomas
                  (modified after loading)
Analysis Method : C:\CHEM32\1\METHODS\ROBO.M
Last changed    : 1/19/2023 2:16:41 AM by tomas
                  (modified after loading)
Additional Info : Peak(s) manually integrated
```

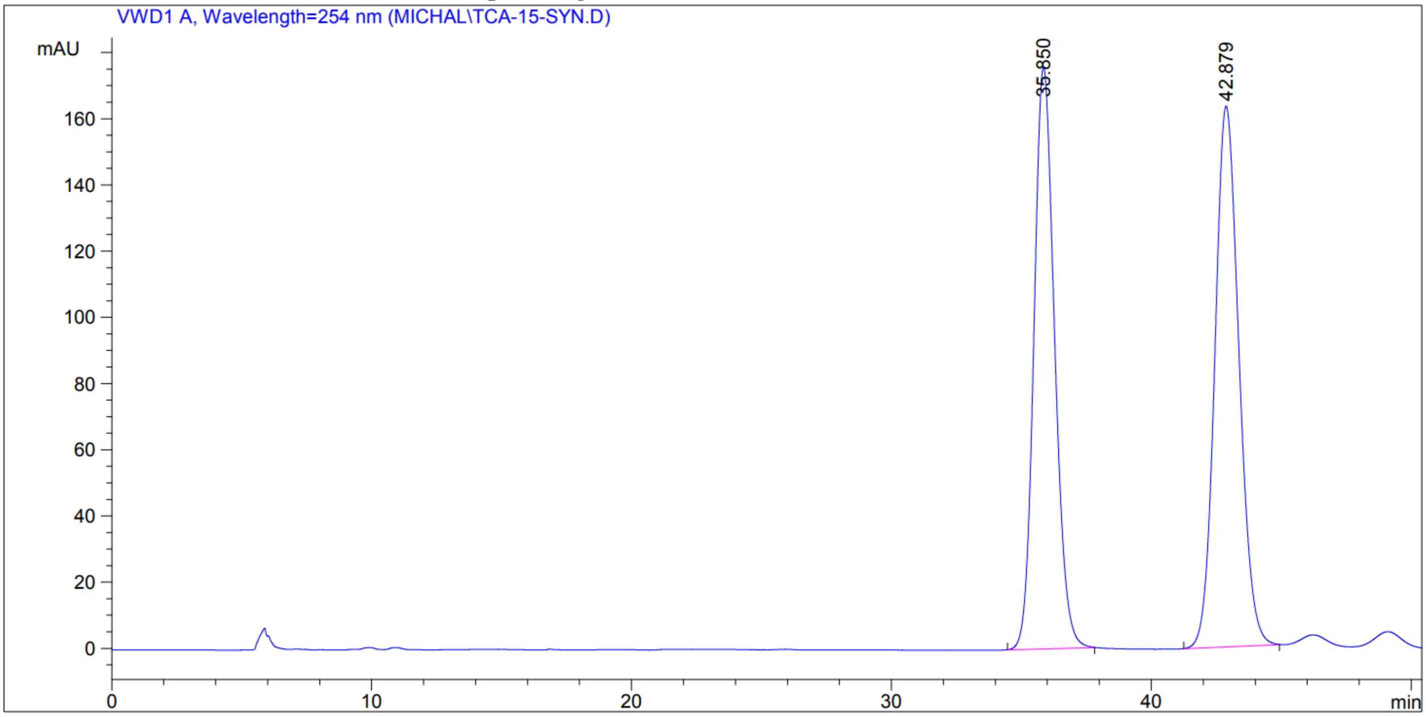


```
=====================================================================
                         Area Percent Report
=====================================================================

Sorted By             :      Signal
Multiplier:                   :       1.0000
Dilution:                     :       1.0000
Use Multiplier & Dilution Factor with ISTDs


Signal 1: VWD1 A, Wavelength=254 nm

Peak RetTime Type  Width     Area      Height     Area
  #   [min]        [min]   mAU   *s   [mAU   ]       %
----|-------|----|-------|----------|----------|--------|
   1  35.850 BB    0.8475 9680.69434  175.86061  47.9545
   2  42.879 BB    0.9946 1.05066e4   163.43764  52.0455

Totals :                  2.01873e4   339.29825


=====================================================================
                          *** End of Report ***
```

Figure S72. *HPLC analysis of racemic mixture of 3b and ent-3b.*

Data File C:\CHEM32\1\DATA\MICHAL\TCA-16-TC-462.D
Sample Name: IC, 90/10, 0.6ml, 12micoL, 254nm

===

Acq. Operator : tomas
Acq. Instrument : Instrument 1 Location : -
Injection Date : 1/19/2023 4:16:54 AM
Acq. Method : C:\CHEM32\1\METHODS\ROBO.M
Last changed : 1/19/2023 4:12:28 AM by tomas
(modified after loading)
Analysis Method : C:\CHEM32\1\METHODS\ROBO.M
Last changed : 1/19/2023 2:16:41 AM by tomas
(modified after loading)
Additional Info : Peak(s) manually integrated

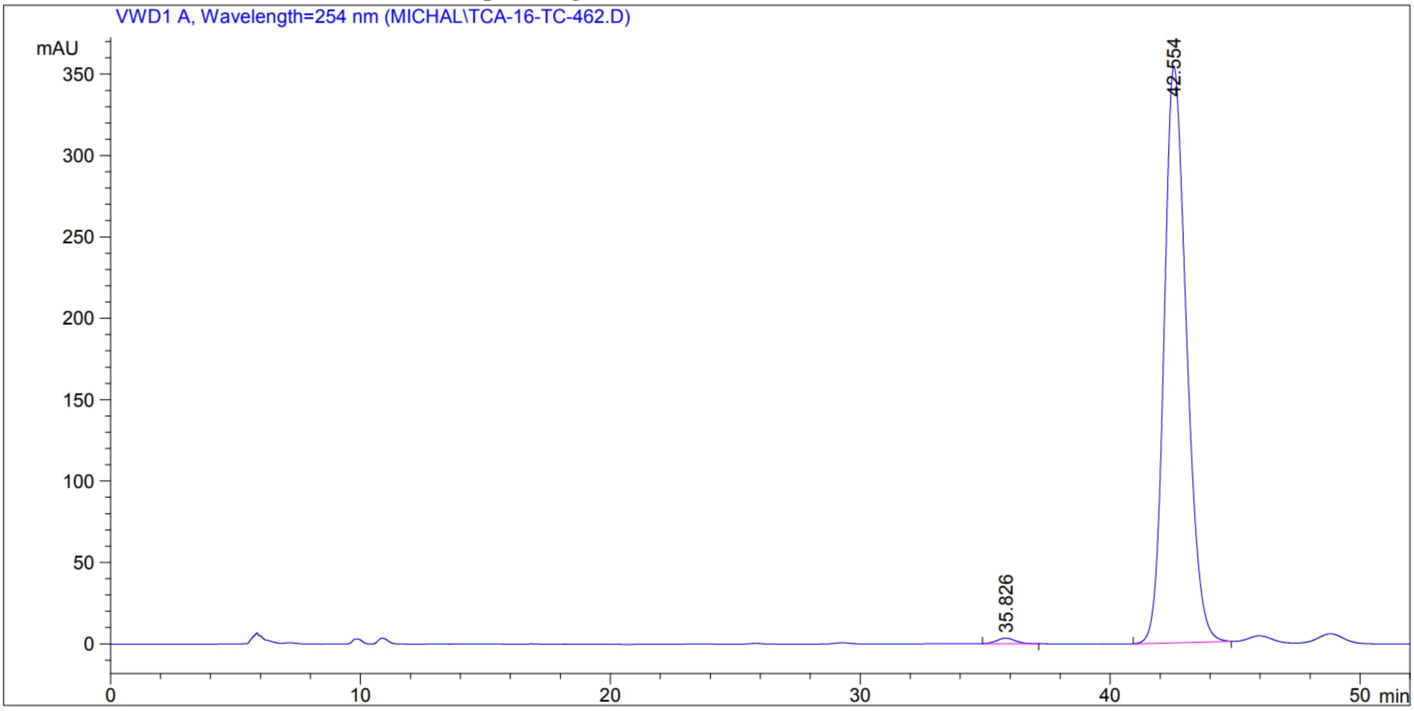

===

Area Percent Report

===

Sorted By : Signal
Multiplier: : 1.0000
Dilution: : 1.0000
Use Multiplier & Dilution Factor with ISTDs

Signal 1: VWD1 A, Wavelength=254 nm

Peak #	RetTime [min]	Type	Width [min]	Area mAU *s	Height [mAU]	Area %
1	35.826	BB	0.7033	174.66859	3.36409	0.7843
2	42.554	BB	0.9578	2.20968e4	354.25656	99.2157

Totals : 2.22715e4 357.62065

===

*** End of Report ***

Figure S73. *HPLC analysis of synthetic 3b.*
